# Discovery and population genomics of structural variation in a songbird genus

**DOI:** 10.1038/s41467-020-17195-4

**Published:** 2020-07-07

**Authors:** Matthias H. Weissensteiner, Ignas Bunikis, Ana Catalán, Kees-Jan Francoijs, Ulrich Knief, Wieland Heim, Valentina Peona, Saurabh D. Pophaly, Fritz J. Sedlazeck, Alexander Suh, Vera M. Warmuth, Jochen B. W. Wolf

**Affiliations:** 1 Department of Evolutionary Biology and Science for Life Laboratory, Uppsala University, 752 36 Uppsala, Sweden; 2 Division of Evolutionary Biology, Faculty of Biology, LMU Munich, Grosshaderner Str. 2, 82152 Planegg-Martinsried, Germany; 3 Uppsala Genome Center, Science for Life Laboratory, Department of Immunology, Genetics and Pathology, Uppsala University, BMC, Box 815, 752 37 Uppsala, Sweden; 4 BioNanoGenomics, San Diego, CA 92121 USA; 5 Institute of Landscsape Ecology, University of Münster, Heisenbergstrasse 2, 48149 Münster, Germany; 6 Human Genome Sequencing Center at Baylor College of Medicine, 1 Baylor Plaza, Houston, TX 77030 USA; 7 Present Address: Department of Biology, Pennsylvania State University, 310 Wartik Lab, University Park, PA 16802 USA; 8 Present Address: Department of Organismal Biology – Systematic Biology, Uppsala University, 752 36 Uppsala, Sweden; 9 Present Address: Max Planck Institute for Plant Breeding Research, Carl-von-Linné-Weg 10, 50829 Cologne, Germany; 10 Present Address: School of Biological Sciences, University of East Anglia, Norwich Research Park, Norwich, NR4 7TU UK

**Keywords:** Evolutionary genetics, Population genetics, Structural variation, Comparative genomics

## Abstract

Structural variation (SV) constitutes an important type of genetic mutations providing the raw material for evolution. Here, we uncover the genome-wide spectrum of intra- and interspecific SV segregating in natural populations of seven songbird species in the genus *Corvus*. Combining short-read (*N* = 127) and long-read re-sequencing (*N* = 31), as well as optical mapping (*N* = 16), we apply both assembly- and read mapping approaches to detect SV and characterize a total of 220,452 insertions, deletions and inversions. We exploit sampling across wide phylogenetic timescales to validate SV genotypes and assess the contribution of SV to evolutionary processes in an avian model of incipient speciation. We reveal an evolutionary young (~530,000 years) *cis*-acting 2.25-kb LTR retrotransposon insertion reducing expression of the *NDP* gene with consequences for premating isolation. Our results attest to the wealth and evolutionary significance of SV segregating in natural populations and highlight the need for reliable SV genotyping.

## Abstract (web summary)

Structural genomic variation can fuel evolution. Here, authors present genome data from seven *Corvus* species and unearth structural variants that vary between incipient crow species in Europe, with implications for premating isolation involving plumage patterning.

## Introduction

Structural mutations altering more than 50 bp of DNA sequence at once include deletions, (transposable) insertions, inversions, duplications and translocations, and have the potential to drastically change phenotypes with medical and evolutionary implications^1–4^. Yet, technological constraints have long impeded genome-wide characterization of structural mutations segregating in populations (structural variation, hereafter referred to as SV)^5^. This is because detection of SV requires highly contiguous genome assemblies accurately representing the repetitive fraction of genomes which is known to be a vibrant source and catalyst of SV^6–8^. SV likely remains hidden unless sequence reads traverse its entire length^9,10^. As a consequence, despite the rapidly increasing number of short-read (SR)-based genome assemblies^11^ and associated population genomic investigations, genome-scale SV generally remains largely unexplored^12^. Even in genetic model organisms, population-level analysis of different classes of SV has been restricted to pedigrees^13^ or organisms with smaller, less complex genomes^14,15^, and few studies have provided a comprehensive account of SV segregating in natural populations^15,16^. Despite the technical difficulty for genome-wide and population-wide identification and reliable genotyping, SV is believed to be a major contributor to processes of population divergence and speciation^12,17^.

**Fig. 1:**
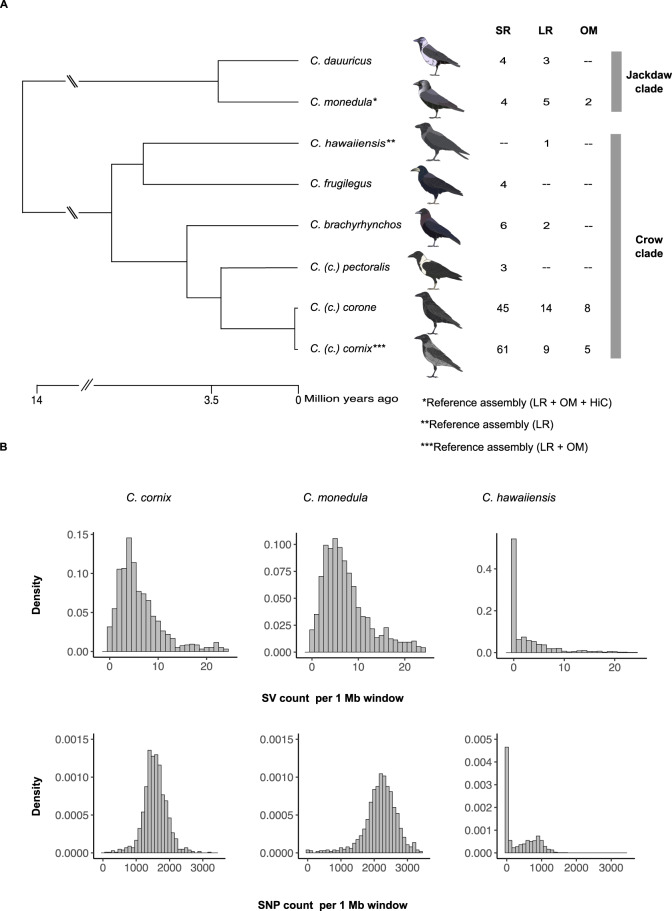
Sampling setup and assembly-based structural and single-nucleotide variation. **a** Phylogeny of sampled species in the genus *Corvus* (after, see ref. ^18^). Numbers in columns represent individual numbers for short-read sequencing (SR), long-read sequencing (LR), and optical mapping (OM). **b** Density histogram showing the abundance of genetic variation within single individuals. Counts of variants per 1 Mb windows are based on comparing the two haplotypes of each assembly. The upper panel reflects structural variation (SV) densities, the lower panel reflects densities for SNPs. Crow drawings in 1A by Alice Meröndun, published under a CC BY-SA 4.0 license (https://creativecommons.org/licenses/by-sa/4.0/deed.en). No changes were made to the original drawings. Source data are provided as Source Data file.

The avian songbird genus *Corvus* comprises a total of 40 species including crows, ravens, and jackdaws^18^. Central to this system is the phylogenetically independent recurrence of a pied color-pattern in several species that stands in contrast to the predominant all-black plumage in the clade (Fig. 1a). Color divergence is believed to contribute to prezygotic isolation by means of social and sexual selection promoting speciation^19^. The genetic basis of plumage variation and its contribution to population divergence has been investigated in much detail using single-nucleotide polymorphism (SNP) data in the *Corvus (corone) spp*. species complex that is characterized by narrow contact zones between all-black forms (*C. (c.) corone/orientalis*) and populations consisting of the pied phenotype (*C. (c.) cornix/capellanus/pallescens/pectoralis/sharpii*)^20–22^. These studies suggest that phenotypically well differentiated populations diverged during the late Pleistocene and exhibit low levels of genome-wide differentiation^23^. Only few narrow genomic regions associated with morphological contrast are subject to divergent selection reducing local gene flow and increasing genetic differentiation^23,24^. In the European hybrid zone comprising carrion crows (*C. (c.) corone*) and hooded crows (*C. (c.) cornix*) epistasis between a genetic factor on chromosome 18 and the gene *NDP* located on chromsome 1 explained most of the morphological variation^25^, which can be near-exclusively related to gene expression differences in melanocytes of nascent feathers^26,27^. Despite some indication for a structural rearrangement underlying phenotypic divergence in two European crow populations^24^, the overall extent and role of SV in population divergence remains unclear in this system.

To fill this gap and more broadly investigate the dynamics of SV in natural populations, we compiled short-read, long-read, and optical mapping data for populations of seven species from the genus *Corvus*. We generated genome assemblies for two of the species spanning the evolutionary history of the clade, comprehensively characterized SV using read-mapping and assembly-based approaches and used phylogenetically informed filtering to obtain reliable SV genotypes. After establishing these genomic resources, we demonstrated the suitability of SV for population genetic analysis and evolutionary inference using the *Corvus (corone)* spp. species complex.

## Results

### Assembly-based SV

We first generated high-quality phased de novo genome assemblies combining long-read (LR) data from single-molecule, real-time (SMRT, PacBio) sequencing, and nanochannel optical mapping (OM) for the hooded crow (*Corvus (corone) cornix*; data from Weissensteiner et al.^28^), and the European jackdaw (*Corvus monedula*, this study). For the former, we also generated chromatin interaction mapping data (Hi-C) to obtain a chromosome-level reference genome (Fig. 1a, see Supplementary Fig. 1 for approach and Supplementary Table 1 for assembly statistics). In addition, we included a previously published LR assembly of the Hawaiian crow (*Corvus hawaiiensis*) in the analyses^29^. All assemblies were generated with the diploid-aware FALCON-UNZIP assembler^30^, facilitating the comparison of haplotypes within species to identify heterozygous variants and determine genetic diversity at the level of single individuals. After aligning the two haplotypes of each assembly, we identified SNPs, insertions and deletions in all three species (Table 1, Supplementary Fig. 2). Median sizes of SVs ranged from 111 bp in jackdaw to 158 bp in Hawaiian crow, with all three species showing similar SV length distributions (Supplementary Fig. 3). As a proxy for genetic diversity within a single individual, we recorded genome-wide densities of SNPs and non-overlapping SVs in 1 Mb windows (Fig. 1b). Total as well as per-window numbers of SV and SNPs were highest in jackdaw and lowest in the highly inbred Hawaiian crow (across four SV size categories, Supplementary Fig. 4), consistent with a positive correlation between census population size and genetic diversity^31,32^.

**Table 1:** Assembly-based structural variation and single-nucleotide polymorphism detection.

Species	Total nr. of SNP	Mean SNP density per 1 Mb	Median SNP density per 1 Mb	Total nr. of SV	Mean SV density per 1 Mb	Median SV density per 1 Mb
Hooded crow	1,637,609	1568	1558	9916	9.19	5
Jackdaw	2,262,079	2189	2228	9903	9.29	7
Hawaiian crow	414,229	366	0	4841	3.82	0

### Long-read resequencing and phylogenetically informed filtering

**Fig. 2:**
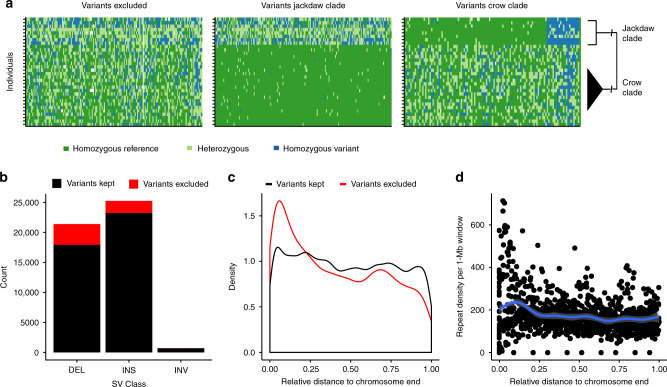
Phylogenetic filtering of read mapping-based structural variants. **a** Example genotype plots of LR-based variants according to phylogenetically informed filtering. Given the large divergence time of 13 million years^18^ between the crow and jackdaw lineage, the proportion of polymorphisms shared by descent is negligible^62^ and therefore likely constitutes false positives or hypermutable sites (left). Variants segregating exclusively in the jacdaw or crow clade (middle and right), however, comply with the infinite sites model and were retained accordingly. Plotted are genotypes of one representative chromosome (chromosome 18), with genotypes of variants in different colors, where each row corresponds to one individual (*N* = 8 individuals jackdaw clade and *N* = 24 individuals crow clade). Note that, due to the tolerance of a certain number of misgenotyped variants per clade, some variants are present in both clades. **b** Excluded versus retained variants in relation to SV class and chromosomal distribution. Excluded variants are enriched for deletions (LMM, *p* < 10^−16^) and **c** are most abundant at chromosome ends, coinciding with **d**, an increased repeat density. Source data are provided as Source Data file.

To uncover SV segregating within and between natural populations, we generated PacBio LR resequencing data for 31 individuals. Spanning the phylogeny of the genus, this dataset included samples from the European and Daurian jackdaw (*C. monedula, C. dauuricus*), the American crow (*C. brachyrhynchos*), and the Eurasian crow complex (*C. (corone)* spp.). The latter comprised individuals from the phenotypically divergent hooded crow (Sweden and Poland), and carrion crow populations (Spain and Germany)^23^ (Fig. 1a). Individuals were sequenced to a mean sequence coverage of 15 (range 8.47–27.91) with a mean read length of 7535 bp (range 5219–10,034 bp; Supplementary Table 2). Mapping reads to the hooded crow reference allowed us to identify variants and genotypes for each diploid individual, which resulted in a set of 47,346 variants (see Supplementary Fig. 5 for SV detection approach). SV genotyping is nontrivial and associated with high uncertainty^33^. Thus, we utilized the multispecies sampling scheme to filter for variants complying with basic population genetic assumptions (Fig. 2a, see “Methods” section). Variants that were excluded according to these criteria contained more deletions and clustered near the end of chromosomes (linear model, *p* = 10^−16^, Fig. 2b, c). Increased densities of repetitive elements (Fig. 2d), particularly tandem repeat arrays and clusters of interspersed repeats, in these regions are conducive to erroneous genotype calling, though it is possible that a subset of these phylogenetically recurring variants indeed represent true positive, hypermutable sites.

**Fig. 3:**
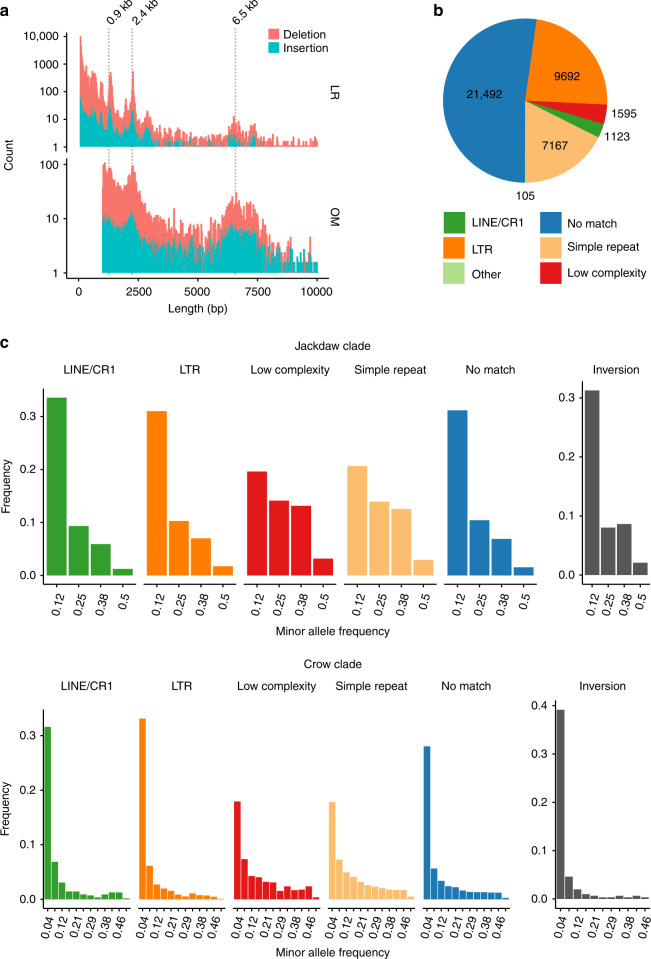
Characterization and allele frequencies of SV. **a** Length distributions of deletions and insertions shorter than 10 kb identified with LR (upper panel) and OM (lower panel) data. Pronounced peaks at 0.9, 2.4 kb in the LR and at 2.4 and 6.5 kb in the OM variants likely stem from an overrepresentation of specific repeats. Indeed, among the five most common repeats found in insertions and deletions are LTR retrotransposons with a consensus sequence length of 670, 1315, 2072 bp, respectively. **b** Content of insertion and deletion sequences. About half of all variants were assigned to a known repeat family, of which transposable elements from the LTR retrotransposon subclass were most common, followed by simple repeats (including microsatellites) and low complexity repeats. **c** Folded allele frequency spectra of structural variants. Upper and lower panels correspond to the jackdaw and crow clade, respectively. The five left panels depict the minor allele frequencies of insertions and deletions, and the rightmost panel that of inversions. Source data are provided as Source Data file.

After the phylogenetically informed filtering step, we retained a final set of 41,868 variants (88.43% of the initial, unfiltered set) segregating within and between species. Of these, a small proportion was classified as inversions (694, 1.65%), whereas the vast majority was attributed to insertions (23,235, 55.49%) and deletions (17,939, 42.84%) relative to the hooded crow reference. Variant sizes were largest for inversions, with a median size of 980 bp (range: 51–99,824 bp), followed by insertions (248 bp, range: 51–45,373 bp) and deletions (154 bp, range: 51–94,167 bp). The latter showed noticeable peaks in the size distribution at around 900, 2400, and 6500 bp (Fig. 3a, for inversions see Supplementary Fig. 6), which likely stem from an overrepresentation of paralogous repetitive elements. The three most common repeat types in insertions and deletions belonged to endogenous retrovirus-like long terminal repeats (LTR) retrotransposon families and accounted for 13.89% of all matches to a manually curated repeat library (Supplementary Table 3). This suggests recent activity of this transposable element group, as has been previously reported in population-level analyses of other songbird species^34^. More than half of all insertions and deletions could not be associated with any annotated repetitive element family (52.19%). The remainder was distributed approximately equally between tandem repeats (e.g., simple and low complexity repeats) and interspersed repeats. The latter category was dominated by LTR and LINE/CR retrotransposons with only a small number of SINE retrotransposons (Fig. 3b, Table 2). These different types of repeat elements exhibit fundamentally different mutation mechanisms^35^ and effects on neighboring genes^36,37^, such that repeat annotations are of crucial importance for the downstream population genetic analysis of SV.

**Table 2:** Characterization of LR insertions and deletions using RepeatMasker.

Classification	Number	Percentage
Tandem repeat total	8847	21.48
Simple repeat	7167	17.4
Low complexity repeat	1595	3.87
Satellite	75	0.18
rRNA	5	<0.05
tRNA	3	<0.05
Macrosatellite	1	<0.05
Interspersed repeat total	10,828	26.3
LTR retrotransposon	9692	23.53
LINE/CR1 retrotransposon	1123	2.27
SINE retrotransposon	11	<0.05
DNA/hAT-Charlie element	2	<0.05
No match	21,492	52.19

### Allele frequency spectra of long-read sequencing-based SV

For population genetic analyses, we considered structural variation segregating within clades sharing recent common ancestry. A total of 35,723 and 29,555 variants remained after filtering in the jackdaw (*N* = 8 individuals; *C. monedula, C. dauuricus*) and crow clade (*N* = 24; *C. (corone) spp., C. brachyrhynchos*), respectively. Using the full data set across all populations within each clade allowed us to compare folded allele frequency spectra between SV classes and repetitive element types with high resolution (for population specific spectra unbiased by population structure see Supplementary Fig. 7). Consistent with recent studies in grapevine and *Drosophila* SV^15,38^, the distribution of allele frequencies was skewed towards rare alleles (Fig. 3c). However, allele frequency spectra of different SV classes differed in shape. While insertions and deletions associated with LTR retrotransposons, LINE/CR1 retrotransposons or without any match to a known repeat type as well as inversions exhibited the typical pattern of a strongly right-skewed frequency distribution, allele frequencies of simple and low complexity repeats were shifted towards intermediate frequencies. Besides a potential technical bias due to the more difficult genotyping and variant discovery of these classes^39^, this pattern is consistent with convergence to intermediate allele frequencies due to high mutation rates^35^. These results illustrate how different underlying mutation dynamics potentially impact the analysis of population genetic parameters for SV.

### Optical mapping-based SV detection

To improve our ability to detect larger SV and to provide an independent orthogonal approach for SV discovery, we generated 14 optical maps (Fig. 1a) and compared them, together with two additional optical maps taken from Weissensteiner et al.^28^, to the hooded crow reference assembly. Following that approach, we identified 12,807 insertions, 8799 deletions and 293 inversions. As expected from the increased size of individually assessed DNA molecules (mean molecule N50 = 223.38 kb), variants identified with this approach exhibited a different size range (Fig. 3a) after applying the same upper limit (100 kb) as for the LR SV calls and a lower limit of resolution (1 kb)^40^. Insertions and deletions were not only enriched at lengths around 0.9 and 2.4 kb as seen in the LR-based SV calling, but also at ~6.5 kb, likely corresponding to full-length insertions of the LTR retrotransposons in this size range. Thus, independent approaches targeting different size ranges of SV are vital to increase sensitivity in detecting hidden genetic variation.

### Short-read sequencing-based SV detection

To increase our sample size and expand our analysis to further populations and species (Fig. 1a), we applied a consensus approach of three different short-read (SR) based SV detection approaches on previously published data of 127 individuals^23,24^. In total, we identified 132,025 variants of which 97,524 (73.87%) were unique to single individuals. In total, only 11,951 variants overlapped with the final set of variants identified in the LR data set (corresponding to 9.05% of SR and 28.54% LR calls). This disconnect cannot be explained solely by differences in sample size. More likely, it indicates a high number of false-positives and false-negatives in the SR-based approach known for its sensitivity to the calling method^41^ and disparity to LR-based calls^10,14^. Therefore, we focused on the LR-based SV calls in the subsequent analysis and considered carefully selected SR calls only for specific mutations.

### Population genetics of long-read sequencing-based SV

**Fig. 4:**
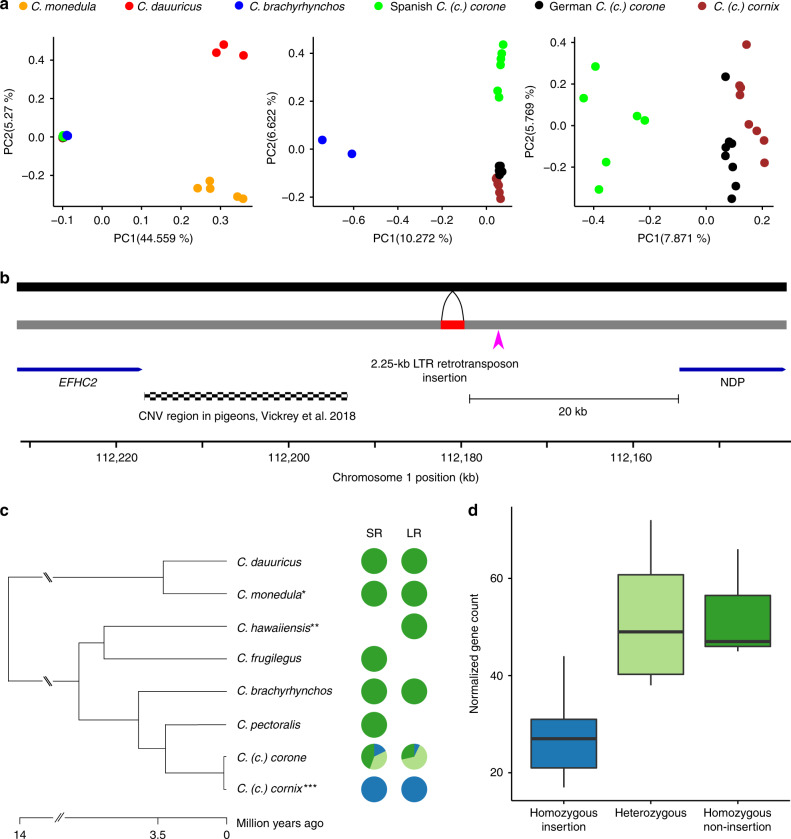
SV-based population structure and LTR retrotransposon insertion upstream of the *NDP* gene. **a** Principal component analysis based on SV genotypes. In the left, all individuals were analyzed together. Individuals of the crow clade are tightly clustered and separate from the jackdaw clade along PC1; individuals of the two jackdaw species separate along PC2. The middle displays the results of PCA conducted exclusively for individuals from the crow clade, clearly separating American crows from the Eurasian species. In the right, only the European populations of the crow clade are included, showing marked separation of the Spanish carrion crow population. **b** A 2.25-kb LTR retrotransposon insertion into the crow lineage (black bar: ancestral state, gray bar: derived, reference allele) belongs to the endogenous retrovirus K (ERVK) subfamily corCorLTRK1b and is located 20 kb upstream of the *NDP* gene. A highly conserved non-coding region (pink arrow) is present in close proximity (2.8 kb) to the insertion in the 3′ flanking sequence. This region, which is conserved between chicken, human, and crow (Supplementary Fig. 8), is likely a regulatory element which may be affected by the nearby LTR retrotransposon insertion. Located in the 5′ region of the insertion is a region copy number variable in pigeons, associated with plumage pattern variation. **c** Genotypes of the LTR insertion in short-read (SR) and long-read (LR) data. In both datasets, the LTR element insertion (blue) is fixed in all hooded crow populations. Species and populations with a black plumage are either polymorphic (light green) or fixed for the ancestral state, non-insertion (green). **d** Gene expression of *NDP* in body skin. Normalized gene counts of 18 individuals are significantly associated with the insertion genotypes (LMM, *p* = 0.002), boxplot center lines show median show medians and whiskers 1.5 times the interquartile range (*n* = 19). Source data are provided as Source Data file.

Next, we investigated population structure using principal component analyses (PCA). Figure 4a (based on LR data) recapitulates the pattern of population stratification found in Vijay et al.^23^ based on 16.6 million SNPs, and thus supports the general suitability of SV genotypes for population genetic analyses (for SR data see Supplementary Fig. 8). In order to identify SV associated with prezygotic reproductive isolation, we calculated genetic differentiation between phenotypically divergent populations connected by gene flow^23,24^ and allopatric populations within the same phenotype^23^. Mean *F*_ST_ was low overall with values ranging from 0.03 in the hooded versus carrion crow comparison to 0.15 in the hooded versus American crow comparison.

A total of 103 variants fell into the 99th percentile of *F*_ST_ in the gray-coated hooded versus all-black carrion crow population comparison in central Europe. These variants, located on 23 chromosomes, were considered as ad hoc candidate outlier loci subject to divergent selection^12^, and were found at a median distance of 14.32 kb to adjacent genes (range: 0–695.84 kb). (Supplementary Table 4). Ten of these outliers (10.31%) were placed on chromosome 18, which was previously identified by SNP-based analyses as a candidate genomic region subject to divergent selection between the taxa^23–25^. Chromosome 18 only represents 1.22% of the entire assembly, corresponding to an ~8.5-fold enrichment of highly differentiated SV. Given that outliers are located in the proximity of previously identified genes presumably under divergent selection (such as *AXIN2* and RGS9, Supplementary Table 4), this supports a crucial role of chromosome 18 in maintaining plumage divergence^24,25^.

The three highest F_ST_ outliers included an 86 bp indel on chromosome 18 inside of a tandem repeat array, a 1.56 kb indel on chromosome 3 and a 2.25 kb indel on chromosome 1 (Supplementary Table 5). The latter, an LTR retrotransposon insertion, was located 20 kb upstream of the *NDP* gene on chromosome 1 (Fig. 4b), a gene known to contribute to the maintenance of color divergence across the European crow hybrid zone^25,26^. Molecular dating based on the LTR region suggested an insertion event at <534,000 years ago upon diversification of the European crow lineage (Fig. 4c)^23^. In current day populations, the insertion still segregates in all-black crows including *C (c.) corone* in Europe and *C (c.) orientalis* in Russia (*N* individuals with LR = 14 and with SR = 45 genotypes) (Fig. 4c). However, all hooded crow *C. (c.) cornix* individuals genotyped with LR (*N* = 9) and SR data (*N* = 61) were homozygous for the insertion regardless of their population of origin. This finding is consistent with a selective sweep in proximity to the *NDP* gene that has previously been suggested in hooded crow populations^24,25^. Recent work has also shown that the *NDP* gene exhibits decreased gene expression in gray feather follicles of hooded crows, suggesting a role in modulating overall plumage color patterning^26^. Following re-analysis of normalized gene expression data for eight carrion and ten hooded crows^26^, we found a significant association between the homozygous insertion genotype and decreased *NDP* gene expression levels in body skin tissue (linear model, *p* = 0.002) (Fig. 4d), in line with reduced eumelanin pigmentation in hooded crows^27^.

To further investigate the relationship between the abovementioned LTR insertion and phenotypic differences between all-black *C (c.) corone* and gray-coated *C. (c.) cornix* populations, we genotyped 120 individuals from the European hybrid zone using PCR^25^. Including data of SNPs for the same individuals, we tested the association between genotype and pigmentation phenotype. A statistical model including the insertion fit best to the observed phenotypes (ΔAICc = 2.33, but ΔBIC = −0.12), explaining an additional 10.32% of the variance of the phenotype-derived PC1 relative to the adjacent SNPs. The insertion lies upstream of *NDP* in close proximity (2.8 kb) to a highly conserved non-coding region in vertebrate genomes (likely constituting a regulatory element, Supplementary Fig. 9) as well as an orthologous region in pigeons where a copy number variation modulates plumage patterning (Fig. 4b)^42^. Reminiscent of the color altering TE insertions in butterflies^3,43^, this insertion thus constitutes a prime candidate for a causal mutation modulating gene expression with phenotypic consequences. While such insertions have usually been associated with increased expression of the affected gene^36^, there are also examples of TE insertions repressing gene activity^44–46^, as observed here.

## Discussion

SV constitutes a large proportion of genetic variation in the genomes of eukaryote organisms^47^ affecting phenotypes with fitness consequences^1^. It is thus imperative to investigate the extent and occurrence of SV beyond well-studied model organisms and characterize its dynamics in naturally evolving populations. The study presented here illustrates the feasibility of SV characterization followed by downstream population analyses in a non-model organism without reliance on any prior genetic resources.

The mere comparison of de novo assembled haplotypes from single diploid individuals in several species revealed a positive correlation of SV with census population size^31,32^ and confirmed a high degree of inbreeding in the Hawaiian crow^29^. Moreover, using population samples across several species and combining sequencing technologies, mapping strategies and computational inference methods proved particularly useful in characterizing different types and sizes of SV across the genome^47^. For example, insertions and deletions identified with optical mapping exhibited a notable peak in their size distribution around 6.5 kb, which was neither evident in LR-based nor SR-based SV detection. This finding, which indicates an increased abundance of LTR retrotransposon insertions of a certain size, illustrates the necessity of adding complementary data to uncover the full size spectrum of SV. The poor consistency of SV identified with SR and LR data, which appears to be commonplace even in much smaller, less complex genomes^14^, cautions against interpetation of SV calls solely from SR data known to exhibit difficulties in capturing the complexity of repetitive sequence. Yet, also for LR data, reliable and accurate determination of the genotype of a given variant is paramount for meaningful downstream analysis. While there are numerous tools for SV discovery for any given technology (reviewed in the ref. ^47^), approaches for genotyping SV are still in their infancy^33^. As a workaround, we validated SV genotypes using population genetic reasoning based on the infinite sites model in a phylogenetic setting. While this approach likely excludes hypermutable sites such as microsatellites, it helped minimize false-positives, and constitutes a first step toward a more reliable genotyping readily applicable to other systems.

The filtered set of variants then allowed construction of allele frequency spectra forming the basis of evolutionary downstream analyses. A 2.25-kb LTR retrotransposon insertion on chromosome 1 emerged as a key candidate contributing to the striking contrast in plumage variation between European crow populations^23,24^. Transposable elements are well-known modulators of gene expression^36,44,46,48^, and in fact the homozyogous insertion of this retrovirus-like LTR retrotransposon was associated with decreased gene expression of the *NDP* gene affecting plumage patterns. Furthermore, the inclusion of the LTR insertion genotype in an association-mapping analysis of phenotypic hybrid crow individuals from a previous study^25^ explained more variation than any SNP. Taken together, these results render this LTR retrotransposon insertion a prime candidate mutation underlying plumage divergence in hooded and carrion crows, and add to the notion that transposable elements are important contributors to the genetic architecture of phenotypic change^3,43^. Given the importance of color variation to prezygotic isolation in this system^21,25,49^ it constitutes an example of a structural mutation with evolutionary significance. Since the majority of SV is likely still uncovered in most organisms^50^, these results represent an important hallmark for the field, highlighting the need for rigorous methodological approaches and the evolutionary importance of SV in natural populations.

## Methods

### DNA extraction and long-read sequencing

We extracted high-molecular weight DNA from a total of 32 samples using either a modified phenol–chloroform extraction protocol^28^, or the Qiagen Genomic-tip kit (following manufacturer’s instructions) from frozen blood samples. For sampling details, see Supplementary Table 5. Extracted DNA was eluted in 10 mM Tris buffer and stored at −80 °C. The quality and concentration of the DNA was assessed using a 0.5% agarose gel (run for >8 h at 25 V) and a Nanodrop spectrophotometer (ThermoFisherScientific). Long-read sequencing DNA libraries were prepared using the SMRTbell Template Prep Kit 1.0 (Pacific Biosciences). For each library, 10 µg genomic DNA was sheared into 20-kb fragments with the Hydroshear (ThermoFisherScientific) instrument. SMRTbell libraries for circular consensus sequencing were generated after an Exo VII treatment, DNA damage repair, and end-repair before ligation of hairpin adapters. Following an exonuclease treatment and PB AMPure bead wash, libraries were size-selected using the BluePippin system with a minimum cutoff value of 8500 bp. All libraries were then sequenced on either the RSII or Sequel instrument from Pacific Biosciences, totaling 324 RSII and 67 Sequel SMRT cells, respectively, resulting in 754 Gbp of raw data.

### Short-read sequencing data

We compiled raw short-read sequencing data from Poelstra et al.^24^ and Vijay et al.^23^ for *Corvus (corone)* spp., *C. frugilegus, C. dauuricus*, *C. monedula*, and *C. brachyrhynchos* (for more information on the origin of samples see Supplementary Table 5). Overall, 127 individuals had on average 12.6-fold sequencing coverage using paired-end libraries (primarily) sequenced on an Illumina HiSeq2000 machine.

### Genome assembly

In birds, females are the heterogametic sex (ZW). For this study, we were interested in a high-quality assembly of all autosomes and the shared sex chromosome (Z) and accordingly chose male individuals for the genome assemblies. Note, however, that this choice excludes the female-specific W chromosome a priori. Diploid genome assembly was performed for both a hooded crow and a jackdaw individual. For the former, a long-read-based genome assembly has previously been published^28^ and is available under the accession number (GCA_002023255.2) at the repository of the National Center for Biotechnology Information (NCBI, www.ncbi.nlm.nih.gov). Here, we (re)assembled raw reads using updated filtering and assembly software. First, all SMRT cells for the respective individuals (102 for the hooded crow individual S_Up_H32, 70 for the jackdaw individual S_Up_J01) were imported into the SMRT Analysis software suite (v2.3.0). Subreads shorter than 500 bp or with a quality (QV) < 80 were filtered out. The resulting data sets were used for de novo assembly with FALCON UNZIP v0.4.0^30^. Initial FALCON UNZIP assemblies of hooded crow and jackdaw consisted of primary and associated contigs with a total length of 1053.37 and 965.95 Mb for the hooded crow and 1,073.84 and 1,092.55 Mb for the jackdaw, presumably corresponding to the two chromosomal haplotypes (for assembly statistics see Supplementary Table 1). To further improve the assembly, we performed consensus calling of individual bases using ARROW^30^. In addition, we obtained the genome of the Hawaiian crow (*Corvus hawaiiensis*) from the repository of NCBI with accession number GCA_003402825.1 (https://www.ncbi.nlm.nih.gov/assembly/GCA_003402825.1). This genome had been likewise derived from long-reads generated with the SMRT technology and assembled using FALCON UNZIP^30^. To assess the completeness of the newly assembled genomes we used BUSCO v2.0.1^51^. The Aves and the vertebrate databases were used to indentify core orthologous gene sets (Supplementary Table 1).

### Optical mapping data and assembly

We generated additional optical map assemblies for two jackdaw individuals, eight carrion crow individuals and four hooded crow individuals, and reassembled single-molecule data for the hooded and carrion crow reported in Weissensteiner et al.^28^, following the approach described therein. In brief, we extracted nuclei of red blood cells and captured them in low-melting point agarose plugs. DNA extraction was followed by melting and digesting of the agarose resulting in a high-molecular weight DNA solution. After digestion with a nicking endonuclease (Nt.BspQI) which inserts a fluorescently labeled nick strand, the sample was loaded onto an IrysChip, which was followed by fluorescent label detection on the Irys instrument. The single molecule maps were assembled into consensus maps with the Bionano Access 1.3.1 Bionano Solve pipeline 3.3.1 (pipeline version 7841). As reference, an in silico map of the hooded crow reference assembly was used. Molecule and assembly statistics of optical maps can be found in Supplementary Table 6. For details regarding the hybrid scaffolding see Weissensteiner et al.^23^.

### Hi-C chromatin interaction mapping and scaffolding

One Dovetail Hi-C library was prepared from a hooded crow sample following Lieberman-Aiden et al.^52^. In brief, chromatin was fixed in place with formaldehyde in the nucleus and extracted thereafter. Fixed chromatin was digested with DpnII, the 5′ overhangs filled with biotinylated nucleotides and free blunt ends were ligated. After ligation, crosslinks were reversed and the DNA purified from the protein. Purified DNA was treated such that all biotin was removed that was not internal to ligated fragments. The DNA was then sheared to ~350 bp mean fragment size and sequencing libraries were generated using NEBNext Ultra enzymes and Illumina-compatible adapters. Biotin-containing fragments were isolated using streptavidin beads before PCR enrichment of each library. The library was then sequenced on an Illumina HiSeq X (rapid run mode). The Dovetail Hi-C library reads and the contigs of the primary FALCON UNZIP assembly were used as input data for HiRise, a software pipeline designed specifically for using proximity ligation data to scaffold genome assemblies^53^. An iterative analysis was conducted. First, Hi-C library sequences were aligned to the draft input assembly using a modified SNAP read mapper (http://snap.cs.berkeley.edu). The separation of read pairs mapped within draft scaffolds were analyzed by HiRise to produce a likelihood model for genomic distance between read pairs, and the model was used to identify and break putative misjoins, to score prospective joins and make joins above a threshold. The resulting 48 super-scaffolds were assigned to 27 chromosomes based on synteny to the flycatcher genome version (NCBI accession GCA_000247815.2)^54^ using LASTZ^55^. The resulting Hi-C scaffolded hooded crow assembly is available as a Dryad repository, file 10.5061/dryad.ns1rn8ppj.

### Assembly-based SV and SNP detection

We aligned the associated contigs of all three assemblies (hooded crow, jackdaw and Hawaiian crow) to the primary contigs (super-scaffolded to chromosome level for hooded crow) using MUMmer^56^. SNPs were then identified using *show-snps* with the options –Clr and –T following a filtering step with delta-filter –r and –q. We only considered single-nucleotide differences in this analysis.

Structural variants between the two haplotypes of each assembly were identified using two independent approaches (Supplementary Fig. 2). First, we used the alignments produced with MUMmer to identify variants using the Assemblytics tool^57^. We then converted the output to a *vcf* file using SURVIVOR (v1.0.3)^41^. Independently, we used the smartie-sv pipeline to identify structural variants^58^, and then converted and merged the output with the Assemblytics-based variant set with SURVIVOR. This final unified variant set was then used to calculate SV-density in non-overlapping 1-Mb windows.

### Read-mapping-based SV detection

We aligned PacBio long-read data of all re-sequenced individuals to the hooded crow reference assembly using NGM-LR^59^ (v0.2.2) with the –pacbio option and sorted and indexed resulting alignments with samtools^60^ (v1.9) (Supplementary Fig. 5). Initial SV calling per individual was then performed using Sniffles^59^ (v1.0.8) with parameters set to a minimum support of 5 reads per variant (–min_support 5) and enabled –genotype, –cluster and –report_seq options. We removed abundant translocation calls indicative of an excess of false positives and filtered remaining variants for a maximum length of 100 kb and a maximum read support of 60 with bcftools^61^. Both of these filtering steps have been shown to be necessary to remove erroneously called variants. Next, we generated a merged multi-sample *vcf* file consisting of all individuals from both the crow and the jackdaw clades with SURVIVOR merge and options set to 1000 1 1 0 0 50. This merged *vcf* file was then used as an input to reiterate SV calling with Sniffles for each individual with the –Ivcf option enabled, effectively genotyping each variant per individual (Supplementary Data 1). Resulting single individual *vcf* files were again merged with the SURVIVOR command described above and variants overlapping with assembly gaps were removed. We converted the *vcf* file into a genotype file with vcftools^61^ (v0.1.15) for downstream analysis.

To account for the high amount of genotyping errors and false positives after initial filtering, we employed a “phylogenetic” filtering strategy. The crow and jackdaw clades diverged roughly 13 million years ago^18^, such that the proportion of polymorphisms shared by descent is near negligible^62^. Moreover, under the infinite sites model, recurrent mutations are not expected, therefore polymorphisms segregating in both lineages most likely constitute false positives. For population genetic analyses of the jackdaw clade, we therefore considered only variants which were homozygous for the reference in crow clade individuals, allowing for a maximum of four genotyping errors. In the crow clade analyses, we only retained variants which were either fixed for the reference or the variant allele in the jackdaw clade, allowing for two genotyping errors. It is likely that this conservative approach excludes variants with a high mutation rate^63^. However, since it is difficult to differentiate such variants from genotyping errors, we deemed this filter necessary to yield a set of reliable variants. Due to the tolerance of genotyping errors, there is a number of variants present in both clades, most of them fixed or almost fixed in both clades. Extensive manual curation would be necessary to differentiate between genotyping errors and variants truly polymorphic between clades. To find common features in filtered versus kept variants, we applied a generalized linear mixed-effects model with a binomial error structure, in which we coded the dependent variable as 1 for a retained variant and as 0 for a filtered variant. As covariates we included the distance to the chromosome end and variant class as a factor (insertion, deletion or inversion). We further fitted chromosome identity as a random intercept term. All models were run in R (v3.2.3, R Core Team) using the lme4 package^64^ (v1.1–19).

The short-read data were mapped using BWA-MEM with the –M option to the hooded crow reference assembly^65^. We used LUMPY^66^, DELLY^67^, and Manta^68^ to obtain SV calls for each sample using their respective default parameters. Subsequently the individual SV calls per sample were merged using SURVIVOR merge^41^ with the parameters: “1000 2 1 0 0 0”. This filtering step retained only SV calls for which 2 out of the 3 callers had reported a call within 1 kb. Next, we computed the coverage of low mapping quality reads (MQ < 5) for each sample independently and recorded regions where the low MQ coverage exceeded 10. SV calls which overlapped these regions were filtered out (Supplementary Data 2).

### Repeat annotation and characterization of insertions and deletions

To characterize the repeat content of the hooded crow assembly, we further curated the repeat library from Vijay et al.^23^. Raw consensus sequences were manually curated following the method used in Suh et al.^34^. Every consensus sequence was aligned back to the reference genome, then the best 20 BLASTN^69^ hits were collected, extended by 2 kb and aligned to one another using MAFFT (v6^70^). The alignments were manually curated applying a majority-rule for consensus sequence generation and the superfamily of each repeat assessed following Wicker et al.^71^. We then masked the new consensus sequences in CENSOR (http://www.girinst.org/censor/index.php) and named them according to homology to known repeats present in Repbase^72^. Repeats with high sequence similarity to known repeats were given the name of the known repeat + suffix “_corCor”; repeats with partial homology were named with the suffix “-L_corCor” where “L” stands for “like”^34^. Repeats with no homology to other known repeats were considered as new families and named with the prefix “corCor” followed by the name of their superfamilies. Using this fully curated repeat library we performed a RepeatMasker^73^ search on all sequences reported for insertion and deletion variants. In case of multiple different matches per variant or individual, we took the match with the highest overlap with the query sequence to yield a single match for each variant. We also performed a RepeatMasker search with the curated library to estimate repeat density per 1 Mb window in the hooded crow reference assembly. The curated repeat library is available as Supplementary Data 3 and the detailed annotation of curated repeats in Supplementary Table 7.

### Population genetic analysis of structural variants

To investigate population structure, we performed principal component analyses (PCA) with both the long-read and short-read variant sets using the R packages SNPrelate (v1.4.2) and gdsfmt (v1.6.2)^74^. We further calculated the folded allele frequency spectrum using minor allele frequencies of variants for all populations and clades. To estimate genetic differentiation of structural variations, we calculated *F*_ST_, using the Weir and Cockerham estimator for *F*_ST_^75^, for each variant using vcftools^76^. Variants with an *F*_ST_ exceeding the 99th percentile were considered as outliers flagging candidate genes with relevance for population divergence.

### Optical mapping-based SV detection

The assembled optical maps were used to identify SV compared to the provided reference, which is part of the 1.3.1 Bionano Solve pipeline 3.3.1 (pipeline version 7841). SV calling was based on the alignment between an individual assembled consensus cmap and the in silico generated map of the reference using a multiple local alignment algorithm and detecting SV signatures. The detection algorithm identifies insertions, deletions, translocation breakpoints, inversion breakpoints, and duplications. The results are a file generated in the BioNano-specific format smap in which the SVs are classified as homozygous or heterozygous. This resulting smap file was converted to vcf format (version 4.2) for further downstream processing (Supplementary Data 4).

### Analyses of SV in the vicinity of the NDP gene

The LTR retrotransposon insertion identified upstream of the *NDP* gene on chromosome 1—an ERVK element belonging to the subfamily corCorLTRK1b—has initially been called as a deletion relative to the reference (hooded crow) assembly. To estimate its age, we assumed that the two long terminal repeats of the full-length LTR retrotransposon were identical at the time of insertion^77^. Thus, we quantified the number of substitutions and 1-bp indels between the left and right LTR of the insertion at position 112,179,329 on chromosome 1 of the hooded crow reference. The LTRs showed five differences which we then divided by the length of the LTR (296 bp) and by twice the neutral substitution rate per site and million years (0.0158, Vijay et al.^23^). Assuming that all differences between the left and right LTR of this insertion are fixed, this estimate yields an upper bound of the insertion age. However, overlap with SNPs segregating in the hooded crow population suggests that all five differences were not fixed and the insertion could thus be considerably younger.

To investigate a potential link between the LTR insertion and differences in plumage coloration, we re-analyzed gene expression data from ten black-and-gray hooded crows (*C. (c.) cornix*) and eight all-black carrion (*C. (c.) corone*) crows raised under common garden conditions^26^. Expression was measured for messenger RNA derived from feather buds at the torso, where carrion crows have black feathers, and hooded crows are gray. We inferred the insertion genotype for each individual using short-read sequencing data via visual inspection of alignments to the hooded crow reference. We then fitted a linear model with normalized *NDP* expression (for head and body skin tissue) data as the dependent variable and *NDP* indel genotype as the predictor. We decomposed the effect of the insertion genotype into an additive component (the number of non-inserted minor allele copies – 0, 1, or 2 – as a covariate) and a dominance component (homozygous = 0, heterozygous = 1).

To identify highly conserved regions indicative of regulatory elements in the vicinity of the LTR retrotransposon insertion, we aligned 5 kb of the flanking sequence on either site of the insertion in the hooded crow genome to the chicken reference assembly (galGal6) on the UCSC GenomeBrowser (https://genome.ucsc.edu). Visual inspection of conservation scores and human chain alignment tracks revealed a highly conserved region in the 3′ flanking sequence (Supplementary Fig. 9).

To further establish a link between the LTR retrotransposon insertion and phenotypic differences, we made use of a hybrid admixture data set from the European hybrid zone^25^. We designed three sets of PCR primers to genotype the insertion for 120 phenotyped individuals from the European hybrid zone of all-black *C. (c.) corone* and black-and-gray *C. (c.) cornix* crows. For absence of the insertion, a pair of primers located in the sequence flanking the insertion was used (A_F_3 “AGTAACTGTCCTCTGTAGTGCAGG” and A_R_3 “CCTGGGTAAGATCACAGTGTTGC”) resulting in a 197 bp fragment. For presence of the insertion, two pairs of primers with one in the flanking and one in either left or right LTR region of the insertion (P_L_F_1 “TCCTCTGTAGTGCAGGACTGG” and P_L_R_2 “CACCCATGGTTTCCCTCACA”, as well as P_R_F_1 “GGATCGGGGATCGTTCTGCT” and P_R_R_1 “CACAGCCCCAGAAGATGTGC”) was used, resulting in fragments of 659 and 564 bp, respectively. A representative gel picture used for genotyping can be found in the Supplementary Fig. 10. Phenotypic data was taken from Knief et al.^25^ who summarized 11 plumage color measures on the dorsal and ventral body into a principal component (PC1), explaining 78% of the phenotypic variation. We then tested whether the interaction between chromosome 18 and the insertion genotype explained more variation in plumage color than the interaction between chromosome 18 and the most significant SNP near the *NDP* gene^25^. We fitted two linear regression models on the same subset of the data that contained no missing genotypes (*N* = 120 individuals, ref. ^78^). In both models, we used color PC1 as our dependent variable. In the first model, we fitted the interaction between chromosome 18 and the insertion genotype, and in the second model the interaction between chromosome 18 and the SNP genotype as our independent variables. Both variables were coded as 0, 1, 2 copies of the derived allele and fitted as factors. We selected the model with the better fit to the data by estimating the AICc and BIC and deemed a ΔAICc ≥ 2 as significant.

### Reporting summary

Further information on research design is available in the Nature Research Reporting Summary linked to this article.

## Supplementary information

Supplementary Information

Peer Review

Reporting Summary

Description of Additional Supplementary Files

Supplementary Dataset 1-5

## Data Availability

All sequencing and optical mapping data have been deposited in the National Center for Biotechnology Information (NCBI) database under project number PRJNA634260. The newly generated reference assembly for the jackdaw individual is available under the GenBank accession JABDSK000000000. The hooded crow genome assembly has been deposited in the Dryad repository [10.5061/dryad.ns1rn8ppj]. Source data are provided with this paper.

## Source data

Source Data file

## Code availability

All custom scripts and pipelines developed in for this project are available as Supplementary Data 5 and at [https://github.com/EvoBioWolf/Corvid_SVpopgen]. Source data are provided with this paper.
