# Author Correction: Discovery and population genomics of structural variation in a songbird genus

**DOI:** 10.1038/s41467-021-23640-9

**Published:** 2021-05-20

**Authors:** Matthias H. Weissensteiner, Ignas Bunikis, Ana Catalán, Kees-Jan Francoijs, Ulrich Knief, Wieland Heim, Valentina Peona, Saurabh D. Pophaly, Fritz J. Sedlazeck, Alexander Suh, Vera M. Warmuth, Jochen B. W. Wolf

**Affiliations:** 1 Department of Evolutionary Biology and Science for Life Laboratory, Uppsala University, 752 36 Uppsala, Sweden; 2 Division of Evolutionary Biology, Faculty of Biology, LMU Munich, Grosshaderner Str. 2, 82152 Planegg-Martinsried, Germany; 3 Uppsala Genome Center, Science for Life Laboratory, Department of Immunology, Genetics and Pathology, Uppsala University, BMC, Box 815752 37, Uppsala, Sweden; 4 BioNanoGenomics, San Diego, CA 92121 USA; 5 Institute of Landscsape Ecology, University of Münster, Heisenbergstrasse 2, 48149 Münster, Germany; 6 Human Genome Sequencing Center at Baylor College of Medicine, 1 Baylor Plaza, Houston, TX 77030 USA; 7 Present Address: Department of Biology, Pennsylvania State University, 310 Wartik Lab, PA 16802 USA; 8 Present Address: Department of Organismal Biology – Systematic Biology, Uppsala University 752 36 Uppsala, Sweden; 9 Present Address: Max Planck Institute for Plant Breeding Research, Carl-von-Linné-Weg 10, 50829 Cologne, Germany; 10 Present Address: School of Biological Sciences, University of East Anglia, Norwich Research Park, Norwich, NR4 7TU UK

**Keywords:** Evolutionary genetics, Population genetics, Structural variation, Comparative genomics

Correction to: *Nature Communications* 10.1038/s41467-020-17195-4, published online 07 July 2020.

The original version of this Article contained an error in Fig. 1b, in which the labels ‘SNP count per 1 Mb window’ and ‘SV count per 1 Mb window’ are switched.

This has been corrected in both the PDF and HTML versions of the Article.

