# Supplementary material for: Discovery and population genomics of structural variation in a songbird genus
Source: Nat Commun. 2020 Jul 7;11:3403. doi: 10.1038/s41467-020-17195-4 (PMC7341801; doi:10.1038/s41467-020-17195-4)
Supplement: Supplementary file 1 — Supplementary Information [file 41467_2020_17195_MOESM1_ESM.pdf]

Supplementary Information for

Discovery and population genomics of structural variation in a songbird genus

Weissensteiner et al. 2020

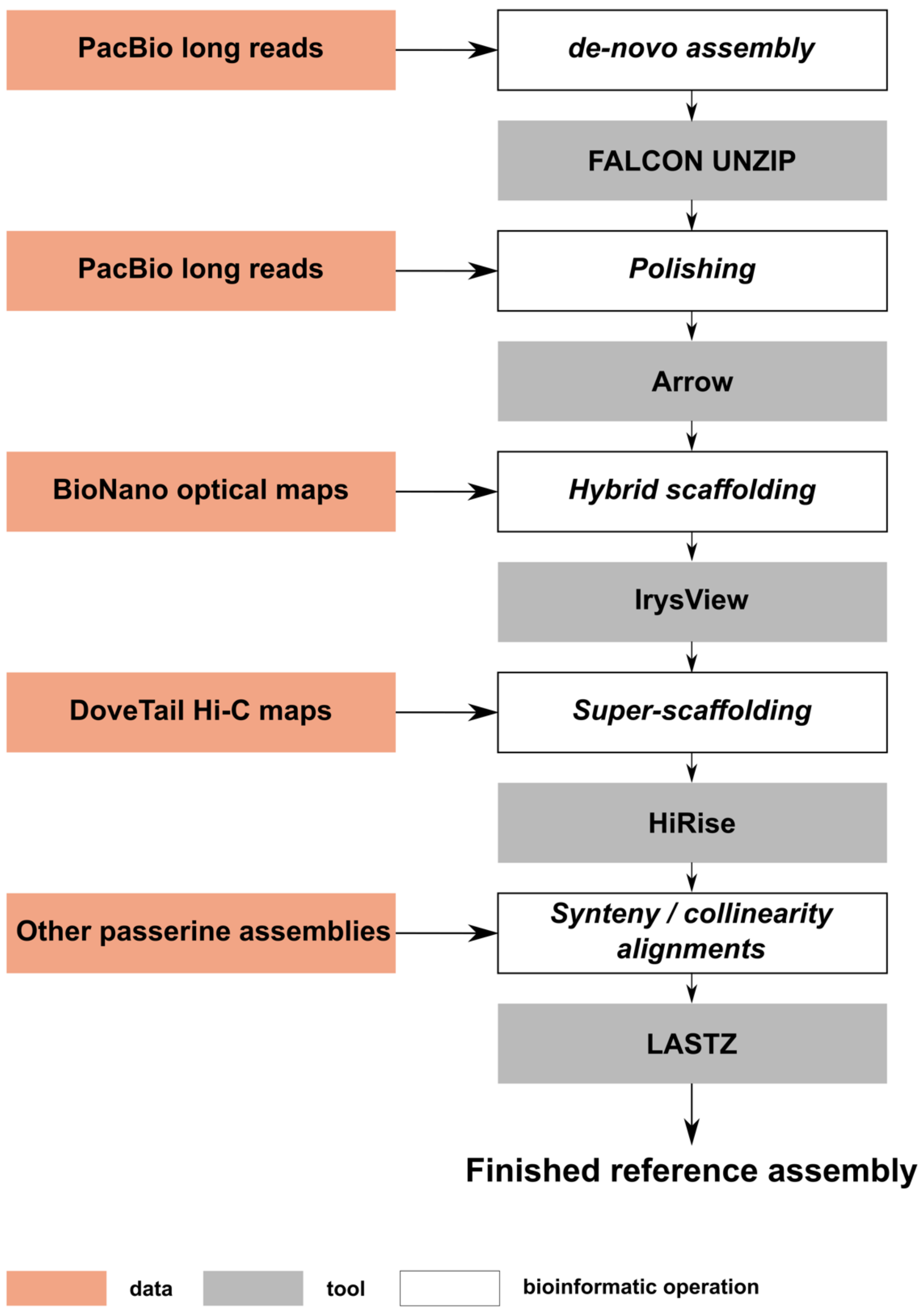

Supplementary Figure 1. Workflow for building the hooded crow reference assembly.

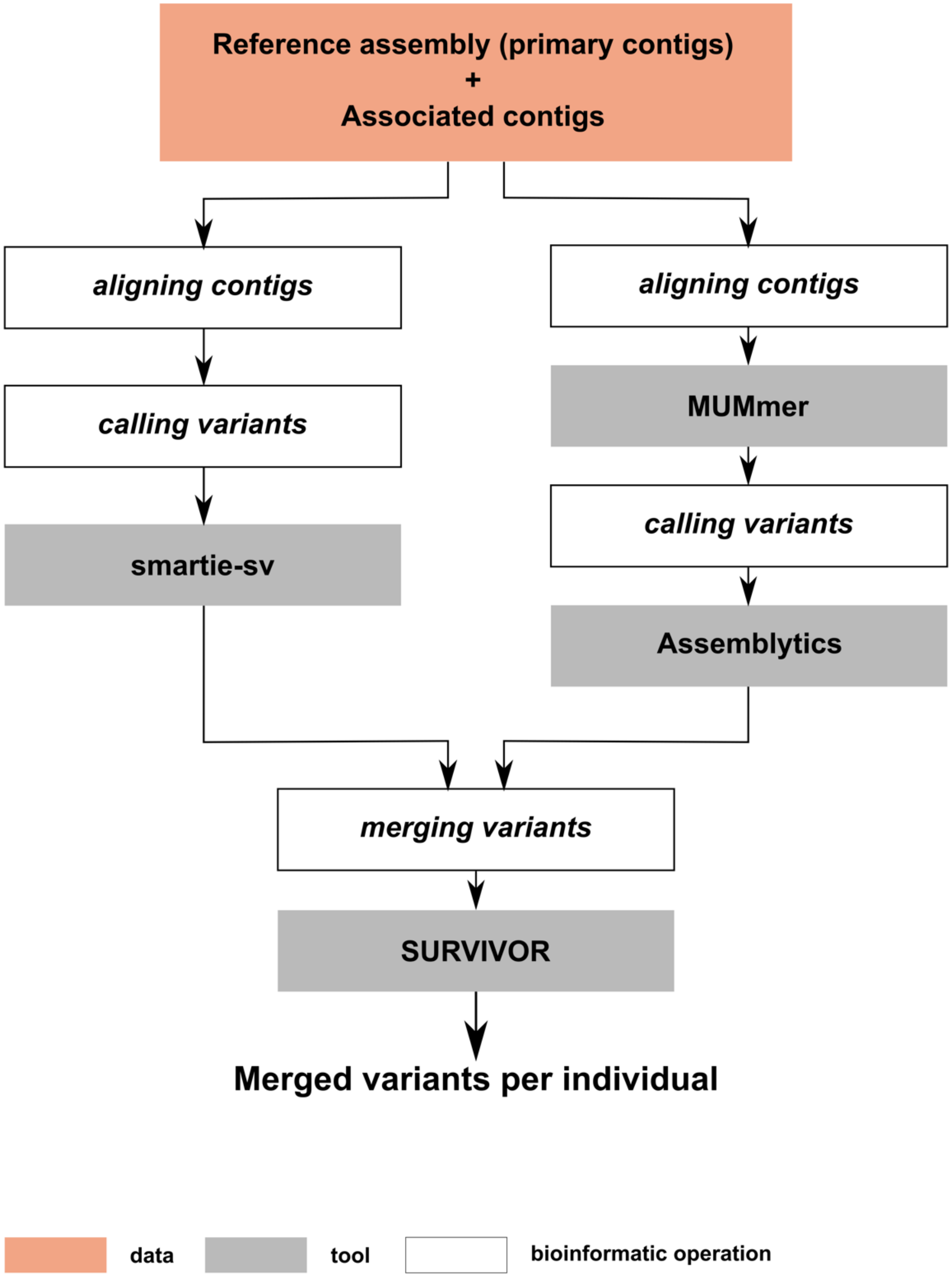

Supplementary Figure 2. Workflow for assembly-based SV detection using both haplotypes of a single-individual genome assembly.

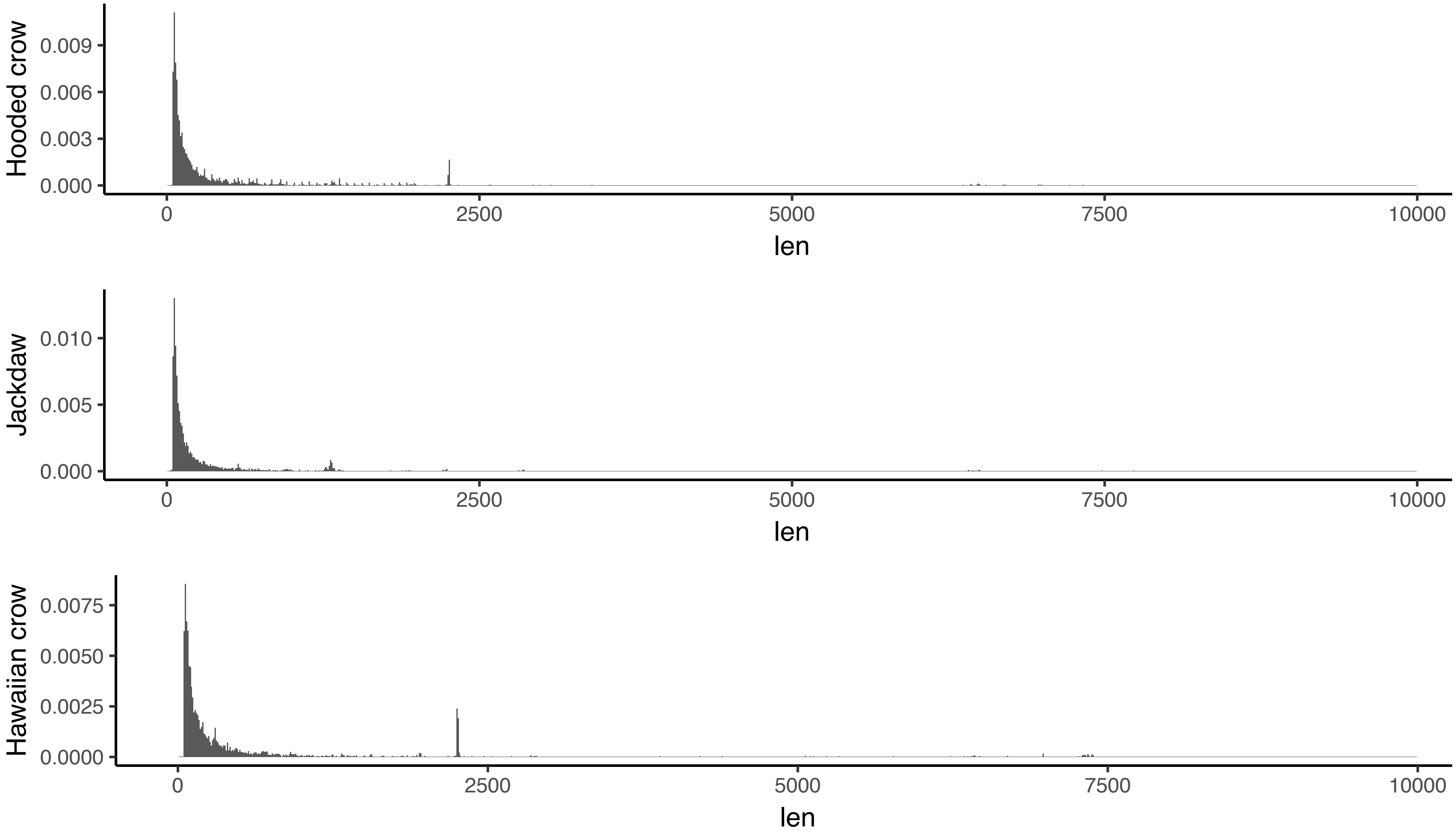

Supplementary Figure 3. Density histogram of assembly-based SV lengths < 10kb. Hooded crow and Hawaiian crow exhibit similar peaks in SV length at ~2.3 kb, whereas jackdaw shows a distinct peak at ~1.2 kb.

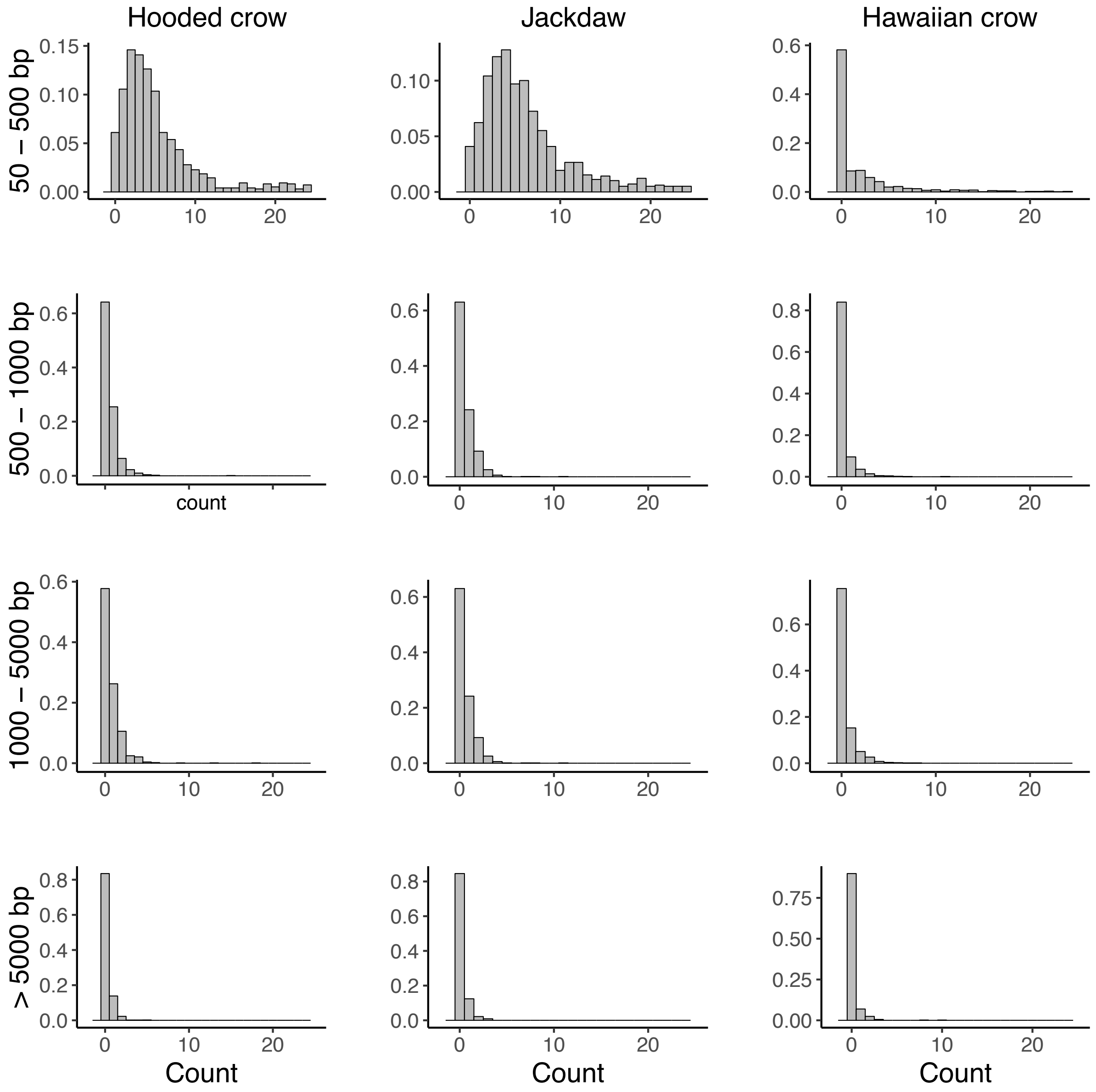

Supplementary Figure 4. Genome-wide SV counts in 1-Mb genomic windows for four different size categories of structural mutations (50 – 500 bp, 500 – 1000 bp, 1000 – 5000 bp and > 5000 bp).

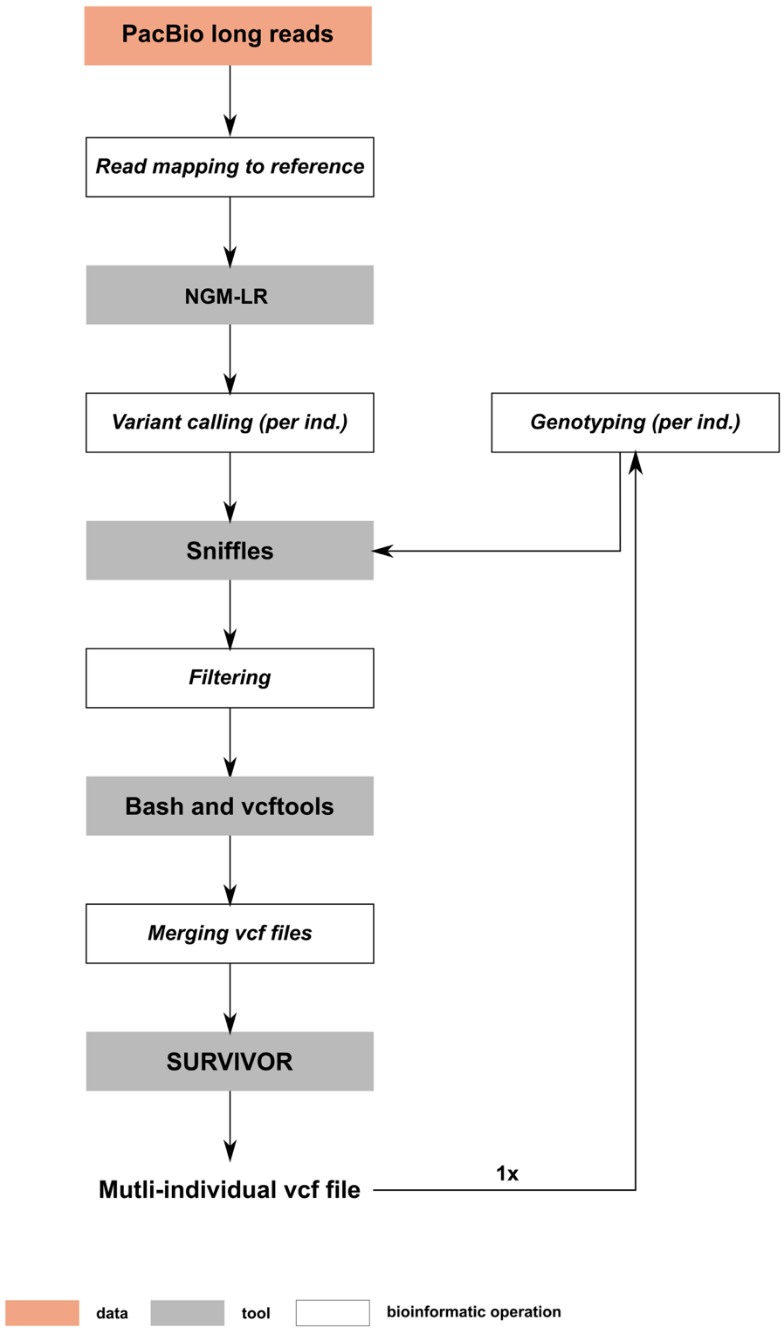

Supplementary Figure 5. Workflow for long-read sequencing-based SV detection combining multiple individuals.

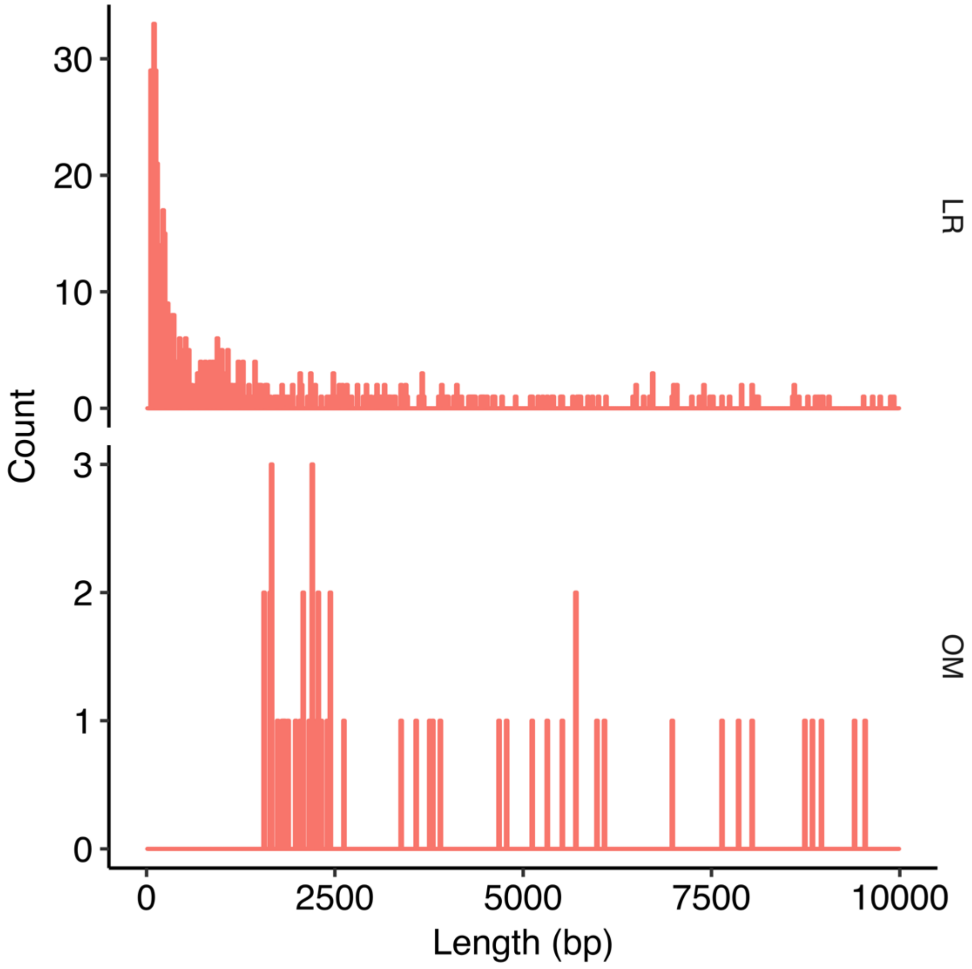

Supplementary Figure 6.

Length distribution of inversions shorter than 10 kb identified with LR (top panel) and OM (bottom panel). Note the different scale on the y-axis.

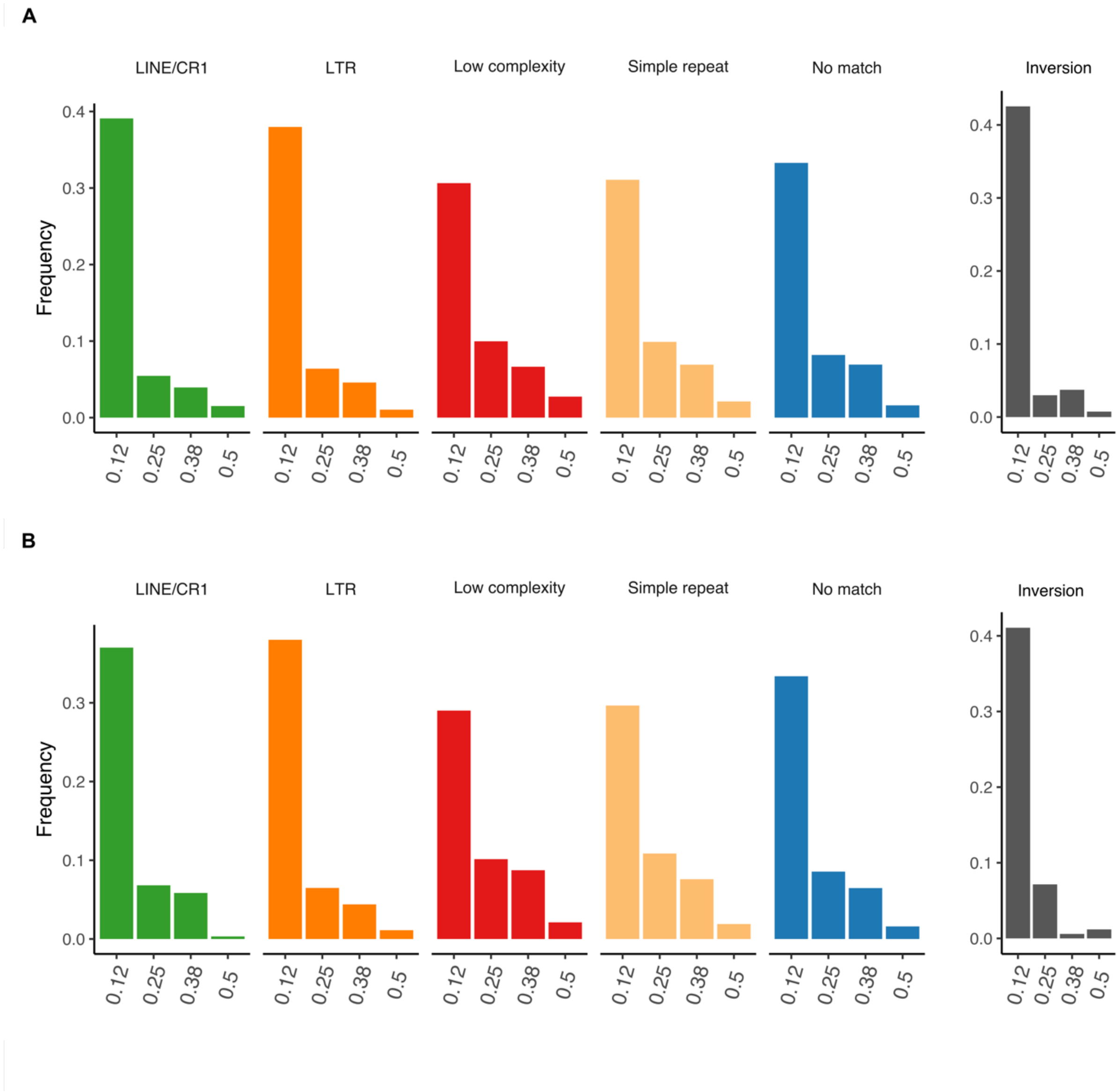

Supplementary Figure 7.
Folded allele frequency spectra LR-based SV of A) the hooded crow population and B) the German carrion crow population.

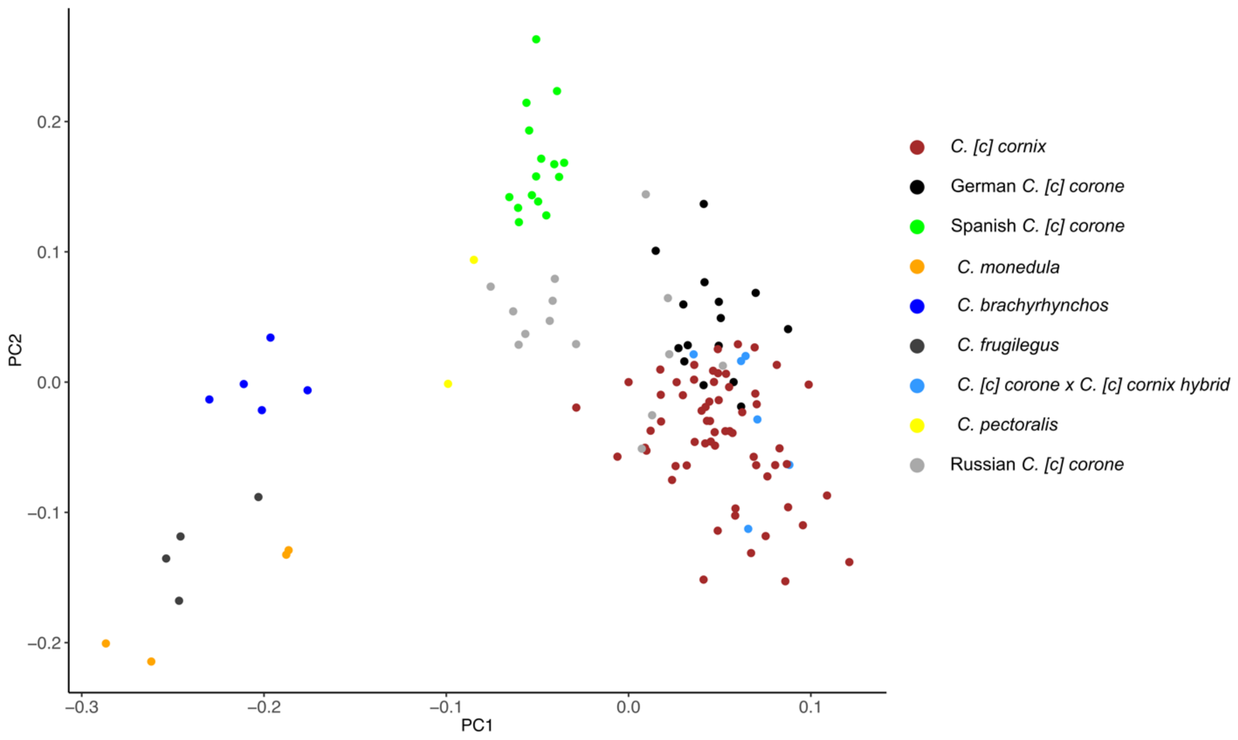

Supplementary Figure 8.
Principal component analysis of SV based on SR data.

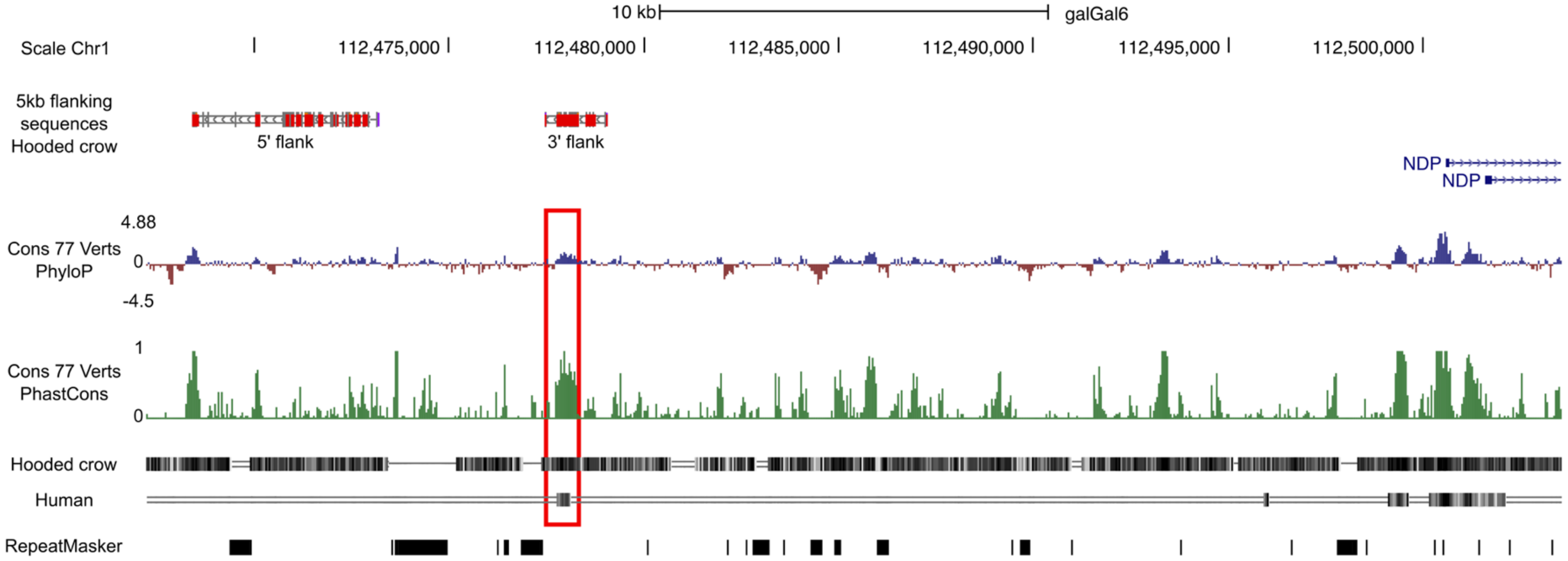

Supplementary Figure 9. The LTR retrotransposon upstream of the *NDP* gene is close to a highly conserved non-coding element. Shown is the upstream region of the *NDP* gene in the chicken (galGal6) genome assembly, retrieved from UCSC GenomeBrowser (**http://genome.ucsc.edu**). Tracks in red denote BLAT hits of 5-kb flanks of the LTR retrotransposon insertion identified in the hooded crow aligned to the chicken genome, i.e., the orthologous locus of the LTR insertion is located between these. Interestingly, the 3' flanking sequence harbors a highly conserved region (red box), illustrated by conservation score peaks (PhyloP 77 vertebrates, blue / red track, PhastCons 77 vertebrates, green track) and a partial alignment of chicken to hooded crow and even human (second and third track from the bottom). We hypothesize that the proximity of the LTR insertion to this conserved non-coding element (2.8 kb distance) has a down-regulatory effect on *NDP* expression.

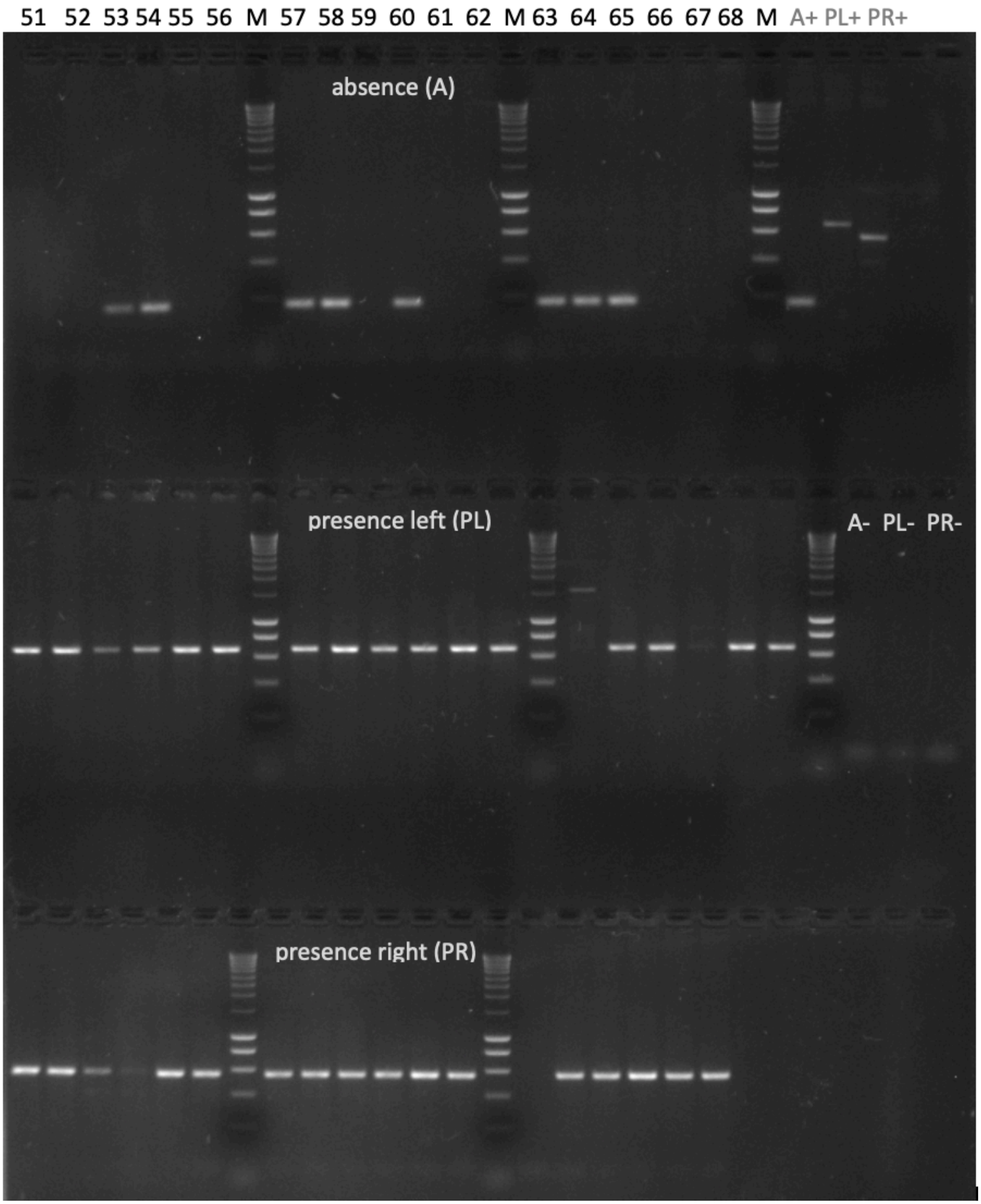

Supplementary Figure 10.

Representative gel picture of the LTR retrotransposon insertion genotyping in the vicinity of the *NDP* gene on chromosome 1. Numbered columns show focal individual genotyped for three different PCR fragments.

Supplementary Table 1.

Assembly statistics of genome assemblies generated for this study.

Assembly	Number of scaffolds	Total length	Longest scaffold	Mean scaffold length	Median scaffold length	Scaffold N50	Number of contigs	Total length	Longest contig	Contig N50	% complete BUSCOs vertebrate	% complete BUSCOs aves
Hooded crow - Super-scaffolded primary assembly	48	1037. 32	154.82	21.61	6.88	74.11	810	1033.98	33.50	7.83	94,39	88,67
Hooded crow - FALCON UNZIP associated assembly	-	-	-	-	-	-	7645	965.95	1.80	0.22	83,49	78,90
Jackdaw - Super-scaffolded primary assembly	136	1042. 43	58.30	7.66	3.436	16.38	1607	1036.86	52.68	12.14	93,70	93,70
Jackdaw - FALCON UNZIP associated assembly	-	-	-	-	-	-	6349	1009.69	3.10	0.42	84,30	84,30
Hawaiian crow - FALCON UNZIP primary assembly	-	-	-	-	-	-	670	1064.97	31.53	7.73	95,05	95,05
Hawaiian crow - FALCON UNZIP associated assembly	-	-	-	-	-	-	2082	432.63	9.52	0.45	42,65	39,47

Supplementary Table 2.

Long-read sequencing data. Summary of sequencing data generated with PacBio SMRT-sequencing technology.

Run_ID	Number of reads	Amount sequenced [Gbp]	Longest read [kbp]	Mean read length [kbp]	Median read length [kbp]
D_Ko_C29__pb_247_001	1319078	10,80	78,574	8,187	7,726
E_Vi_C58__pb_375_006	1426456	13,54	53,867	9,493	8,682
S_To_J14__pb_382_001	1646343	13,18	61,411	8,005	7,252
S_Up_H24__pb_382_002	1543348	13,59	67,679	8,805	7,837
S_Up_H29__pb_382_003	1378152	13,38	70,966	9,709	8,756
USA_Wa_B02__pb_383_005	2282703	18,93	53,388	8,294	7,753
D_Ko_C04__pb_375_004	1388443	12,95	69,752	9,328	8,528
S_Up_H47__pb_382_004	1561579	14,44	67,569	9,246	8,45
S_Up_H29__pb_410_002	1472021	12,19	47,971	8,284	7,298
E_Vi_C103__pb_410_003	1739876	12,84	49,815	7,38	6,895
E_Vi_C57__pb_410_004	1668218	12,98	51,044	7,778	6,812
Pl_Wa_H24__pb_410_006	1407040	13,19	51,352	9,371	8,795
Pl_Wa_H22__pb_410_005	1400972	12,92	50,659	9,222	8,814
D_Ko_C13__pb_410_007	1449483	13,49	50,611	9,307	9,027
S_To_J15__pb_410_010	1665168	12,16	50,03	7,301	6,787
USA_Wa_B02__pb_410_009	1449719	12,55	47,961	8,657	8,225
USA_Wa_B01__pb_410_008	1461798	12,60	50,766	8,618	8,3
S_Up_H24__pb_410_001	2155243	13,52	46,222	6,272	5,18
S_Up_H32__pb_210_001	1599921	12,57	52,426	7,858	6,945
S_Up_H32__pb_260_002	858882	5,71	45,288	6,644	5,916
S_Up_H32__pb_260_003	2307285	15,06	47,199	6,527	5,744
S_Up_H32__pb_260_001	4655884	29,74	50,116	6,388	5,647
S_Up_J01__pb_298_001	9614265	83,33	53,658	8,667	7,923
S_Up_H03__ps_024_006	2526698	17,22	87,77	6,814	5,6
D_Ko_C36__ps_024_005	3684211	23,70	88,122	6,433	5,583
D_Ra_C16__ps_024_002	2779764	19,33	98,694	6,955	6,209

D_Ko_C15__ps_024_008	2851263	16,85	144,589	5,91	4,965
S_To_J10__ps_024_010	4590885	28,05	99,725	6,109	4,886
D_Ko_C31__ps_024_009	3888521	27,36	113,93	7,036	5,456
S_Up_H37__ps_024_007	3248427	21,08	94,114	6,49	6,004
S_To_J13__ps_024_019	4971825	32,39	104,698	6,513	5,659
S_Up_H59__ps_024_022	4447600	31,70	106,717	7,127	5,921
D_Ra_C05__ps_024_021	4463592	30,86	116,395	6,913	5,655
E_Vi_C98__ps_038_001	2403048	16,58	77,51	6,898	5,415
E_Vi_C101__ps_038_004	3123591	19,89	97,839	6,368	5,098
E_Vi_C100__ps_038_002	4011123	22,76	80,83	5,675	5,339
RUS_Mp_D06__ps_038_007	2106565	21,14	92,069	10,034	7,805
RUS_Mp_D08__ps_038_006	4114486	21,48	85,377	5,219	5,676
RUS_Mp_D04__ps_038_005	3524965	18,73	73,561	5,314	5,104
Total	104188441	754,80	72,57087179	7,5347	6,719

Supplementary Table 3.

Frequencies of individual repeat types in retained insertions and deletions.

Repeat ID	Frequency	Repeat class	Length
TguERVK7-La_corCor	1356	LTR	670
TguLTRL2-Lc_corCor	1138	LTR	1315
corCor_ERV_chimera-head_corCorLTRK1b-L	893	LTR	2072
A-rich	785	Low_complexity	NA
TguERVL2-La_corCor	768	LTR	564
G-rich	758	Low_complexity	NA
TguERVL2-Le_corCor	751	LTR	906
(T)n	724	Simple_repeat	NA
GA-rich	670	Low_complexity	NA
corCorLTRK1b_LTR	509	LTR	294
(A)n	472	Simple_repat	NA
corCorLTRK15	432	LTR	966
corCorLTRK12	414	LTR	481
corCorLTRK13	353	LTR	575
TguLTRL1-La_corCor	343	LTR	640
lycPyrLTRL11	328	LTR	1238
(C)n	316	Simple_repeat	NA
(TA)n	280	Simple_repeat	NA
(G)n	237	Simple_repeat	NA
corCorLTRK17_LTR	221	LTR	375
(AT)n	212	Simple_repeat	NA
TguLTRL2-La_corCor	198	LTR	1303
corCorLTR1	184	LTR	461
(TTCC)n	180	Simple_repeat	NA
(CCTT)n	178	Simple_repeat	NA
corCorLTRK1a_I	177	LTR	1736
(GGAA)n	170	Simple_repeat	NA
TguLTRL4a	169	LTR	1154
corCorLTRK1d_LTR	169	LTR	299
(GGGAA)n	151	Simple_repeat	NA
(TTTC)n	124	Simple_repeat	NA

Supplementary Table 4.

F_{ST} outliers in the all-black German carrion crow and black-and-gray hooded crow comparison based on LR variants.

Chromosome	Position	Type	Length	FST	Gene downstream	Distance	Gene upstream	Distance
Super-Scaffold_9_Super-Scaffold_99__chr18	10083548	DEL	86	0.812405	SLC16A6	-26679	ARSG	301
Sc8eucV_19_HRSCAF_154__chr3	78483754	DEL	1563	0.666196	GABRR2	-9425	UBE2J1	850
Sc8eucV_16_HRSCAF_138__chr1	112179329	DEL	2255	0.616612	NDP	-26270	EFHC2	37138
Sc8eucV_12_HRSCAF_113__chr4A	10168413	DEL	659	0.556894	GRIA3	-204950	MCTS1	304532
Sc8eucV_10_HRSCAF_100__chr15	9876103	INS	920	0.542418	SLC5A1	-25446	YWHAH	2969
Sc8eucV_6_HRSCAF_52__chr8	81990	DEL	1338	0.542418	SLC44A5	0	SLC44A5	0
Super-Scaffold_9_Super-Scaffold_99__chr18	9080135	INS	56	0.542418	LOC104696015	-966	BPTF	15959
Sc8eucV_21_HRSCAF_161__chr4	30401968	DEL	819	0.528082	GIMD1	-91886	DKK2	43851
Super-Scaffold_9_Super-Scaffold_99__chr18	10160422	DEL	608	0.515687	FAM20A	-9970	LOC104693358	21836
Sc8eucV_28_HRSCAF_204__chrZ	42855849	DEL	6523	0.498962	IFT74	-436763	CAAP1	402328
Sc8eucV_17_HRSCAF_144__chr12	14067812	INS	134	0.498818	PSMD6	-25685	PRICKLE2	3640
Sc8eucV_41_HRSCAF_239__chr2	11673240	DEL	115	0.45518	PITRM1	-102277	PFKP	132560
Sc8eucV_21_HRSCAF_161__chr4	10686903	DEL	1481	0.453426	MAML3	-18719	MGST2	32272
Sc8eucV_19_HRSCAF_154__chr3	93939333	INS	58	0.439911	NA	NA	NA	NA
Sc8eucV_28_HRSCAF_204__chrZ	34154260	DEL	567	0.437665	NA	NA	NA	NA
Sc8eucV_16_HRSCAF_138__chr1	435789	DEL	100	0.430649	SLAMF9	-79446	LOC104698121	31026
Sc8eucV_20_HRSCAF_160__chr1A	22139103	DEL	74	0.430649	PTPRZ1	0	PTPRZ1	0
Super-Scaffold_9_Super-Scaffold_99__chr18	6551090	INS	92	0.430649	CA10	-972	UTP18	282159
Super-Scaffold_9_Super-Scaffold_99__chr18	9954827	INS	64	0.427794	AXIN2	-95524	RGS9	14188
Sc8eucV_10_HRSCAF_100__chr15	1130347	INS	119	0.425766	GLT1D1	-31874	SLC15A4	8750
Sc8eucV_14_HRSCAF_135__chr10	18758127	DEL	70	0.425766	MPI	-16805	SCAMP2	4290

Sc8eucV_20_HRSCAF_160__chr1A	40984218	INS	295	0.425766	SLC6A15	-13521	TSPAN19	43707
Sc8eucV_19_HRSCAF_154__chr3	64545414	DEL	3696	0.42464	MAN1A1	-326161	TBC1D32	296556
Sc8eucV_19_HRSCAF_154__chr3	43076707	DEL	569	0.417853	DISC1	0	DISC1	0
Sc8eucV_22_HRSCAF_163__chr9	24812560	DEL	289	0.414455	PCCB	-20580	PPP2R3A	13552
Sc8eucV_12_HRSCAF_113__chr4A	1505992	DEL	114	0.408223	LOC104691275	-372	EDA2R	21394
Sc8eucV_5_HRSCAF_38__chr7	1789800	DEL	221	0.408223	MMADHC	-26020	LYPD6	40702
Sc8eucV_12_HRSCAF_113__chr4A	16945599	DEL	129	0.407186	LOC104691732	-34917	PIH1D3	21123
Sc8eucV_12_HRSCAF_113__chr4A	8868927	DEL	215	0.407186	NRK	0	NRK	0
Sc8eucV_41_HRSCAF_239__chr2	31827203	DEL	162	0.388603	ELMO1	0	ELMO1	0
Sc8eucV_19_HRSCAF_154__chr3	56433950	DEL	597	0.388519	ESR1	0	ESR1	0
Sc8eucV_2_HRSCAF_5__chr5	37341942	DEL	400	0.387775	LOC104698601	0	LOC104698601	0
Sc8eucV_2_HRSCAF_5__chr5	47984643	DEL	143	0.387279	TH	-15362	LOC104687704	119783
Sc8eucV_11_HRSCAF_110__chr11	2373236	INS	131	0.381214	GSE1	-22151	GINS2	40332
Sc8eucV_6_HRSCAF_52__chr8	478781	DEL	93	0.381214	NEGR1	-61593	ERICH3	227018
Super-Scaffold_8_Super-Scaffold_98__chr28	1864815	INS	149	0.381214	ADAMTS10	-72880	ZAP70	35464
Sc8eucV_21_HRSCAF_161__chr4	41662680	DEL	7777	0.379839	SORBS2	-384955	MTNR1A	95511
Sc8eucV_41_HRSCAF_239__chr2	47695276	INS	146	0.379596	IL6	-10879	LOC104689112	21839
Sc8eucV_16_HRSCAF_138__chr1	2398759	INS	68	0.37461	LOC104689368	-75896	LOC104689433	39839
Sc8eucV_2_HRSCAF_5__chr5	38174309	DEL	135	0.372993	RTF1	0	RTF1	0
Sc8eucV_41_HRSCAF_239__chr2	84746331	INS	80	0.372993	COBL	-357811	LOC104685918	289065
Sc8eucV_6_HRSCAF_52__chr8	1129691	DEL	79	0.372993	LOC104695493	-2511	WLS	2023
Sc8eucV_2_HRSCAF_5__chr5	15012660	INS	75	0.371391	PPP4R4	0	PPP4R4	0
Sc8eucV_11_HRSCAF_110__chr11	14615394	INS	59	0.366293	LOC104687436	-3416	POP4	358
Sc8eucV_19_HRSCAF_154__chr3	54586748	DEL	61	0.365118	NOX3	-113289	CLDN20	189881
Super-Scaffold_8_Super-Scaffold_98__chr28	3607722	DEL	90	0.365118	LOC104697075	0	LOC104697075	0
Super-Scaffold_9_Super-Scaffold_99__chr18	10166476	INS	151	0.365118	FAM20A	-16024	LOC104696001	23298
Super-Scaffold_9_Super-Scaffold_99__chr18	10244723	DEL	91	0.365118	LOC104696000	-7678	ABCA5	1943
Super-Scaffold_143__chr26	6291854	INS	282	0.357143	PPFIA4	0	PPFIA4	0

Sc8eucV_16_HRSCAF_138__chr1	8432823	INS	916	0.351229	ROBO1	-235266	LOC104689417	266974
Sc8eucV_20_HRSCAF_160__chr1A	27000693	DEL	2184	0.351229	IMMP2L	0	IMMP2L	0
Sc8eucV_21_HRSCAF_161__chr4	11319706	INS	52	0.351229	RNF150	-822	LOC104697268	95611
Sc8eucV_2_HRSCAF_5__chr5	564728	INS	100	0.351229	FAM179B	-211063	KLHL28	169095
Sc8eucV_41_HRSCAF_239__chr2	9079818	INS	460	0.351229	LOC104686440	0	LOC104686440	0
Sc8eucV_7_HRSCAF_58__chr13	1843425	DEL	448	0.351229	LOC104689637	-6048	SLC26A2	8279
Sc8eucV_16_HRSCAF_138__chr1	60691922	DEL	912	0.34902	LOC104693136	-557782	DIAPH3	298389
Sc8eucV_22_HRSCAF_163__chr9	24517238	DEL	2864	0.34902	MAP3K13	0	MAP3K13	0
Super-Scaffold_9_Super-Scaffold_99__chr18	5715351	DEL	51	0.34902	ACSF2	0	ACSF2	0
Sc8eucV_11_HRSCAF_110__chr11	2409601	DEL	149	0.345105	GSE1	0	GSE1	0
Sc8eucV_16_HRSCAF_138__chr1	47207238	INS	74	0.340705	WASF3	-4322	CDK8	45576
Sc8eucV_6_HRSCAF_52__chr8	23785773	DEL	300	0.340705	LOC104695422	-48461	CDC73	9165
Sc8eucV_41_HRSCAF_239__chr2	74069849	INS	384	0.340533	CDH9	0	CDH9	0
Sc8eucV_12_HRSCAF_113__chr4A	13659348	DEL	54	0.339498	LOC104691679	0	LOC104691679	0
Sc8eucV_5_HRSCAF_38__chr7	982205	INS	92	0.337316	DRC1	-14449	GALNT13	55917
Sc8eucV_41_HRSCAF_239__chr2	101128609	INS	324	0.336903	NA	NA	NA	NA
Sc8eucV_28_HRSCAF_204__chrZ	73797802	INS	73	0.330544	EDIL3	-406650	LOC104695841	282404
Sc8eucV_20_HRSCAF_160__chr1A	67154259	INS	51	0.329462	PRR5	0	PRR5	0
Sc8eucV_28_HRSCAF_204__chrZ	13197166	INS	573	0.329462	RAI14	0	RAI14	0
Sc8eucV_2_HRSCAF_5__chr5	5602372	DEL	830	0.329462	PRKCH	-64676	SLC38A6	52965
Sc8eucV_6_HRSCAF_52__chr8	10448114	DEL	60	0.329462	IPO13	-9585	ST3GAL3	4992
Super-Scaffold_9_Super-Scaffold_99__chr18	7026905	INS	97	0.329462	LOC104694274	-17172	LOC104694356	204630
Sc8eucV_2_HRSCAF_5__chr5	28446815	DEL	53	0.326572	LOC104684460	-210386	LOC104684431	41038
Super-Scaffold_9_Super-Scaffold_99__chr18	2181334	DEL	184	0.326572	LOC104695636	-20399	LOC104695682	1877
Sc8eucV_20_HRSCAF_160__chr1A	29292175	INS	55	0.321524	ADAMTS20	0	ADAMTS20	0
Sc8eucV_19_HRSCAF_154__chr3	77188793	DEL	6950	0.321188	MAP3K7	-695846	EPHA7	397902
Sc8eucV_2_HRSCAF_5__chr5	6552052	INS	108	0.321188	WDR89	0	WDR89	0
Sc8eucV_41_HRSCAF_239__chr2	26678831	DEL	1164	0.321188	MRPL32	-103684	LOC104688910	82671

Sc8eucV_19_HRSCAF_154__chr3	114426297	DEL	89	0.319956	DTNB	0	DTNB	0
Sc8eucV_10_HRSCAF_100__chr15	11369903	DEL	584	0.315981	LOC104694978	-111902	AIFM3	200211
Super-Scaffold_503__chr27	4947702	DEL	192	0.313759	LOC104685769	0	LOC104685769	0
Sc8eucV_19_HRSCAF_154__chr3	66564293	INS	160	0.312588	NT5DC1	-214535	FRK	151867
Sc8eucV_1_HRSCAF_4__chr6	16956472	INS	79	0.312588	KIF20B	0	KIF20B	0
Sc8eucV_21_HRSCAF_161__chr4	55571864	INS	155	0.312588	PPARGC1A	-48196	LOC104690704	53198
Sc8eucV_20_HRSCAF_160__chr1A	10564911	DEL	621	0.311616	GNAI1	-144700	GNAT3	241037
Sc8eucV_6_HRSCAF_52__chr8	29238870	DEL	1209	0.311616	CCDC180	-528731	LOC104693596	37188
Sc8eucV_5_HRSCAF_38__chr7	33426000	DEL	7076	0.307692	ZNF804A	-178769	LOC104698251	112821
Sc8eucV_13_HRSCAF_122__chr20	1264893	DEL	190	0.306888	LOC104698034	-2824	LOC104698050	42840
Sc8eucV_16_HRSCAF_138__chr1	24035795	DEL	383	0.306888	LSAMP	-150407	GAP43	481899
Sc8eucV_41_HRSCAF_239__chr2	61742801	DEL	699	0.306143	CDKAL1	0	CDKAL1	0
Sc8eucV_18_HRSCAF_152__chr22	4681012	DEL	145	0.305817	LOC104693230	0	LOC104693230	0
Sc8eucV_19_HRSCAF_154__chr3	91662759	DEL	122	0.305817	LOC104693575	-4823	LOC104693569	6609
Sc8eucV_19_HRSCAF_154__chr3	59802741	INS	52	0.3027	EPB41L2	-985	SMLR1	118199
Sc8eucV_20_HRSCAF_160__chr1A	72391930	DEL	67	0.3027	CACNA1C	0	CACNA1C	0
Sc8eucV_28_HRSCAF_204__chrZ	41910148	DEL	58	0.3027	SLC12A2	-121109	LOC104691976	24998
Sc8eucV_28_HRSCAF_204__chrZ	658358	INS	220	0.3027	TCF4	0	TCF4	0
Sc8eucV_16_HRSCAF_138__chr1	57452531	DEL	1336	0.301669	DGKH	-1802	RGCC	329040
Sc8eucV_2_HRSCAF_5__chr5	45053086	DEL	136	0.301417	LRP5	0	LRP5	0

Supplementary Table 5.

Detailed sample information on sequenced and mapped individuals.

Genus	Species	Individual ID	Tissue	Sampling location	Sequencing Instrument
Corvus	brachyrhynchos	USA_CA_B01	tissue, blood	USA, California, Shasty County	Illumina HiSeq2000
Corvus	brachyrhynchos	USA_CA_B03	tissue, blood	USA, California, Shasta, Cottonwood	Illumina HiSeq2000
Corvus	brachyrhynchos	USA_CA_B08	unknown	USA, California	Illumina HiSeq2000
Corvus	brachyrhynchos	USA_NJ_B02	tissue, blood	USA, New Jersey, Union County, Westfield	Illumina HiSeq2000
Corvus	brachyrhynchos	USA_NY_B03	tissue, blood	USA, New York, Suffolk County, Northport	Illumina HiSeq2000
Corvus	brachyrhynchos	USA_NY_B04	tissue, blood	USA, New York, Nassau County, Wantagh	Illumina HiSeq2000
Corvus	corone cornix	B_So_H01	blood	Bulgaria, Sofia	Illumina HiSeq2000
Corvus	corone cornix	B_So_H02	blood	Bulgaria, Sofia	Illumina HiSeq2000
Corvus	corone cornix	B_So_H03	blood	Bulgaria, Sofia	Illumina HiSeq2000
Corvus	corone cornix	B_So_H04	blood	Bulgaria, Sofia	Illumina HiSeq2000
Corvus	corone cornix	B_SZ_H01	blood	Bulgaria, Stora Zagora	Illumina HiSeq2000
Corvus	corone cornix	B_SZ_H02	blood	Bulgaria, Stora Zagora	Illumina HiSeq2000
Corvus	corone cornix	B_un_H01	blood	Bulgaria	Illumina HiSeq2000
Corvus	corone cornix	ISR_TA_H01	blood	Israel, Tel Aviv	Illumina HiSeq2000
Corvus	corone cornix	ISR_TA_H02	blood	Israel, Tel Aviv	Illumina HiSeq2000
Corvus	corone cornix	ISR_TA_H04	blood	Israel, Tel Aviv	Illumina HiSeq2000
Corvus	corone cornix	ITA_Ro_H01	blood	Italy, Rome	Illumina HiSeq2000
Corvus	corone cornix	ITA_Ro_H02	blood	Italy, Rome	Illumina HiSeq2000
Corvus	corone cornix	ITA_Ro_H03	blood	Italy, Rome	Illumina HiSeq2000
Corvus	corone cornix	ITA_Ro_H04	blood	Italy, Rome	Illumina HiSeq2000
Corvus	corone cornix	ITA_Ro_H05	blood	Italy, Rome	Illumina HiSeq2000
Corvus	corone cornix	ITA_Ro_H06	blood	Italy, Rome	Illumina HiSeq2000
Corvus	corone cornix	ITA_Ro_H07	blood	Italy, Rome	Illumina HiSeq2000
Corvus	corone cornix	ITA_Ro_H08	blood	Italy, Rome	Illumina HiSeq2000
Corvus	corone cornix	ITA_Ro_H09	tissue	Italy, Rome	Illumina HiSeq2000
Corvus	corone cornix	ITA_Ro_H10	tissue	Italy, Rome	Illumina HiSeq2000

Corvus	corone cornix	ITA_Ro_H11	tissue	Italy, Rome	Illumina HiSeq2000
Corvus	corone cornix	ITA_Ro_H12	tissue	Italy, Rome	Illumina HiSeq2000
Corvus	corone cornix	ITA_Ro_H13	tissue	Italy, Rome	Illumina HiSeq2000
Corvus	corone cornix	ITA_Ro_H14	tissue	Italy, Rome	Illumina HiSeq2000
Corvus	corone cornix	PL_Wa_H02	blood	Poland, Warsaw	Illumina HiSeq2000
Corvus	corone cornix	PL_Wa_H03	blood	Poland, Warsaw	Illumina HiSeq2000
Corvus	corone cornix	PL_Wa_H05	blood	Poland, Warsaw	Illumina HiSeq2000
Corvus	corone cornix	PL_Wa_H06	blood	Poland, Warsaw	Illumina HiSeq2000
Corvus	corone cornix	PL_Wa_H09	blood	Poland, Warsaw	Illumina HiSeq2000
Corvus	corone cornix	PL_Wa_H11	blood	Poland, Warsaw	Illumina HiSeq2000
Corvus	corone cornix	PL_Wa_H14	blood	Poland, Warsaw	Illumina HiSeq2000
Corvus	corone cornix	PL_Wa_H16	blood	Poland, Warsaw	Illumina HiSeq2000
Corvus	corone cornix	PL_Wa_H17	blood	Poland, Warsaw	Illumina HiSeq2000
Corvus	corone cornix	PL_Wa_H22	blood	Poland, Warsaw	Illumina HiSeq2000
Corvus	corone cornix	PL_Wa_H23	blood	Poland, Warsaw	Illumina HiSeq2000
Corvus	corone cornix	PL_Wa_H32	blood	Poland, Warsaw	Illumina HiSeq2000
Corvus	corone cornix	PL_Wa_H35	blood	Poland, Warsaw	Illumina HiSeq2000
Corvus	corone cornix	PL_Wa_H50	blood	Poland, Warsaw	Illumina HiSeq2000
Corvus	corone cornix	PL_Wa_H52	blood	Poland, Warsaw	Illumina HiSeq2000
Corvus	corone cornix	RUS_Ki_H02	blood	Russia, Kirov	Illumina HiSeq2000
Corvus	corone cornix	RUS_Ki_H03	blood	Russia, Kirov	Illumina HiSeq2000
Corvus	corone cornix	RUS_Ki_H04	blood	Russia, Kirov	Illumina HiSeq2000
Corvus	corone cornix	RUS_No_H02	blood	Russia, Novosibirsk	Illumina HiSeq2000
Corvus	corone cornix	RUS_No_H03	blood	Russia, Novosibirsk	Illumina HiSeq2000
Corvus	corone cornix	RUS_Tu_H01	blood	Russia, Tyumen	Illumina HiSeq2000
Corvus	corone cornix	S_Ri_H05	liver	Sweden, Rimbo	Illumina HiSeq2000
Corvus	corone cornix	S_Ri_H07	liver	Sweden, Rimbo	Illumina HiSeq2000
Corvus	corone cornix	S_Ri_H23	liver	Sweden, Rimbo	Illumina HiSeq2000
Corvus	corone cornix	S_Ri_H29	liver	Sweden, Rimbo	Illumina HiSeq2000

Corvus	corone cornix	S_Ri_H43	liver	Sweden, Rimbo	Illumina HiSeq2000
Corvus	corone cornix	S_Up_H03	blood	Sweden, Uppsala	Illumina HiSeq2000
Corvus	corone cornix	S_Up_H09	blood	Sweden, Uppsala	Illumina HiSeq2000
Corvus	corone cornix	S_Up_H16	blood	Sweden, Uppsala	Illumina HiSeq2000
Corvus	corone cornix	S_Up_H24	blood	Sweden, Uppsala	Illumina HiSeq2000
Corvus	corone cornix	S_Up_H29	blood	Sweden, Uppsala	Illumina HiSeq2000
Corvus	corone cornix	S_Up_H37	blood	Sweden, Uppsala	Illumina HiSeq2000
Corvus	corone cornix	S_Up_H43	blood	Sweden, Uppsala	Illumina HiSeq2000
Corvus	corone cornix	S_Up_H47	blood	Sweden, Uppsala	Illumina HiSeq2000
Corvus	corone cornix	S_Up_H51	blood	Sweden, Uppsala	Illumina HiSeq2000
Corvus	corone cornix	S_Up_H52	blood	Sweden, Uppsala	Illumina HiSeq2000
Corvus	corone corone	D_Ko_C02	blood	Germany, Konstanz	Illumina HiSeq2000
Corvus	corone corone	D_Ko_C04	blood	Germany, Konstanz	Illumina HiSeq2000
Corvus	corone corone	D_Ko_C05	blood	Germany, Konstanz	Illumina HiSeq2000
Corvus	corone corone	D_Ko_C08	blood	Germany, Konstanz	Illumina HiSeq2000
Corvus	corone corone	D_Ko_C11	blood	Germany, Konstanz	Illumina HiSeq2000
Corvus	corone corone	D_Ko_C13	blood	Germany, Konstanz	Illumina HiSeq2000
Corvus	corone corone	D_Ko_C15	blood	Germany, Konstanz	Illumina HiSeq2000
Corvus	corone corone	D_Ko_C19	blood	Germany, Konstanz	Illumina HiSeq2000
Corvus	corone corone	D_Ko_C20	blood	Germany, Konstanz	Illumina HiSeq2000
Corvus	corone corone	D_Ra_C01	blood	Germany, Radolfzell	Illumina HiSeq2000
Corvus	corone corone	D_Ra_C05	blood	Germany, Radolfzell	Illumina HiSeq2000
Corvus	corone corone	D_Ra_C06	blood	Germany, Radolfzell	Illumina HiSeq2000
Corvus	corone corone	D_Ra_C11	blood	Germany, Radolfzell	Illumina HiSeq2000
Corvus	corone corone	D_Ra_C14	blood	Germany, Radolfzell	Illumina HiSeq2000
Corvus	corone corone	D_Ra_C16	blood	Germany, Radolfzell	Illumina HiSeq2000
Corvus	corone corone	E_Vi_C01	blood	Spain, Villaseca de la Sorriba	Illumina HiSeq2000
Corvus	corone corone	E_Vi_C05	blood	Spain, Villaseca de la Sorriba	Illumina HiSeq2000
Corvus	corone corone	E_Vi_C08	blood	Spain, Villaseca de la Sorriba	Illumina HiSeq2000

Corvus	corone corone	E_Vi_C14	blood	Spain, Villaseca de la Sorriba	Illumina HiSeq2000
Corvus	corone corone	E_Vi_C19	blood	Spain, Villaseca de la Sorriba	Illumina HiSeq2000
Corvus	corone corone	E_Vi_C22	blood	Spain, Villaseca de la Sorriba	Illumina HiSeq2000
Corvus	corone corone	E_Vi_C23	blood	Spain, Villaseca de la Sorriba	Illumina HiSeq2000
Corvus	corone corone	E_Vi_C32	blood	Spain, Villaseca de la Sorriba	Illumina HiSeq2000
Corvus	corone corone	E_Vi_C37	blood	Spain, Villaseca de la Sorriba	Illumina HiSeq2000
Corvus	corone corone	E_Vi_C44	blood	Spain, Villaseca de la Sorriba	Illumina HiSeq2000
Corvus	corone corone	E_Vi_C46	blood	Spain, Villaseca de la Sorriba	Illumina HiSeq2000
Corvus	corone corone	E_Vi_C48	blood	Spain, Villaseca de la Sorriba	Illumina HiSeq2000
Corvus	corone corone	E_Vi_C51	blood	Spain, Villaseca de la Sorriba	Illumina HiSeq2000
Corvus	corone corone	E_Vi_C57	blood	Spain, Villaseca de la Sorriba	Illumina HiSeq2000
Corvus	corone corone	E_Vi_C58	blood	Spain, Villaseca de la Sorriba	Illumina HiSeq2000
Corvus	corone coroneXcorone cornix	IRL_Lm_H07	blood	Ireland, Lough Money Farm	Illumina HiSeq2000
Corvus	corone coroneXcorone cornix	IRL_Lm_H08	blood	Ireland, Lough Money Farm	Illumina HiSeq2000
Corvus	corone coroneXcorone cornix	IRL_Lm_H10	blood	Ireland, Lough Money Farm	Illumina HiSeq2000
Corvus	corone coroneXcorone cornix	IRL_Lm_H12	blood	Ireland, Lough Money Farm	Illumina HiSeq2000
Corvus	corone coroneXcorone cornix	IRL_Lm_H15	blood	Ireland, Lough Money Farm	Illumina HiSeq2000
Corvus	corone coroneXcorone cornix	IRL_Lm_H16	blood	Ireland, Lough Money Farm	Illumina HiSeq2000
Corvus	corone coroneXcorone cornix	RUS_Ke_Y01	blood	Russia, Kemerovo	Illumina HiSeq2000
Corvus	corone coroneXcorone cornix	RUS_Ke_Y02	blood	Russia, Kemerovo	Illumina HiSeq2000

Corvus	corone coroneXcorone cornix	RUS_Ke_Y03	blood	Russia, Kemerovo	Illumina HiSeq2000
Corvus	corone coroneXcorone cornix	RUS_Ke_Y05	blood	Russia, Kemerovo	Illumina HiSeq2000
Corvus	corone coroneXcorone cornix	RUS_Ke_Y06	blood	Russia, Kemerovo	Illumina HiSeq2000
Corvus	corone orientalis	RUS_Kr_O01	blood	Russia, Krasnoyarsky	Illumina HiSeq2000
Corvus	corone orientalis	RUS_Kr_O02	blood	Russia, Krasnoyarsky	Illumina HiSeq2000
Corvus	corone orientalis	RUS_Kr_O03	blood	Russia, Krasnoyarsky	Illumina HiSeq2000
Corvus	corone orientalis	RUS_Kr_O04	blood	Russia, Krasnoyarsky	Illumina HiSeq2000
Corvus	corone orientalis	RUS_Pr_O01	tissue, blood	Russia, Primorsky	Illumina HiSeq2000
Corvus	corone orientalis	RUS_Pr_O02	tissue, blood	Russia, Primorsky	Illumina HiSeq2000
Corvus	corone orientalis	RUS_Pr_O03	tissue, blood	Russia, Primorsky	Illumina HiSeq2000
Corvus	corone orientalis	RUS_Pr_O04	tissue, blood	Russia, Primorsky	Illumina HiSeq2000
Corvus	corone orientalis	RUS_Pr_O05	tissue, blood	Russia, Primorsky	Illumina HiSeq2000
Corvus	corone orientalis	RUS_Tv_O01	blood	Russia, Tuva	Illumina HiSeq2000
Corvus	corone orientalis	RUS_Tv_O02	blood	Russia, Tuva	Illumina HiSeq2000
Corvus	corone orientalis	RUS_Ya_O01	tissue	Russia, Yakutsk	Illumina HiSeq2000
Corvus	corone orientalis	RUS_Ya_O02	blood	Russia, Yakutsk	Illumina HiSeq2000
Corvus	corone orientalis	RUS_Ya_O03	blood	Russia, Yakutsk	Illumina HiSeq2000
Corvus	dauuricus	CHN_Gu_D02	liver	China, Guangxi	Illumina HiSeq2000
Corvus	dauuricus	MON_Kh_D01	blood	Mongolia, Khentii	Illumina HiSeq2000
Corvus	dauuricus	MON_Kh_D02	blood	Mongolia, Khentii	Illumina HiSeq2000
Corvus	dauuricus	MON_Kh_D03	blood	Mongolia, Khentii	Illumina HiSeq2000
Corvus	monedula	S_Ri_J01	liver	Sweden, Rimbo	Illumina HiSeq2000
Corvus	monedula	S_Ri_J02	liver	Sweden, Rimbo	Illumina HiSeq2000
Corvus	monedula	S_Ri_J03	liver	Sweden, Rimbo	Illumina HiSeq2000
Corvus	monedula	S_Ri_J08	liver	Sweden, Rimbo	Illumina HiSeq2000

Corvus	pectoralis/torquatus	CHN_Gu_P01	tissue	China, Guangxi	Illumina HiSeq2000
Corvus	pectoralis/torquatus	Un_un_P01	tissue	unknown	Illumina HiSeq2000
Corvus	pectoralis/torquatus	Un_un_P02	tissue	unknown	Illumina HiSeq2000
Corvus	corone cornix	S_Up_H32	blood	Sweden,Uppsala	PacBio RSII
Corvus	corone cornix	S_Up_H03	blood	Sweden,Uppsala	PacBio Sequel
Corvus	corone cornix	S_Up_H47	blood	Sweden,Uppsala	PacBio RSII
Corvus	corone cornix	S_Up_H37	blood	Sweden,Uppsala	PacBio Sequel
Corvus	corone cornix	S_Up_H24	blood	Sweden,Uppsala	PacBio RSII
Corvus	corone cornix	S_Up_H29	blood	Sweden,Uppsala	PacBio RSII
Corvus	corone cornix	S_Up_H59	blood	Sweden,Uppsala	PacBio Sequel
Corvus	corone cornix	Pl_Wa_H22	blood	Poland,Warsaw	PacBio RSII
Corvus	corone cornix	Pl_Wa_H24	blood	Poland,Warsaw	PacBio RSII
Corvus	corone corone	D_Ko_C04	blood	Germany,Konstanz	PacBio RSII
Corvus	corone corone	D_Ko_C13	blood	Germany,Konstanz	PacBio RSII
Corvus	corone corone	D_Ko_C15	blood	Germany,Konstanz	PacBio Sequel
Corvus	corone corone	D_Ko_C29	blood	Germany,Konstanz	PacBio RSII
Corvus	corone corone	D_Ra_C16	blood	Germany,Radolfzell	PacBio Sequel
Corvus	corone corone	D_Ko_C36	blood	Germany,Konstanz	PacBio Sequel
Corvus	corone corone	D_Ra_C05	blood	Germany,Radolfzell	PacBio Sequel
Corvus	corone corone	D_Ko_C31	blood	Germany,Konstanz	PacBio Sequel
Corvus	corone corone	E_Vi_C57	blood	Spain,La Sorriba	PacBio RSII
Corvus	corone corone	E_Vi_C58	blood	Spain,La Sorriba	PacBio RSII
Corvus	corone corone	E_Vi_C98	blood	Spain,La Sorriba	PacBio Sequel
Corvus	corone corone	E_Vi_C100	blood	Spain,La Sorriba	PacBio Sequel
Corvus	corone corone	E_Vi_C101	blood	Spain,La Sorriba	PacBio Sequel
Corvus	corone corone	E_Vi_C103	blood	Spain,La Sorriba	PacBio RSII
Corvus	brachyrhynchos	USA_Wa_B01	blood	USA,Seattle	PacBio RSII
Corvus	brachyrhynchos	USA_Wa_B02	blood	USA,Seattle	PacBio RSII
Corvus	monedula	S_Up_J01	blood	Sweden,Uppsala	PacBio RSII

Corvus	monedula	S_To_J13	blood	Sweden,Aspa	PacBio Sequel
Corvus	monedula	S_To_J10	blood	Sweden,Aspa	PacBio Sequel
Corvus	monedula	S_To_J14	blood	Sweden,Aspa	PacBio RSII
Corvus	monedula	S_To_J15	blood	Sweden,Aspa	PacBio RSII
Corvus	dauuricus	RUS_Mp_D04	blood	Russia,Muraviovka Park	PacBio Sequel
Corvus	dauuricus	RUS_Mp_D06	blood	Russia,Muraviovka Park	PacBio Sequel
Corvus	dauuricus	RUS_Mp_D08	blood	Russia,Muraviovka Park	PacBio Sequel

Supplementary Table 6.
Statistics of optical maps and map assemblies.

Sample	Species	Data over 150 kb	N50 (Mb)	Maprate to *Corvus cornix* assembly	N50 (Mb)	Map length (Mb)	Effective coverage
D_Ko_C04	*Corvus corone corone*	58 Gbp	0.1426	49.70%	0.223	529.43	19.89
D_Ko_C13	*Corvus corone corone*	48.0589 Gbp	0.1978	54.30%	0.4	1291.57	32.78
D_Ko_C29	*Corvus corone corone*	113.2390 Gbp	0.2164	45.70%	0.647	1154.888	47.49
D_Ko_C36	*Corvus corone corone*	141.6023 Gbp	0.2278	69.00%	1.044	1236.42	82.23
D_Ra_C05	*Corvus corone corone*	313.7037 Gbp	0.2272	52.40%	0.759	1202.661	106.71
E_Vi_C101	*Corvus corone corone*	221.5878 Gbp	0.2566	71.30%	1.064	1306.967	130.50
E_Vi_C103	*Corvus corone corone*	194.30 Gbp	0.2516	65.20%	0.848	1207.24	93.00
E_Vi_C57	*Corvus corone corone*	356.49 Gbp	0.2167	55.80%	0.726	1449.28	166.50
E_Vi_C58	*Corvus corone corone*	149.5706 Gbp	0.2111	44.00%	0.755	1135.633	44.05
S_Up_H03	*Corvus corone cornix*	178.58 Gbp	0.2311	66.50%	0.826	1192.34	89.78
S_Up_H29	*Corvus corone cornix*	141.49 Gbp	0.2488	70.70%	0.824	1203.84	85.12
S_Up_H32	*Corvus corone cornix*	107.2551 Gbp	0.2171	67.20%	0.763	1108.333	59.74
S_Up_H37	*Corvus corone cornix*	166.40 Gbp	0.2411	68.40%	0.829	1216.61	99.61
S_Up_H59	*Corvus corone cornix*	132.0199 Gbp	0.2109	50.60%	0.798	1136.396	75.20
S_Up_J01	*Corvus monedula*	188.0292 Gbp	0.2438	29.30%	0.812	1201.969	73.28
S_To_J17	*Corvus monedula*	200.8957 Gbp	0.2336	36.40%	0.882	1309.932	94.89

Supplementary Table 7.
Detailed annotation of manually curated repeats.

Class	Subclass	Superfamily	Superfamily	Family	Subfamily	Similarity to Repbase repeats (100 bp)	Comment	Consensus status	Consensus length	TSD
Retrotransposon	LTR	ERV2	ERVK	corCor_ERV_chimera	corCor_ERV_chimera-head_corCorLTRK1b-L		corCorLTRK1b and TguERV1-L arranged in head-to-head chimera. Full chimera consensus incomplete on both ends and split into two at the ERVK/ERV1 boundary.	Incomplete 5' end	2072	?
Retrotransposon	LTR	ERV1	ERV1	corCor_ERV_chimera	corCor_ERV_chimera-tail_TguERV1-L_corCor	TguERV1_I (80%)	corCorLTRK1b and TguERV1-L arranged in head-to-head chimera. Full chimera consensus incomplete on both ends and split into two at the ERVK/ERV1 boundary.	Incomplete 3' end	3950	?
Retrotransposon	LTR	ERV?	ERV?	corCorERV	corCorERV-1	None		Complete	599	4 or 5
Retrotransposon	LTR	ERV1	ERV1	corCorLTR1	corCorLTR1	None		Complete	461	4
Retrotransposon	LTR	ERV1	ERV1	corCorLTR2	corCorLTR2	None		Complete	467	4
Retrotransposon	LTR	ERV2	ERVK	corCorLTRK10	corCorLTRK10	None		Complete	653	6
Retrotransposon	LTR	ERV2	ERVK	corCorLTRK11	corCorLTRK11	None		Complete	458	6
Retrotransposon	LTR	ERV2	ERVK	corCorLTRK12	corCorLTRK12	None		Complete	481	6
Retrotransposon	LTR	ERV2	ERVK	corCorLTRK13	corCorLTRK13	None		Complete	575	6
Retrotransposon	LTR	ERV2	ERVK	corCorLTRK14	corCorLTRK14	None		Complete	681	6
Retrotransposon	LTR	ERV2	ERVK	corCorLTRK15	corCorLTRK15	None		Complete	966	6
Retrotransposon	LTR	ERV2	ERVK	corCorLTRK16	corCorLTRK16	None		Complete	339	6
Retrotransposon	LTR	ERV2	ERVK	corCorLTRK17	corCorLTRK17_I	TguERVK8_I + TguERVK9_I (67% + 76%)		Complete	5032	6
Retrotransposon	LTR	ERV2	ERVK	corCorLTRK17	corCorLTRK17_LTR	None		Complete	375	6
Retrotransposon	LTR	ERV2	ERVK	corCorLTRK18	corCorLTRK18	None		Complete	540	6
Retrotransposon	LTR	ERV2	ERVK	corCorLTRK19	corCorLTRK19	None		Complete	510	6
Retrotransposon	LTR	ERV2	ERVK	corCorLTRK1	corCorLTRK1a_I	ERV2-1_CJ-I + hAT-9_Ami (75% + 67%)		Complete	1736	6
Retrotransposon	LTR	ERV2	ERVK	corCorLTRK1	corCorLTRK1a_LTR	None		Complete	289	6

Retrotransposon	LTR	ERV2	ERVK	corCorLTRK1	corCorLTRK1b_I	MUDRN3_OS + L1-11_NN (72% + 74%)		Complete	1661	6
Retrotransposon	LTR	ERV2	ERVK	corCorLTRK1	corCorLTRK1b_LTR	None		Complete	294	6
Retrotransposon	LTR	ERV2	ERVK	corCorLTRK1	corCorLTRK1c_I	MERMITE18D + L1-11_NN (68% + 74%)		Complete	1675	6
Retrotransposon	LTR	ERV2	ERVK	corCorLTRK1	corCorLTRK1c_LTR	None		Complete	288	6
Retrotransposon	LTR	ERV2	ERVK	corCorLTRK1	corCorLTRK1d_I	L1-11_NN (72%)		Complete	1420	
Retrotransposon	LTR	ERV2	ERVK	corCorLTRK1	corCorLTRK1d_LTR	None		Complete	299	6
Retrotransposon	LTR	ERV2	ERVK	corCorLTRK20	corCorLTRK20	None		Complete	728	6
Retrotransposon	LTR	ERV2	ERVK	corCorLTRK21	corCorLTRK21	None		Complete	434	6
Retrotransposon	LTR	ERV2	ERVK	corCorLTRK22	corCorLTRK22	None		Complete	545	6
Retrotransposon	LTR	ERV2	ERVK	corCorLTRK2	corCorLTRK2a	None		Complete	365	6
Retrotransposon	LTR	ERV2	ERVK	corCorLTRK2	corCorLTRK2b	None		Complete	365	6
Retrotransposon	LTR	ERV2	ERVK	corCorLTRK2	corCorLTRK2c	None		Complete	352	6
Retrotransposon	LTR	ERV2	ERVK	corCorLTRK3	corCorLTRK3a	None		Complete	367	6
Retrotransposon	LTR	ERV2	ERVK	corCorLTRK3	corCorLTRK3b	None		Complete	365	6
Retrotransposon	LTR	ERV2	ERVK	corCorLTRK4	corCorLTRK4a1	hAT-N15_XL (70%)		Complete	421	6
Retrotransposon	LTR	ERV2	ERVK	corCorLTRK4	corCorLTRK4a2	hAT-N2_CPB (70%)		Complete	429	6
Retrotransposon	LTR	ERV2	ERVK	corCorLTRK5	corCorLTRK5a	None		Complete	375	6
Retrotransposon	LTR	ERV2	ERVK	corCorLTRK5	corCorLTRK5b	None		Complete	374	6
Retrotransposon	LTR	ERV2	ERVK	corCorLTRK6	corCorLTRK6a	None		Complete	437	6
Retrotransposon	LTR	ERV2	ERVK	corCorLTRK6	corCorLTRK6b	None		Complete	438	6
Retrotransposon	LTR	ERV2	ERVK	corCorLTRK7	corCorLTRK7a	RNLTR2d (72%)		Complete	633	6
Retrotransposon	LTR	ERV2	ERVK	corCorLTRK7	corCorLTRK7b	None		Complete	628	6
Retrotransposon	LTR	ERV2	ERVK	corCorLTRK8	corCorLTRK8a	None		Complete	663	6
Retrotransposon	LTR	ERV2	ERVK	corCorLTRK8	corCorLTRK8b	L1-65_Acar (76%)		Complete	689	6
Retrotransposon	LTR	ERV2	ERVK	corCorLTRK9	corCorLTRK9	ATCOPIA49_I (72%)		Complete	306	6
Retrotransposon	LTR	ERV3	ERVL	corCorLTRL10	corCorLTRL10	None		Complete	709	5
Retrotransposon	LTR	ERV3	ERVL	corCorLTRL11	corCorLTRL11	None		Complete	665	5
Retrotransposon	LTR	ERV3	ERVL	corCorLTRL12	corCorLTRL12	None		Complete	693	5
Retrotransposon	LTR	ERV3	ERVL	corCorLTRL13	corCorLTRL13	LTR-29C_Gav (77%)		Complete	902	5
Retrotransposon	LTR	ERV3	ERVL	corCorLTRL1	corCorLTRL1a	None		Complete	374	5
Retrotransposon	LTR	ERV3	ERVL	corCorLTRL1	corCorLTRL1b	None		Complete	374	5
Retrotransposon	LTR	ERV3	ERVL	corCorLTRL2	corCorLTRL2a	CR1-5_Psi (70%)		Complete	585	5
Retrotransposon	LTR	ERV3	ERVL	corCorLTRL2	corCorLTRL2b	None		Complete	600	5

Retrotransposon	LTR	ERV3	ERVL	corCorLTRL3	corCorLTRL3	None		Complete	1175	5
Retrotransposon	LTR	ERV3	ERVL	corCorLTRL4	corCorLTRL4	None		Complete	1171	5
Retrotransposon	LTR	ERV3	ERVL	corCorLTRL5	corCorLTRL5	None		Complete	783	5
Retrotransposon	LTR	ERV3	ERVL	corCorLTRL6	corCorLTRL6	MurERV4 + ERV1-4_Crp-I (75% + 74%)		Complete	875	5
Retrotransposon	LTR	ERV3	ERVL	corCorLTRL7	corCorLTRL7	None		Complete	675	5
Retrotransposon	LTR	ERV3	ERVL	corCorLTRL8	corCorLTRL8	hAT-5_Sbi (72%)		Complete	898	5
Retrotransposon	LTR	ERV3	ERVL	corCorLTRL9	corCorLTRL9	None		Complete	457	5
Retrotransposon	LINE	CR1	CR1	CR1-E_Pass	CR1-E_Pass-L_corCor.inc	CR1-E_Pass (87%)		Incomplete 5' end	325	?
Satellite	Satellite	Satellite	Satellite	crowSat1	crowSat1	None		Complete	13934	
Satellite?	Satellite?	Satellite?	Satellite?	crowSat2	crowSat2	TE-X-12_DR (62%)		Incomplete 5' and 3' ends	3784	?
Satellite?	Satellite?	Satellite?	Satellite?	crowSat3	crowSat3	Copia-53_ZM-I + Harbinger-2_Crp + CR1-C4 + Keno-1_Ssa (78% + 75% + 67%)		Incomplete 5' and 3' ends	4412	?
Retrotransposon	LTR	ERV1	ERV1	ERV1-1_NuM	ERV1-1_NuM-L_corCor	ERV1-1_NuM-LTR (75%)		Complete	537	4
Retrotransposon	LTR	ERV3	ERVL	Tgu_rep3	Tgu_rep3-La_corCor	Tgu_rep3 + TguLTR11 (75% + 70%)		Complete	997	5
Retrotransposon	LTR	ERV3	ERVL	Tgu_rep3	Tgu_rep3-Lb_corCor	Tgu_rep3 (83%)		Complete	1026	5
Retrotransposon	LTR	ERV1	ERV1	TguERV1	TguERV1-L_corCor.inc	TguERV1_I (80%)		Incomplete 5' and 3' ends	7043	?
Retrotransposon	LTR	ERV1	ERV1	TguERV7	TguERV7_I-La_corCor	TguERV7k_I (71%)		Complete	494	4
Retrotransposon	LTR	ERV1	ERV1	TguERV7	TguERV7_I-Lb_corCor	TguERV7k_I (74%)		Complete	552	4
Retrotransposon	LTR	ERV3	ERVL	TguERVK5	TguERVK5-L_corCor	TguERVK5_LTR1c (68%)		Complete	540	5
Retrotransposon	LTR	ERV2	ERVK	TguERVK7	TguERVK7-La_corCor	TguERVK7_LTR2b + TguERVK7_LTR4 (80% + 74%)		Complete	670	6
Retrotransposon	LTR	ERV2	ERVK	TguERVK7	TguERVK7-Lb_corCor	TguERVK7_LTR4 (70%)		Complete	709	6
Retrotransposon	LTR	ERV3	ERVL	TguERVL2	TguERVL2-La_corCor	TguERVL2a3_LTR + TguERVL2b1_LTR (81% + 80%)		Complete	564	6
Retrotransposon	LTR	ERV3	ERVL	TguERVL2	TguERVL2-Lb_corCor	TguERVL2a2-LTR + LTR-12_Ami (85% + 68%)		Complete	1178	5
Retrotransposon	LTR	ERV3	ERVL	TguERVL2	TguERVL2-Lc_corCor	TguERVL2b1_LTR (83%)		Complete	589	5
Retrotransposon	LTR	ERV3?	ERVL?	TguERVL2	TguERVL2-Ld_corCor.inc	TguERVL2_I (79%)		Incomplete 5' and 3' ends	5523	?

Retrotransposon	LTR	ERV3	ERVL	TguERVL2	TguERVL2-Le_corCor	TguERVL2a2-LTR + MuDR-N18C_OS (83% + 76%)		Complete	906	5
Retrotransposon	LTR	ERV3	ERVL	TguERVL2	TguERVL2-Lf_corCor	TguERVL2a2-LTR + MuDR-N18C_OS (90% + 76%)		Complete	909	5
Retrotransposon	LTR	ERV3	ERVL	TguERVL2	TguERVL2-Lg_corCor	TguERVL2a2-LTR + MuDR-N18C_OS (90 % + 76%)		Complete	908	5
Retrotransposon	LTR	ERV1	ERV1	TguLTR12	TguLTR12-L_corCor	TguLTR12 (75%)		Complete	594	4
Retrotransposon	LTR	ERV2	ERVK	TguLTRK4	TguLTRK4-L_corCor	TguLTRK4c (72%)		Complete	439	6
Retrotransposon	LTR	ERV2	ERVK	TguLTRK5	TguLTRK5-L_corCor	TguLTRK5d (69%)		Complete	648	6
Retrotransposon	LTR	ERV2	ERVK	TguLTRK9	TguLTRK9-L_corCor	TguLTRK9b (65%)		Complete	608	6
Retrotransposon	LTR	ERV3	ERVL	TguLTRL1	TguLTRL1-La_corCor	TguLTRL1a6 + TguLTRL1a2 + TguLTRL1a7 (78% + 72% + 78%)		Complete	640	5
Retrotransposon	LTR	ERV3	ERVL	TguLTRL1	TguLTRL1-Lb_corCor	TguLTRL1a7 (73%)		Complete	625	5
Retrotransposon	LTR	ERV3	ERVL	TguLTRL1	TguLTRL1-Lc_corCor	TguLTRL1a6 (67%)		Complete	600	5
Retrotransposon	LTR	ERV3	ERVL	TguLTRL2	TguLTRL2-La_corCor	Tgu_rep2 + TguLTRL2a8 (66% + 68%)		Complete	1303	5
Retrotransposon	LTR	ERV3	ERVL	TguLTRL2	TguLTRL2-Lb_corCor	None		Complete	131	5
Retrotransposon	LTR	ERV3	ERVL	TguLTRL2	TguLTRL2-Lc_corCor	Tgu_rep2 + TguLTRL2a7 (66% + 67%)		Complete	1315	5
Retrotransposon	LTR	ERV3	ERVL	TguLTRL3	TguLTRL3-La_corCor	TguLTRL3b2 (73%)		Complete	520	4
Retrotransposon	LTR	ERV3?	ERV3?	TguLTRL3	TguLTRL3-Lb_corCor	TguLTRL3c (79%)		Complete	660	4 or 5
Retrotransposon	LTR	ERV3	ERVL	TguLTRL4	TguLTRL4-L_corCor	TguLTRL4a + hAT-N58_CPB + TguLTRL4a (75% + 73% + 84%)		Complete	1400	5
Retrotransposon	LTR	ERV3	ERVL	TguLTRL6	TguLTRL6-La_corCor.inc	TguLTRL6b + TguERVL1_I + ERV3-1_MUn-I (93% + 65% + 68%)		Incomplete 5' and 3' ends	2025	?
Retrotransposon	LTR	ERV3	ERVL	TguLTR6	TguLTRL6-Lb_corCor	TguLTR6a (87%)		Complete	268	5?
