# Supplementary material for: Discovery and population genomics of structural variation in a songbird genus
Source: Nat Commun. 2020 Jul 7;11:3403. doi: 10.1038/s41467-020-17195-4 (PMC7341801; doi:10.1038/s41467-020-17195-4)
Supplement: Supplementary file 3 — Reporting Summary [file 41467_2020_17195_MOESM3_ESM.pdf]

nature research

Corresponding author(s): Matthias H. Weissensteiner, Jochen B. W. Wolf

Last updated by author(s): Apr 21, 2020

Reporting Summary

Nature Research wishes to improve the reproducibility of the work that we publish. This form provides structure for consistency and transparency in reporting. For further information on Nature Research policies, see Authors & Referees and the Editorial Policy Checklist.

Statistics

For all statistical analyses, confirm that the following items are present in the figure legend, table legend, main text, or Methods section.

n/a	Confirmed	
☐	☒	The exact sample size (n) for each experimental group/condition, given as a discrete number and unit of measurement
☐	☒	A statement on whether measurements were taken from distinct samples or whether the same sample was measured repeatedly
☐	☒	The statistical test(s) used AND whether they are one- or two-sided<br>*Only common tests should be described solely by name; describe more complex techniques in the Methods section.*
☐	☒	A description of all covariates tested
☐	☒	A description of any assumptions or corrections, such as tests of normality and adjustment for multiple comparisons
☐	☒	A full description of the statistical parameters including central tendency (e.g. means) or other basic estimates (e.g. regression coefficient) AND variation (e.g. standard deviation) or associated estimates of uncertainty (e.g. confidence intervals)
☐	☒	For null hypothesis testing, the test statistic (e.g. F, t, r) with confidence intervals, effect sizes, degrees of freedom and P value noted<br>*Give P values as exact values whenever suitable.*
☒	☐	For Bayesian analysis, information on the choice of priors and Markov chain Monte Carlo settings
☒	☐	For hierarchical and complex designs, identification of the appropriate level for tests and full reporting of outcomes
☒	☐	Estimates of effect sizes (e.g. Cohen's d, Pearson's r), indicating how they were calculated

Our web collection on statistics for biologists contains articles on many of the points above.

Software and code

Policy information about availability of computer code

Data collection	Data collection of PacBio sequencing was performed with SMRT Analysis software (v2.3.0) suite on RSII and Sequel instruments. BioNano optical mapping was performed on the Irys instrument followed by image analysis with the IrysView software. DoveTail Hi-C data were analyzed and assembled using the DoveTail HiRise software.
Data analysis	Proprietary software: DoveTail HiRise. Publicly available software used in this study: FALCON UNZIP v0.4.0, ARROW, BUSCO v2.0.1, Bionano Solve pipeline 3.3.1 (pipeline version 7841), LASTZ v1.04.00., MUMmer v3.23, Assemblytics (assemblytics.com), SURVIVOR (v1.0.3), BLASTN (v2.2.26), MAFFT (v6), CENSOR (http://www.girinst.org/censor/index.php), NGM-LR (v0.2.2), samtools (v1.9), Sniffles (v1.0.8), bcftools (v1.9), vcftools (v0.1.15), R (v3.2.3, R Core Team), lme4 package (v1.1-19), BWA-MEM (v0.7.17), Lumpy (v0.2.12), Delly (v0.7.7), Manta (v1.0.3), SNPrelate, (v1.4.2.), gdsfmt (v1.6.2),

For manuscripts utilizing custom algorithms or software that are central to the research but not yet described in published literature, software must be made available to editors/reviewers. We strongly encourage code deposition in a community repository (e.g. GitHub). See the Nature Research guidelines for submitting code & software for further information.

Data

Policy information about availability of data

All manuscripts must include a data availability statement. This statement should provide the following information, where applicable:

- Accession codes, unique identifiers, or web links for publicly available datasets
- A list of figures that have associated raw data
- A description of any restrictions on data availability

All raw data generated in this study is currently being uploaded to the NCBI Sequence Read Archive, NCBI GenBank and Data Dryad, respectively.

Field-specific reporting

Please select the one below that is the best fit for your research. If you are not sure, read the appropriate sections before making your selection.

☐ Life sciences ☐ Behavioural & social sciences ☒ Ecological, evolutionary & environmental sciences

For a reference copy of the document with all sections, see nature.com/documents/nr-reporting-summary-flat.pdf

Ecological, evolutionary & environmental sciences study design

All studies must disclose on these points even when the disclosure is negative.

Study description	To comprehensively assess genomic structural variation (SV) in natural populations and investigate its population dynamics, we compiled a data set consisting of individuals of different species and populations of the songbird genus Corvus, sequenced with Illumina short-read sequencing, PacBio long-read sequencing and BioNano optical mapping. We used genome-assembly and read mapping approaches to investigate the full size spectrum of SV and identify variants with potential phenotypic effects.
Research sample	In total 153 different individuals from 6 species of the songbird genus Corvus from Europe, North America and Asia have been used in this study.
Sampling strategy	We mostly sampled blood samples from caught individuals. For sampling details, see Supplementary Table S5.
Data collection	MW, WH, and JW conducted field work and provided samples.
Timing and spatial scale	Samples were taken from Spring 2014 to Summer 2017.
Data exclusions	A single individual was excluded from the long-read data set due to a mislabeled sampling tube.
Reproducibility	No experiments were carried out in this study.
Randomization	Not applicable to this study.
Blinding	All analyses were performed blind in respect to the outcome.

Did the study involve field work? ☒ Yes ☐ No

Field work, collection and transport

Field conditions	Not relevant for the current study because we focus on genetic data.
Location	Samples were obtained in Sweden (Uppsala, Aspa, Rimbo), Germany (Radolfzell, Konstanz), Spain (La Sorriba), Poland (Warsaw), Russia (Muraviovka Park), USA (Seattle)
Access and import/export	Permissions for sampling of wild crows were granted by Regierungspräsidium Freiburg (Aktenzeichen: 55-8852.15), by Jordbruksverket (Dnr 30-1326/10) in Sweden and by Muraviovka Park in Russia. Polish hooded crow nestlings were provided by courtesy of Dr. Andrzej Kruszewicz from the animal rehabilitation centre of Warsaw Zoo.
Disturbance	Samples were either taken from nestlings and they were released after handling or from captive individuals meant for common garden experiments (for a different study).

Reporting for specific materials, systems and methods

We require information from authors about some types of materials, experimental systems and methods used in many studies. Here, indicate whether each material, system or method listed is relevant to your study. If you are not sure if a list item applies to your research, read the appropriate section before selecting a response.

Materials & experimental systems

n/a	Involved in the study	
☒	☐	Antibodies
☒	☐	Eukaryotic cell lines
☒	☐	Palaeontology
☐	☒	Animals and other organisms
☒	☐	Human research participants
☒	☐	Clinical data

Methods

n/a	Involved in the study	
☒	☐	ChIP-seq
☒	☐	Flow cytometry
☒	☐	MRI-based neuroimaging

Animals and other organisms

Policy information about studies involving animals; ARRIVE guidelines recommended for reporting animal research

Laboratory animals	No laboratory animals have been used in this study.
Wild animals	Wild individuals of the songbird genus Corvus have been caught in the wild. For sampling locations, species and population see Supplementary table S5
Field-collected samples	Not applicable.
Ethics oversight	Permissions for sampling of wild crows were granted by Regierungspräsidium Freiburg (Aktenzeichen: 55-8852.15), by Jordbruksverket (Dnr 30-1326/10) in Sweden and by Muraviovka Park in Russia. Polish hooded crow nestlings were provided by courtesy of Dr. Andrzej Kruszewicz from the animal rehabilitation centre of Warsaw Zoo.

Note that full information on the approval of the study protocol must also be provided in the manuscript.
