# Supplementary material for: Discovery and population genomics of structural variation in a songbird genus
Source: Nat Commun. 2020 Jul 7;11:3403. doi: 10.1038/s41467-020-17195-4 (PMC7341801; doi:10.1038/s41467-020-17195-4)
Supplement: Supplementary file 4 — Description of Additional Supplementary Files [file 41467_2020_17195_MOESM4_ESM.pdf]

Description of Additional Supplementary Files

File name: Supplementary Data 1
VCF file containing long-read based SV calls used for downstream analysis.

File name: Supplementary Data 2
VCF file containing short-read based SV calls used for downstream analysis.

File name: Supplementary Data 3
FASTA file containing manually repeat motifs.

File name: Supplementary Data 4
VCF file containing optical mapping-based SV calls used for downstream analysis.

File name: Supplementary Data 5
Pdf and Rmd file containing all scripts, workflows and custom code.
