# Supplementary material for: Discovery and population genomics of structural variation in a songbird genus
Source: Nat Commun. 2020 Jul 7;11:3403. doi: 10.1038/s41467-020-17195-4 (PMC7341801; doi:10.1038/s41467-020-17195-4)
Supplement: Supplementary file 2 — Peer Review [file 41467_2020_17195_MOESM2_ESM.pdf]

Reviewers' comments:

Reviewer #1 (Remarks to the Author):

Summary:

This study uses the latest advances in genomic tools to reconstruct high-quality genome assemblies for the hooded crow (Corvus (corone) cornix) and the European jackdaw (Corvus monedula). These high-quality genomes are used as a reference to study the population genomics of structural variation. Such a study of structural variation across evolutionary timescales is an important advance in the field. Most importantly, the study finds a cis-acting 2.25-kb retrotransposon insertion reducing expression of the NDP gene potentially involved in plumage color variation.

Minor comments:

1) What types of variation are included when authors refer to Structural variation? Do they use the definition from Freeman, J.L. et al. Copy number variation: new insights in genome diversity. Genome Res. 16(8), 949-61 (2006)?

2) Line 42: "..constraints have long impeded genome-wide characterization of..". The sentence seems incomplete?

3) Custom scripts and other technical details accompanying a manuscript can be provided as a resource on dryad, figshare or some such public repository. A link to the same can be provided in the manuscript. While this is becoming standard practice, it is up to the author's discretion.

4) The use of multiple genomics technologies for performing genome assemblies is very interesting. A schematic describing the approach and its novelty in comparison to other approaches might be helpful to understand the manuscript better.

5) Do the authors test any specific evolutionary hypothesis regarding the Structural Variation in the current manuscript? This needs to be better described.

Major issues:

1) Genome-wide numbers of SV (Structural variation) and SNPs per 1 Mb window are compared and their density histograms are presented in Figure 1. However, given that the lengths of SV's have considerable heterogeneity, it might be appropriate to measure SV density after correcting for SV length.

If the authors had a different reason for looking solely at counts/Mb, it would be good to know this rationale. In the supplementary figure S1, the length distributions from LR and OM are provided. This again raises the question of the technical reliability of comparing SV counts/MB.

Moreover, the comparison of SV density across species without considering the length distribution of SV's in these species may need to be revisited. The authors are motivated to understand the evolutionary properties of SV population genetics. Hence, the length distributions of SV's seem important. For example see (The origin, evolution, and functional impact of short insertion-deletion variants identified in 179 human genomes, doi: 10.1101/gr.148718.112) who state that "We find that indel length modulates selection strength and that indels affecting multiple functionally constrained nucleotides undergo stronger purifying selection."

2) Mapping rate of optical maps

"Table S6.Statistics of optical maps and map assemblies" provides details of the Maprate to Corvus cornix assembly. The values range from 29.3% to 70.7% within the Swedish population. Why do the mapping rates differ so much? Can the authors provide an explanation for this?

The high heterogeneity in the mapping rate is not only intriguing but might be of relevance to the results being presented. For example, are the differences in the mapping rate reflective of differences in the quality of the data between samples of the optical mapping experiment? Does this also affect the discovery rate of structural variation?

It has been seen that the unmapped fraction in NGS datasets correspond to the "dark matter" of the genome. Is it possible that the unmapped fraction corresponds to different arrangments of the DNA? For example, see this paper about what long-read sequencing has revealed about the chloroplast genome (https://academic.oup.com/gbe/article/11/12/3372/5637229).

3) Association between NDP gene expression and LTR insertion

The authors report a striking association between the expression level of the NDP gene and LTR insertion. It would be interesting if the authors explored the link between LTR insertion and the regulatory elements that are being affected. Which regulatory element is being affected?

In addition to the gene expression level association, are the authors able to demonstrate differential binding of a regulatory protein or some other functional data to support the association identified by the current manuscript?

Reviewer #2 (Remarks to the Author):

This manuscript NCOMMS-19-39605-T studies the genomic structural variation in Corvus genus. The dataset is really impressive and the study is interesting. However, how the manuscript was written I wasn’t sure if it should be treated more methodological (how to find SVs using different sequencing data) or evolutionary (Corvus population genetics and evolution). Based on the title I first thought this would be about the birds but there wasn’t anything why to study SV in Corvus genus especially in the abstract or introduction paragraph. For this manuscript to be suitable for publication at Nature Communications, re-writing is really needed.

Below I have some specific comments to improve the manuscript.

Abstract: Try to decide if the main message of this study is methodological or evolutionary. If it’s the first then tell why SV detection needs improvement. If it’s about Corvus, then tell why to study SV in these birds. If it’s combination of both then try to implement both aspects in the abstract (and the whole manuscript).

Main text: Please add the main headings (introduction, results and discussion) and also sub-headings to guide the reader.

Page 2-3, lines 40-51 (introduction paragraph): Add more information about what SVs are, why to

study those especially in the non-model system and why in Corvus if you will tell the story from the Corvus side. Add also a “in this study…” section so the introduction links to the results section.

Page 3, line 53 onwards, the result section: The subheadings are especially needed here as you have quite a lot of results to cover (maybe some could go to supplements?). Have first an introduction paragraph about the results and then go section by section through the results.

Figure 1a: this is really good and needed overview of the samples.

Table 1: the first part is about SVs and the second is SNPs? Or other way around? Add column titles.

Figure 4a: Are the PCA axis titles correct? Shouldn’t they be PC1-PC5?

Methods: Because you have a long methods section as well, a workflow figure would be really helpful in understanding what is happening. Try also keep the same order both in the MM and the results parts.

We would like to thank the two anonymous referees for their helpful and encouraging comments. Please find below the detailed responses to each comment.

Reviewers' comments:

Reviewer #1 (Remarks to the Author):

Summary:

This study uses the latest advances in genomic tools to reconstruct high-quality genome assemblies for the hooded crow (Corvus (corone) cornix) and the European jackdaw (Corvus monedula). These high-quality genomes are used as a reference to study the population genomics of structural variation. Such a study of structural variation across evolutionary timescales is an important advance in the field. Most importantly, the study finds a cis-acting 2.25-kb retrotransposon insertion reducing expression of the NDP gene potentially involved in plumage color variation.

Minor comments:

R1.1 What types of variation are included when authors refer to Structural variation? Do they use the definition from Freeman, J.L. et al. Copy number variation: new insights in genome diversity. Genome Res. 16(8), 949-61 (2006)?

We now provide an explicit definition of what we understand by SV following Alkan et al. 2011. Note however, that due to the specific analysis requirements, we have confined our analysis to insertions, deletions and inversions.

Lines 57-60:
Structural mutations altering more than 50 bp of DNA sequence at once include deletions, insertions, inversions, transposable element insertions, duplications and translocations, and have the potential to drastically change phenotypes with medical and evolutionary implications 1–4.

R1.2 Line 42: "..constraints have long impeded genome-wide characterization of..". The sentence seems incomplete?

We thank the reviewer for pointing this out. This is now fixed:

Lines 60-62:
Yet, technological constraints have long impeded genome-wide characterization of structural mutations segregating in populations (structural variation, hereafter referred to as SV) 5.

R1.3 Custom scripts and other technical details accompanying a manuscript can be provided as a resource on dryad, figshare or some such public repository. A link to the same can be provided in the manuscript. While this is becoming standard practice, it is up to the author's discretion.

All scripts and pipelines used in this manuscript are freely available on a GitHub repository https://github.com/EvoBioWolf/Corvid_SVpopgen which is now specified in a 'Data and Code Availability' statement. Upload of the genome assemblies and newly generated raw data to the sequence read archive are currently ongoing.

R1.4 The use of multiple genomics technologies for performing genome assemblies is very interesting. A schematic describing the approach and its novelty in comparison to other approaches might be helpful to understand the manuscript better.

We thank the reviewer for this helpful comment, which was similarly raised by reviewer 2 (R2.8) and will help the reader orient. We have now included workflow figures for the generation of the reference assembly (Supplementary Figure S1), the assembly-based SV detection (Supplementary Figure S2) and the long-read based SV detection (Supplementary Figure S5). These figures are accordingly referred to in the main text (lines 117, 124, 145)

R1.5 Do the authors test any specific evolutionary hypothesis regarding the Structural Variation in the current manuscript? This needs to be better described.

As also mentioned by reviewer 2, the structure of the original manuscript did not convey the goals of the study appropriately. In a first step, the study explores the methodological aspect of conducting large-scale population analyses across evolutionary time scales using SV instead of SNPs. After demonstrating that this is feasible, we used population genetic approaches to specifically screen for putative causal structural mutations contributing to reproductive isolation in a well-established model of incipient speciation. We have now extended the abstract, introduction and discussion substantially to better convey the motivation of the study.

Abstract (lines 36-52)
Structural variation (SV) is an important component of genetic mutations providing the raw material for evolution. Here, we uncovered the genome-wide spectrum of intra- and interspecific SV segregating in natural populations of seven songbird species in the genus *Corvus*. […] We exploited sampling across wide phylogenetic timescales to validate SV genotypes and demonstrated their suitability for standard population genetic analyses. We then assessed the contribution of this hitherto hidden form of genetic variation to evolutionary processes in an avian model of incipient speciation, the *Corvus (corone)* spp. species complex. […]

Introduction (76-108): In this study, we characterize SV in natural populations across scales of integration (populations, subspecies, species) and utilize them for evolutionary inference. As model, we use the avian songbird genus *Corvus* comprising a total of 40 species including crows, ravens and jackdaws [16]. Central to this system is the phylogenetically independent recurrence of a pied colour-pattern in several species that stands in contrast to the predominant all-black plumage in the clade (**Fig. 1A**). Colour divergence is believed to contribute to prezygotic isolation by means of social and sexual selection promoting speciation [17]. The gentic basis of plumage variation and its contribution to population divergence has been investigated in much detail using single-nucleotide polymorphism (SNP) data in the *Corvus (corone) spp.* species complex that is characterized by narrow contact zones between all-black forms (*C. (c.) corone/orientalis*) and populations consisting of the pied phenotype (*C. (c.) cornix/capellanus/pallescens/pectoralis/sharpii*) [18–20]. These studies suggest that phenotypically well differentiated populations diverged during the late

Pleistocene and exhibit low levels of genome-wide differentiation [21]. Only few, narrow genomic regions associated with morphological contrast are subject to divergent selection reducing local gene flow and increasing genetic differentiation [21,22]. In the European hybrid zone comprising carrion crows (*C . (c.) corone*) and hooded crows (*C. (c.) cornix*) epistasis between a genetic factor on chromosome 18 and the gene *NDP* located on chromsome 1 explained most of the morphological variation [23] which can be near-exclusively related to gene expression differences in melanocytes of nascent feathers [24,25]. Despite some indication for a structural rearrangement underlying phenotypic divergence in two European crow populations [22], the overall extent and role of SV in population divergence remains unclear in this system.

To fill this gap and more broadly investigate the dynamics of SV in natural populations, we compiled short-read, long-read and optical mapping data for populations of seven species from the genus *Corvus*. We generated genome assemblies for two of the species spanning the evolutionary history of the clade, comprehensively characterized SV using read-mapping and assembly-based approaches and used phylogenetically informed filtering to obtain reliable SV genotypes. After the establishment of these genomic resources, we demonstrate the suitability of SV for population genetic analysis and evolutionary inference using the *Corvus (corone)* spp. species complex.

Discussion (292-298):
SV constitutes a large proportion of genetic variation in the genomes of eukaryotes organisms [45] affecting phenotypes with fitness consequences [1]. It is thus imperative to investigate the extent and occurrence of SV beyond well-studied model organisms and characterize its dynamics in naturally evolving populations. The study presented here illustrates the feasibility of SV characterization followed by downstream phylogentic and population analyses in a non-model organism without reliance on any prior genetic resources. […]

Major issues:

R1.6 Genome-wide numbers of SV (Structural variation) and SNPs per 1 Mb window are compared and their density histograms are presented in Figure 1. However, given that the lengths of SV's have considerable heterogeneity, it might be appropriate to measure SV density after correcting for SV length.

If the authors had a different reason for looking solely at counts/Mb, it would be good to know this rationale. In the supplementary figure S1, the length distributions from LR and OM are provided. This again raises the question of the technical reliability of comparing SV counts/MB.

Moreover, the comparison of SV density across species without considering the length distribution of SV's in these species may need to be revisited. The authors are motivated to understand the evolutionary properties of SV population genetics. Hence, the length distributions of SV's seem important. For example see (The origin, evolution, and functional impact of short insertion-deletion variants identified in 179 human genomes, doi: 10.1101/gr.148718.112) who state that "We find that indel length modulates selection strength and that indels affecting multiple functionally constrained nucleotides undergo stronger purifying selection."

This is an important point which we have addressed as follows: When determining the number of SVs per 1 Mb window after aligning the two haplotypes of the assembly, we sought to provide a simple measure of genetic diversity based on SVs. Since the SV counts stem from non-overlapping variants, there is no possibility of double counts compromising this measure due to large variant size, making it essentially length-insensitive.
Nevertheless, to investigate the influence of SV size on this measure of genetic diversity, we calculated genome-wide numbers of SV per 1 Mb windows for four different size categories (50 - 500 bp, 500 – 1000 bp, 1000 – 5000 bp and > 5000 bp) which we present in the revised results section and in a new Supplementary Figure S4. Genetic diversity estimated from these different size categories was generally consistent and seems to be relatively independent of size. Regardless of the length category, we found a picture of reduced genetic variation in the highly inbred Hawaiian crow. Note, however, that due to the drastically decreasing number of variants for sizes beyond 1 kb, the resolution in these size classes is limited.
When comparing SV size distribution between species, we found a density peak for variants ~ 2.3 kb of size was prominent in hooded and Hawaiian crow, but not the jackdaw (Supplementary Figure S3). It is possible that an overrepresentation of a certain variant size may distort the estimation of genome-wide genetic diversity based on SVs, however, since the majority of variants was smaller than 500 bp in all three species (79.44, 84.31 and 76.74 % for hooded crow, jackdaw and Hawaiian crow, respectively), this seems unlikely.

We have now incorporated these changes in the main text:
Lines 124-132:
"Median sizes of SVs ranged from 111 bp in jackdaw to 158 bp in Hawaiian crow, with all three species showing similar SV length distributions (**Supplementary Fig. 3**). As a proxy for genetic diversity within a single individual, we recorded genome-wide densities of SNPs and non-overlapping SVs in 1 Mb windows (**Fig. 1B)**. Total as well as per-window numbers of SV and SNPs were highest in jackdaw and lowest in the highly inbred Hawaiian crow (across four SV size categories, **Supplementary Fig. 4**), consistent with a positive correlation between census population size and genetic diversity [29,30]."

R1.7 Mapping rate of optical maps

"Table S6.Statistics of optical maps and map assemblies" provides details of the Maprate to Corvus cornix assembly. The values range from 29.3% to 70.7% within the Swedish population. Why do the mapping rates differ so much? Can the authors provide an explanation for this?

The high heterogeneity in the mapping rate is not only intriguing but might be of relevance to the results being presented. For example, are the differences in the mapping rate reflective of differences in the quality of the data between samples of the optical mapping experiment? Does this also affect the discovery rate of structural variation?

It has been seen that the unmapped fraction in NGS datasets correspond to the "dark matter" of the genome. Is it possible that the unmapped fraction corresponds to different arrangments of the DNA? For example, see this paper about what long-read sequencing has revealed about the chloroplast genome (https://academic.oup.com/gbe/article/11/12/3372/5637229).

We thank the reviewer for pointing this out. The main reason for the large variation in map rate is that the two individuals with the lowest map rate (S_Up_J01 and S_To_J17 with 29.3 and 36.4 %, respectively) belong to a different species than the reference (jackdaw, *C.*

monedula). The map rate to a diverged reference is accordingly expected to be lower. We apologize for not including this information in Supplementary table S6 and have added a column with the species information.

Apart from that, we agree with the reviewer that map rate is also reflective of data quality and amount of data to some extent, which may indeed influence the discovery of SV. This is likely to become less of a problem with increasing number of individuals. It is also likely that at least a proportion of unaligned optical maps consists of complex DNA repeats and rearrangements. However, it is very difficult to determine this fraction, since the ground truth is inaccessible in this case.

R1.8 Association between NDP gene expression and LTR insertion

The authors report a striking association between the expression level of the NDP gene and LTR insertion. It would be interesting if the authors explored the link between LTR insertion and the regulatory elements that are being affected. Which regulatory element is being affected?

R1.9
In addition to the gene expression level association, are the authors able to demonstrate differential binding of a regulatory protein or some other functional data to support the association identified by the current manuscript?

R1.8 & 1.9: We thank the reviewer for these suggestions. The crow is not a genetic model system, and hence there are hardly any functional genetic resources available. Our ability to further investigate functional elements is therefore limited. To address whether the LTR retrotransposon insertion may be associated with function, we investigated the degree of evolutionary conservation in this region. By aligning 5 kb of unique flanking sequence on either side of the LTR retrotransposon insertion in the hooded crow genome to the chicken reference genome (galGal6), we identified a highly conserved non-coding region present in the 3' flanking sequence of the LTR retrotransposon insertion (2.8 kb distance to the insertion, Figure 4B and Supplementary Figure S9). This sequence is highly conserved between birds and mammals and is thus a good candidate for a regulatory element in the vicinity to the LTR insertion. As such it may affect the expression of the *NDP* gene. We must admit however, that with an absence of functional experiments (and such are unfeasible in crow), the actual effect of the LTR insertion remains speculative.

We have added these analyses to the methods and results and included an additional figure (Supplementary Figure S9)

Lines 281-284
"The insertion lies upstream of *NDP* in close proximity (2.8 kb) to a highly conserved non-coding region in vertebrate genomes (likely constituting a regulatory element, **Supplementary Fig. 9**) as well as an orthologous region in pigeons where a copy number variation modulates plumage patterning (**Fig. 4B**) [40]."

Methods, lines 574-579:
To identify highly conserved regions indicative of regulatory elements in the vicinity of the LTR retrotransposon insertion, we aligned 5 kb of the flanking sequence on either site of the insertion in the hooded crow genome to the chicken reference assembly (galGal6) on the

UCSC GenomeBrowser (https://genome.ucsc.edu). Visual inspection of conservation scores and human chain alignment tracks revealed a highly conserved region in the 3' flanking sequence (**Supplementary Fig. 9**)."

Reviewer #2 (Remarks to the Author):

This manuscript NCOMMS-19-39605-T studies the genomic structural variation in Corvus genus. The dataset is really impressive and the study is interesting. However, how the manuscript was written I wasn't sure if it should be treated more methodological (how to find SVs using different sequencing data) or evolutionary (Corvus population genetics and evolution). Based on the title I first thought this would be about the birds but there wasn't anything why to study SV in Corvus genus especially in the abstract or introduction paragraph. For this manuscript to be suitable for publication at Nature Communications, re-writing is really needed.

Below I have some specific comments to improve the manuscript.

R2.1 Abstract: Try to decide if the main message of this study is methodological or evolutionary. If it's the first then tell why SV detection needs improvement. If it's about Corvus, then tell why to study SV in these birds. If it's combination of both then try to implement both aspects in the abstract (and the whole manuscript).

We thank the reviewer for this helpful comment. Indeed, this manuscript is intended to cover both the methodological as well as the evolutionary aspects of SV. We agree that this may have not been clear enough in the original version of the manuscript. We have now extended the abstract, introduction and discussion substantially and also altered the title to better convey the two-fold motivation of the study.

Title;
"Discovery and population genomics of structural variation in a songbird genus"

Abstract (lines 36-52)
2Structural variation (SV) is an important component of genetic mutations providing the raw material for evolution. Here, we uncovered the genome-wide spectrum of intra- and interspecific SV segregating in natural populations of seven songbird species in the genus *Corvus*. […] We exploited sampling across wide phylogenetic timescales to validate SV genotypes and demonstrated their suitability for standard population genetic analyses. We then assessed the contribution of this hitherto hidden form of genetic variation to evolutionary processes in an avian model of incipient speciation, the *Corvus (corone)* spp. species complex. […]"

Introduction (lines 76-108):
"In this study, we characterize SV in natural populations across scales of integration (populations, subspecies, species) and utilize them for evolutionary inference. As model, we use the avian songbird genus *Corvus* comprising a total of 40 species including crows, ravens and jackdaws [16]. Central to this system is the phylogenetically independent recurrence of a pied colour-pattern in several species that stands in contrast to the predominant all-black plumage in the clade (**Fig. 1A**). Colour divergence is believed to contribute to prezygotic isolation by means of social and sexual selection promoting speciation [17]. The gentic basis of

plumage variation and its contribution to population divergence has been investigated in much detail using single-nucleotide polymorphism (SNP) data in the *Corvus (corone) spp.* species complex that is characterized by narrow contact zones between all-black forms (*C. (c.) corone/orientalis*) and populations consisting of the pied phenotype (*C. (c.) cornix/capellanus/pallescens/pectoralis/sharpii*) [18–20]. These studies suggest that phenotypically well differentiated populations diverged during the late Pleistocene and exhibit low levels of genome-wide differentiation [21]. Only few, narrow genomic regions associated with morphological contrast are subject to divergent selection reducing local gene flow and increasing genetic differentiation [21,22]. In the European hybrid zone comprising carrion crows (*C . (c.) corone*) and hooded crows (*C. (c.) cornix*) epistasis between a genetic factor on chromosome 18 and the gene *NDP* located on chromsome 1 explained most of the morphological variation [23] which can be near-exclusively related to gene expression differences in melanocytes of nascent feathers [24,25]. Despite some indication for a structural rearrangement underlying phenotypic divergence in two European crow populations [22], the overall extent and role of SV in population divergence remains unclear in this system. “

Discussion (292-298):
“SV constitutes a large proportion of genetic variation in the genomes of eukaryotes organisms [45] affecting phenotypes with fitness consequences [1]. It is thus imperative to investigate the extent and occurrence of SV beyond well-studied model organisms and characterize its dynamics in naturally evolving populations. The study presented here illustrates the feasibility of SV characterization followed by downstream phylogentic and population analyses in a non-model organism without reliance on any prior genetic resources. […]”

R2.2 Main text: Please add the main headings (introduction, results and discussion) and also sub-headings to guide the reader.

Done.

R2.3 Page 2-3, lines 40-51 (introduction paragraph): Add more information about what SVs are, why to study those especially in the non-model system and why in Corvus if you will tell the story from the Corvus side. Add also a “in this study…” section so the introduction links to the results section.

This is a very helpful suggestion from the reviewer. Indeed, the lack of the abovementioned information stems from the size constraints in a previous submission. As explained above, we now introduce the crow system more thoroughly and extended the section on *lines 76-108.*

R2.4
Page 3, line 53 onwards, the result section: The subheadings are especially needed here as you have quite a lot of results to cover (maybe some could go to supplements?). Have first an introduction paragraph about the results and then go section by section through the results.

We have now added subheadings and included a paragraph summarizing and structuring the expected results. Furthermore we have added a paragraph to improve the transition from introduction to results.

Lines 101-108:

“To fill this gap and more broadly investigate the dynamics of SV in natural populations, we compiled short-read, long-read and optical mapping data for populations of seven species from the genus *Corvus*. We generated genome assemblies for two of the species spanning the evolutionary history of the clade, comprehensively characterized SV using read-mapping and assembly-based approaches and used phylogenetically informed filtering to obtain reliable SV genotypes. After the establishment of these genomic resources, we demonstrate the suitability of SV for population genetic analysis and evolutionary inference using the *Corvus (corone)* spp. species complex. “

R2.5 Figure 1a: this is really good and needed overview of the samples.

We thank the reviewer for this comment. To further improve this figure, we have now added drawings of all species to illustrate the phenotypic contrast.

R2.6 Table 1: the first part is about SVs and the second is SNPs? Or other way around? Add column titles.

For clarity, we have now added column titles in Table 1.

R2.7 Figure 4a: Are the PCA axis titles correct? Shouldn’t they be PC1-PC5?

There was indeed a mistake here in the caption which may have prompted the confusion. Middle and left panels are subsets of the original data set (middle panel: crow clade only, right panel: European populations of crow clade only). For each of the subsets we performed independent PCAs and then plotted PC1 against PC2. We have now changed this in the figure caption accordingly:

Lines 872-877:
“**(A)** Principal component analysis based on SV genotypes. In the *left panel*, all individuals were analyzed together. Individuals of the crow clade are tightly clustered and separate from the jackdaw clade along PC1; individuals of the two jackdaw species separate along PC2. The *middle panel* displays the results of PCA conducted exclusively for individuals from the crow clade, clearly separating American crows from the Eurasian species. In the *right panel*, only the European populations of the crow clade are included, showing marked separation of the Spanish carrion crow population.”

R2.8 Methods: Because you have a long methods section as well, a workflow figure would be really helpful in understanding what is happening. Try also keep the same order both in the MM and the results parts.

We thank the reviewer for this helpful comment, which was similarly raised by reviewer 1 and will help the reader orient. We have now included workflow figures for the generation of the reference assembly (Supplementary Figure S1), the assembly-based SV detection (Supplementary Figure S2) and the long-read based SV detection (Supplementary Figure S5). These figures are accordingly referred to in the main text (*lines 117, 124, 145*).

We further aligned the order of results and methods section as good as possible.

REVIEWERS' COMMENTS:

Reviewer #1 (Remarks to the Author):

The previous version of the article was reviewed by me as reviewer-1. Authors have answered all the questions raised by both the reviewers.

This study establishes the relevance of structural variation in causing phenotypic differences in natural populations. The use of optical mapping data from multiple individuals to study the population genetics of SV is also a very interesting aspect of this study. The authors acknowledge the challenges involved in SV detection. Hence, this manuscript would also serve as good starting point for future efforts at SV detection in natural populations.

Line 26-27: Affiliation #10 is provided. However, none of the authors listed seem to have this superscript.

Few grammatical errors need to be taken care of. For example,

Line 295-296: "..eukaryotes organisms.." can be changed to "..eukaryotic organisms.."

Reviewer #2 (Remarks to the Author):

The authors did an excellent job responding to the concerns and suggestions. I have no further comments for the authors.

We thank the two anonymous referees for their encouraging comments and have addressed their comments accordingly below.

REVIEWERS' COMMENTS:

Reviewer #1 (Remarks to the Author):

The previous version of the article was reviewed by me as reviewer-1. Authors have answered all the questions raised by both the reviewers.

This study establishes the relevance of structural variation in causing phenotypic differences in natural populations. The use of optical mapping data from multiple individuals to study the population genetics of SV is also a very interesting aspect of this study. The authors acknowledge the challenges involved in SV detection. Hence, this manuscript would also serve as good starting point for future efforts at SV detection in natural populations.

Line 26-27: Affiliation #10 is provided. However, none of the authors listed seem to have this superscript.

This is fixed now.

Few grammatical errors need to be taken care of. For example,

Line 295-296: "..eukaryotes organisms.." can be changed to "..eukaryotic organisms.."

Done.

Reviewer #2 (Remarks to the Author):

The authors did an excellent job responding to the concerns and suggestions. I have no further comments for the authors.
