# Supplementary material for: Discovery and population genomics of structural variation in a songbird genus
Source: Nat Commun. 2020 Jul 7;11:3403. doi: 10.1038/s41467-020-17195-4 (PMC7341801; doi:10.1038/s41467-020-17195-4)
Supplement: Supplementary file 5 — Supplementary Dataset 1-5 [file 41467_2020_17195_MOESM5_ESM.zip › Supplementary Data 5.zip!/Custom_scripts_and_pipelines_Weissensteiner_et_al.pdf]

Custom scripts and pipelines

The population genomics of structural variation in a songbird genus

Matthias H. Weissensteiner

January 8, 2020

Contents

Assembly-based SV detection **1**
SV detection with MUMMer and Assemblytics . . . 1
SV detection with smartie-sv . . . 2
Merge Assemblytics and smartie-sv output . . . 2
Assembly-based SV visualization and density per 1 Mb window . . . 2

Assembly-based SNP detection **3**

Read-mapping based SV detection **4**
Long-read mapping based SV Detection . . . 4
Downstream analysis of LR variants in R . . . 6
PCA . . . 7
'Phylogenetic filtering' . . . 9
SV lengths by LR and OM . . . 14
Repeat characterization of LR insertions and deletions: . . . 15
Site frequency spectrum of LR variants . . . 16
Short-read based SV detection . . . 24

FST analysis **25**

Gene expression of the *NDP* gene **27**

Repeat characterization pipeline **28**

Assembly-based SV detection

SV detection with MUMMer and Assemblytics

First align the associated contigs of the FALCON UNZIP assembly to the primary assembly:

```
MUMmer3.23/nucmer -maxmatch -l 100 -c 500 \
   primary_assembly.fas associated_contigs.fas \
   -prefix associated_vs_primary_assembly
```

The resulting delta file was then uploaded to the Assemblytics webpage (www.assemblytics.com) where the genome comparison was run at default parameters. The Assemblytics output was filtered and converted to vcf using SURVIVOR:

```
SURVIVOR convertAssemblytics assemblytics_output 0 temp

cat temp | tr ";" "\t" | sed 's/SVLEN=//g' | awk '$13<2000' | \
 awk '{print $1 "\t" $2 "\t" $3 "\t"$4 "\t" $5 "\t" $6 "\t" $7 "\t" $8 ";" $9 ";"
 $10 ";" $11 ";" $12 ";SVLEN=" $13 ";" $14 "\t" $15}' > assemblytics_output.vcf
```

SV detection with smartie-sv

We ran `smartie-sv` with the default Snakefile using primary and associated assemblies as input. The output was filtered and converted to vcf with `SURVIVOR`:

```
snakemake -p -w 25 -j 3 \
  --verbose \
  -s Snakefile \
  --cluster-config cluster.config.slurm.json \
  --cluster "sbatch -A {cluster.partition} -n {cluster.n}  -t {cluster.time} -c {cluster.c} \
  -o out -e error"
  -w 30
awk '{print $1,$2,$3,$1,$2+$5,$3+$5,$4,$4,$13,$6,$14}' smartie-sv_output  | \
sed 's/ /\t/g' | sed -e 's/insertion/INS/g' -e 's/deletion/DEL/g'  > temp
SURVIVOR bedpetovcf smartie-sv_output.vcf
```

Merge Assemblytics and smartie-sv output

This was done using `SURVIVOR`:

```
SURVIVOR merge \
  1000 \
  1 \
  1 \
  0 \
  0 \
  30 \
  associated_contigs_vs_primary_assembly_Assemblytics_smartiesv.vcf

grep -v '#' associated_contigs_vs_primary_assembly_Assemblytics_smartiesv.vcf | \
 tr ";" "\t" | cut -f 1,2,5,10 | sed -e 's/AVGLEN=//g' -e 's/<//g' -e 's/>//g' > \
 associated_contigs_vs_primary_assembly_Assemblytics_smartiesv.table
```

Assembly-based SV visualization and density per 1 Mb window

```
#loading the data
dat_JD<-read.table("associated_contigs_vs_primary_assembly_Assemblytics_smartiesv.table", header=F)
colnames(dat_JD)<-c("chr", "pos", "type", "len")
win<-read.table("1Mb_windows.genome.bed", header=F)
colnames(win)<-c("chr", "start", "end")
chr_lengths<-read.table("genome_file.txt", header=T)

# Filter for full 1 Mb windows:

win %>% mutate(win_length=end-start) %>%
  filter(win_length == 1000000) -> filtered_win
count<-c()
average_len<-c()
for (i in 1:length(filtered_win$chr)) {
  dat_JD %>%
    filter(chr == paste(filtered_win[i,1]) & pos >= filtered_win[i,2] & pos <= filtered_win[i,3]) %>%
    select(len) %>%
```

```
    unlist() %>%
    as.vector() %>%
    length() -> count[i]
  dat_JD %>%
    filter(chr == paste(filtered_win[i,1]) & pos >= filtered_win[i,2] & pos <= filtered_win[i,3]) %>%
    select(len) %>%
    unlist() %>%
    as.vector() %>%
    mean() -> average_len[i]
}

filtered_win %>% mutate(count, average_len) -> filtered_win

xlabel<-"Count"
ylabel<-"Frequency"
title<-"SV count per 1Mb window"
filtered_win %>%
  ggplot(aes(count)) +
  geom_histogram(binwidth = 1,colour="#000000", fill="#bdbdbd", aes(y=..density..), size=0.2) +
  xlim(-2,25) +
  theme_classic(base_size = 9) +
  xlab(xlabel)+
  ylab(ylabel)+
  theme(
        strip.text = element_text(),
        plot.margin = unit(c(5,5,5,5),"mm"),
        panel.spacing.x = unit(2,"mm"),
        legend.position="none",
        axis.title.x=element_text(size=12),
        axis.title.y=element_blank(),
        axis.text.x=element_text(size=9),
        axis.text.y=element_text(size=9))
```

Assembly-based SNP detection

This was done using the `show-snps` function of `MUMmer`, following alignment of the primary and associated assemblies:

```
MUMmer3.23/nucmer -maxmatch -l 100 -c 100 \
primary_assembly.fas associated_contigs.fas -prefix associated_vs_primary_assembly
delta-filter -r -q associated_vs_primary_assembly > associated_vs_primary_assembly.filter
show-snps -Clr -T associated_vs_primary_assembly.filter > \
associated_vs_primary_assembly.filter.snps
```

Then the visualization and SNP density calculation was done in `R`:

```
# Load data
dat<-read.table("associated_vs_primary_assembly.filter.snps", header=F)
colnames(dat)<-c("chr", "pos", "end")
win<-read.table("1Mb_windows.genome.bed", header=F)
colnames(win)<-c("chr", "start", "end")
chr_lengths<-read.table("genome_file.txt", header=T)
```

```r
# Filter for full 1 Mb windows:

win %>% mutate(win_length=end-start) %>%
  filter(win_length == 1000000) -> filtered_win

count<-c()
for (i in 1:length(filtered_win$chr)) {
print(filtered_win[i,1])
 dat %>%
  filter(chr == paste(filtered_win[i,1]) & pos >= filtered_win[i,2] & pos <= filtered_win[i,3]) %>%
  dim() -> x
  count[i]<-x[1]
}
filtered_win %>% mutate(count) -> filtered_win

xlabel<-"count per 1Mb window"
ylabel<-"Frequency"
title<-"SNPs per 1Mb window"
filtered_win %>%
  ggplot(aes(count)) +
  geom_histogram(binwidth = 100,colour="#000000", fill="#bdbdbd") +
  theme_classic(base_size = 12) +
  xlab(xlabel)+
  ylab(ylabel)+
ggtitle(title) + ylim(-1,450) + xlim(-100,3500) +
  theme(plot.margin = unit(c(5,5,5,5),"mm"),
        panel.spacing.x = unit(2,"mm"),
        title = element_text(hjust = 0.5),
        legend.position="none",
        axis.title.x=element_text(),
        axis.text.x=element_text(),
        plot.title= element_text(hjust= 0.5),
        axis.title.y=element_text(),
        strip.text.x = element_text(angle=90, margin=unit(c(15,5,15,5),"mm"), size=15),
        strip.background = element_blank())
```

Read-mapping based SV detection

Long-read mapping based SV Detection

As a first step, (long) reads are aligned to a reference assembly using NGM-LR. Resulting bam files are sorted and indexed:

```bash
ngmlr \
   -r reference \
   -q individual.fastq \
   -t 16 \
   -x pacbio \
   | samtools view -Sb - >  individual.bam

samtools sort individual.bam  -o individual.sorted.bam
samtools index individual.sorted.bam
```

Next, these bam files are used to call SVs with `Sniffles`:

```
sniffles \
   -m individual.sorted.bam \
   -v individual.vcf \
   -s 5 \
   --threads 1 \
   --genotype \
   --cluster
```

The `-s 5` option specifies the minimum number of reads supporting a given variant.

Next, the individual vcf files were filtered using `bcftools` and `grep` according to the following criteria: allele frequency (based on supporting reads) > 0.3 SV length < 100 kb (to remove erroneous chromosome-scale variants) Number of supporting reads < 60 (to remove variants caused by high-copy repeats) Remove translocations (again to remove numerous erroneous variants)

```
grep -v '<TRA>' individual.vcf | bcftools view -i 'AF>0.3 && SVLEN<100000 && RE[1]<60'  -  > \
individual_filtered.vcf
```

These individual files were then merged using `SURVIVOR`

```
SURVIVOR \
   merge \
   vcf_list_with_all_single_vcf_files \
   1000 \
   1 \
   1 \
   0 \
   0 \
   50 \
   merged_filtered.vcf
```

Here the filtering parameters were the following: max distance between breakpoints: 1000 Minimum number of supporting caller: 1 Take the type into account (1==yes, else no): 1 Take the strands of SVs into account (1==yes, else no): 0 Estimate distance based on the size of SV (1==yes, else no): 0 Minimum size of SVs to be taken into account: 50

This merged vcf file built from all single vcf files was then used as the basis for another round of SV calling, this time with the `--Ivcf` option which enables the input of a list of SVs which are then genotyped given the bam files. Additionally, we used the `--min_homo_af 0.7` option to improve genotype accuracy (after some initial testing).

```
sniffles \
   -m individual.sorted.bam \
   -v individual_force_called.vcf \
   --not_report_seq \
   --min_homo_af 0.7 \
   --threads 1 \
   --genotype \
   --Ivcf merged_filtered.vcf
```

The individual force-called vcfs were then again merged with `Sniffles`:

```
SURVIVOR \
   merge \
   vcf_list_with_all_single_force_called_vcf_files \
   1000 \
```

```
   1 \
   1 \
   0 \
   0 \
   50 \
   merged_filtered_force_called.vcf
```

Before these variants with the corresponding genotypes per individuals were used for further analysis, we performed further filtering steps. First we removed variants overlapping with gaps (as denoted by 'Ns' in the reference sequence):

```
vcf = merged_filtered_force_called.vcf
bed = Ns_HCv5.5.bed
bedtools intersect -v -a $vcf -b $bed | cat <(grep '#' $vcf) - > temp
```

We then converted this vcf file to a genotype file with `vcftools` for downstream analysis in `R`: The `pop` file is a single-column file with numbers for respective populations / species.

```
vcf_file = temp
output_1 = merged_filtered_force_called.GT_format
output_2 = merged_filtered_force_called.chr_pos_type_len

vcftools --vcf ${vcf_file} --extract-FORMAT-info GT --stdout |\
 tail -n +2 | \
 cut -f 3- | cat -n | \
 python -c \
 "import sys; print('\n'.join(' '.join(c) for c in zip(*(l.split() for l in sys.stdin.readlines() \
 if l.strip()))))" - | \
 sed -e 's/0\/0/0/g' -e 's/0\/1/1/g' -e 's/1\/1/2/g' -e 's/\.\/\./NA/g'  | \
 paste pop -  | tr "\t" " " > $output_1

echo 'chr       pos     type    len' >> $output_2
grep -v '#' $vcf_file | tr ";" "\t" |\
  cut -f 1,2,5,10 | \
   sed -e 's/AVGLEN=//g' -e 's/>//g' -e 's/<//g' \
   -e 's/SVLEN=-//g' -e 's/SVLEN=//g' -e 's/\//_/g'  >> $output_2
```

Downstream analysis of LR variants in `R`

The genotype file was then used in the downstream analysis:

First load the used packages:

```
library(easypackages)
libraries("ggplot2", "plyr", "tidyr", "dplyr", "purrr", "grid",
          "gridExtra", "lme4", "SNPRelate", "gdsfmt")
`%not_in%` <- purrr::negate(`%in%`)
```

Then load the genotype, variant and chromosome length file:

```
dat<-read.table("merged_filtered_force_called.GT_format", header=T)
loc<-read.table("merged_filtered_force_called.chr_pos_type_len", header=T)
chr_lengths<-read.table("v5.5.genome_file.txt", header=T)
colnames(chr_lengths)<-c("id", "length")
```

PCA

Next, perform the PCA with the LR genotypes:

```
backup<-dat
genos<-dat[,2:length(dat[1,])]
samples <- read.table("data_pops", header=FALSE)
names(samples)<-c("pop", "pop_nr", "sampleID")

closefn.gds(SVs)
genotypes <- as.matrix(genos)
sample.ids <- as.character(samples$sampleID)
SV.ids <- paste("SV",1:ncol(genotypes),sep="")

snpgdsCreateGeno("SVs.gds", genmat=genotypes, sample.id=sample.ids,
                 snp.id=SV.ids, snpfirstdim=FALSE)
SVs <- openfn.gds("SVs.gds", readonly=FALSE)

pca <- snpgdsPCA(SVs, snp.id=SV.ids, num.thread=7, sample.id=sample.ids)
pca_perc<-pca$varprop*100

pop <- c("HC_genome_ind", "HC", "HC", "HC", "HC", "HC", "HC", "HC", "HC",
         "CC", "CC", "CC", "CC", "CC", "CC", "CC", "CC", "CCE", "CCE", "CCE", "CCE", "CCE", "CCE",
         "AC", "AC", "JD", "JD" , "JD", "JD", "JD", "DJD", "DJD", "DJD")
pca$eigenvect %>% data.frame() %>% select(X1, X2, X3, X4, X5, X6) -> pca_eigenvectors
names(pca_eigenvectors) <- c("PC1", "PC2", "PC3", "PC4", "PC5", "PC6")
pca_eigenvectors <- cbind(pop, pca_eigenvectors)

col<-c("red", "blue", "green", "orange", "black", "brown")
values<-c("DJD", "AC", "CCE", "JD", "CC", "HC")
names(col)<- values
pca_eigenvectors %>% filter(pop != "HC_genome_ind") %>%
     ggplot(aes(PC1, PC2, colour=pop)) +
     geom_point(size = 2)  + scale_colour_manual(values=col, guide= FALSE) +
     theme_classic(base_size = 9) +
     xlab(paste0("PC 1( ", round(pca_perc[1], digits=3), " %)")) +
     ylab(paste0("PC 2( ", round(pca_perc[2], digits=3), " %)")) -> pca_all
```

Now repeat PCA for the respective clades (crow and jackdaw) and for the European crow populations and plot all in one figure:

```
dat<-backup
genos_crow_clade<-dat[1:25,2:length(dat[1,])]
samples <- read.table("data_pops", header=FALSE)
names(samples)<-c("pop", "pop_nr", "sampleID")
samples_crow_clade<-samples[1:25,]

closefn.gds(SVs)
genotypes <- as.matrix(genos_crow_clade)
sample.ids <- as.character(samples_crow_clade$sampleID)
SV.ids <- paste("SV",1:ncol(genotypes),sep="")

snpgdsCreateGeno("SVs.gds", genmat=genotypes, sample.id=sample.ids, snp.id=SV.ids, snpfirstdim=FALSE)
SVs <- openfn.gds("SVs.gds", readonly=FALSE)
```

```
pca <- snpgdsPCA(SVs, snp.id=SV.ids, num.thread=7, sample.id=sample.ids)
pca_perc<-pca$varprop*100

pop <- c("HC_genome_ind", "HC", "HC", "HC", "HC", "HC", "HC", "HC", "HC",
         "CC", "CC", "CC", "CC", "CC", "CC", "CC", "CC", "CCE", "CCE", "CCE", "CCE", "CCE", "CCE",
         "AC", "AC", "JD", "JD" , "JD", "JD", "JD", "DJD", "DJD", "DJD")
pop<-pop[1:25]
pca$eigenvect %>% data.frame() %>% select(X1, X2, X3, X4, X5, X6) -> pca_eigenvectors
names(pca_eigenvectors) <- c("PC1", "PC2", "PC3", "PC4", "PC5", "PC6")
pca_eigenvectors <- cbind(pop, pca_eigenvectors)

col<-c("red", "blue", "green", "orange", "black", "brown")
values<-c("DJD", "AC", "CCE", "JD", "CC", "HC")
names(col)<- values
pca_eigenvectors %>% filter(pop != "HC_genome_ind") %>%
  ggplot(aes(PC1, PC2, colour=pop)) +
  geom_point(size = 2)  + scale_colour_manual(values=col, guide= FALSE) +
    theme_classic(base_size = 9) +
    xlab(paste0("PC 1( ", round(pca_perc[1], digits=3), " %)")) +
    ylab(paste0("PC 2( ", round(pca_perc[2], digits=3), " %)")) -> pca_crow_clade

dat<-backup
genos_euro_crow_clade<-dat[1:23,2:length(dat[1,])]
samples <- read.table("data_pops", header=FALSE)
names(samples)<-c("pop", "pop_nr", "sampleID")
samples_euro_crow_clade<-samples[1:23,]

closefn.gds(SVs)
genotypes <- as.matrix(genos_euro_crow_clade)
sample.ids <- as.character(samples_euro_crow_clade$sampleID)
SV.ids <- paste("SV",1:ncol(genotypes),sep="")

snpgdsCreateGeno("SVs.gds", genmat=genotypes, sample.id=sample.ids, snp.id=SV.ids, snpfirstdim=FALSE)
SVs <- openfn.gds("SVs.gds", readonly=FALSE)

pca <- snpgdsPCA(SVs, snp.id=SV.ids, num.thread=7, sample.id=sample.ids)
pca_perc<-pca$varprop*100

pop <- c("HC_genome_ind", "HC", "HC", "HC", "HC", "HC", "HC", "HC", "HC",
         "CC", "CC", "CC", "CC", "CC", "CC", "CC", "CC", "CCE", "CCE", "CCE", "CCE", "CCE", "CCE",
         "AC", "AC", "JD", "JD" , "JD", "JD", "JD", "DJD", "DJD", "DJD")
pop<-pop[1:23]
pca$eigenvect %>% data.frame() %>% select(X1, X2, X3, X4, X5, X6) -> pca_eigenvectors
names(pca_eigenvectors) <- c("PC1", "PC2", "PC3", "PC4", "PC5", "PC6")
pca_eigenvectors <- cbind(pop, pca_eigenvectors)

col<-c("red", "blue", "green", "orange", "black", "brown")
values<-c("DJD", "AC", "CCE", "JD", "CC", "HC")
names(col)<- values
pca_eigenvectors %>% filter(pop != "HC_genome_ind") %>%
    ggplot(aes(PC1, PC2, colour=pop)) + geom_point(size = 2)  +
    scale_colour_manual(values=col, guide= FALSE) +
    theme_classic(base_size = 9) +
```

```
    xlab(paste0("PC 1( ", round(pca_perc[1], digits=3), " %)")) +
    ylab(paste0("PC 2( ", round(pca_perc[2], digits=3), " %)")) -> pca_euro_crow_clade

svg("pca_LR.svg", height=2, width=7)
grid.arrange(pca_all, pca_crow_clade, pca_euro_crow_clade, ncol=3)
dev.off()
```

'Phylogenetic filtering'

Next, we performed the 'phylogenetically-informed' filtering.

```
# Transpose the genotype table to get one individual per line and one variant per column
t_dat <- t(dat[,2:ncol(dat)])
t_dat %>% group_by(chr,pos, type, len) %>%
  gather(individual, genotype, 9:41) %>%
  data.frame() -> table
table$individual<-factor(table$individual, levels=c(1:33))

# set individuals of species and populations
# hooded crow
hc<-c(2:9)
# German carrion crow
cc<-c(10:17)
# Spanish carrion crow
cce<-c(18:23)
# American crow
ac<-c(24:25)
# jackdaw
jd<-c(26:30)
# Daurian jackdaw

djd<-c(31:33)
all<-c(1:33)
crows<-c(2:25)
jd_clade<-c(26:33)

colors<-c("#33a02c", "#b2df8a", "#1f78b4", "#a6cee3")
n<-c(0,1,2, "genotype_NA")
names(colors)<-n

# use the chr_lengths file to sort chromosomes by size
chr_lengths<-chr_lengths[order(-chr_lengths$length),]
  chr_lengths
  include_chr<-chr_lengths$id[chr_lengths$length>0] # this is arbitrary and may be changed
  include_chr
  filtered_table<-table[table$chr %in% include_chr,]
  filtered_table$chr<-factor(filtered_table$chr, levels=include_chr)
  head(filtered_table)

# Introduce a unique identifier based on chromosome and position for each variant:
filtered_table %>%
  mutate(SV_ID=paste0(chr,"_",pos)) %>%
  mutate(new_genotype = genotype) -> temp
```

```r
filtered_table <- temp

# Add a column to assess which variants have genotype `NA`
filtered_table$new_genotype[which(is.na(filtered_table$new_genotype))] <- "genotype_NA"

# Exclude the genome individual

filtered_table %>% filter(individual !=1) -> temp
filtered_table <- temp

# Sites which have two variants at the same position starting
filtered_table %>% filter(SV_ID %not_in% exclude_variants) -> temp
filtered_table <- temp

# Filter duplicated sites:

filtered_table %>% filter(individual == 2) %>% select(SV_ID) %>%
  mutate(dup = duplicated(SV_ID)) %>% filter(dup == "TRUE") %>% select(SV_ID) %>%
  unlist() %>% as.vector() -> duplicated_variants

filtered_table %>% filter(SV_ID %not_in% duplicated_variants) -> temp
filtered_table <- temp

# Exclude variants which are not insertions, deletions or inversions:
filtered_table %>% filter(type != "DUP") %>%
  filter(type != "DUP_INS") %>%
  filter(type != "INV_INVDUP") %>%
  filter(type != "INVDUP")-> temp
filtered_table <- temp

# Now look which variants are all homozygous variant or reference
# in the jackdaw clade, allowing for 2 errors:
filtered_table %>%
  filter(individual %in% jd_clade) %>%
  group_by(SV_ID, chr, pos, type, len) %>%
  summarise(homo_ref=sum(genotype==0, na.rm=TRUE),
            het=sum(genotype==1, na.rm=TRUE),
            homo_var=sum(genotype==2, na.rm=TRUE)) %>%
 mutate(enough_data=ifelse(sum(c(homo_ref,het,homo_var))>=6, "yes","no")) %>%
 filter(enough_data == "yes") %>% filter(homo_var >=6) %>%
 ungroup() %>% select(SV_ID) %>% unlist() %>% as.vector() -> homo_var_jd_clade

filtered_table %>%
  filter(individual %in% jd_clade) %>%
  group_by(SV_ID, chr, pos, type, len) %>%
  summarise(homo_ref=sum(genotype==0, na.rm=TRUE),
            het=sum(genotype==1, na.rm=TRUE),
            homo_var=sum(genotype==2, na.rm=TRUE)) %>%
 mutate(enough_data=ifelse(sum(c(homo_ref,het,homo_var))>=6, "yes","no")) %>%
 filter(enough_data == "yes") %>% filter(homo_ref >=6) %>%
 ungroup() %>% select(SV_ID) %>% unlist() %>% as.vector() -> homo_ref_jd_clade

# Now the same for the crow clade, allowing for 3 errors:
```

```
  filtered_table %>%
    filter(individual %in% crows) %>%
    group_by(SV_ID, chr, pos, type, len) %>%
    summarise(homo_ref=sum(genotype==0, na.rm=TRUE),
              het=sum(genotype==1, na.rm=TRUE),
              homo_var=sum(genotype==2, na.rm=TRUE)) %>%
    mutate(enough_data=ifelse(sum(c(homo_ref,het,homo_var))>=21, "yes","no")) %>%
    filter(enough_data == "yes") %>% filter(homo_ref >=21) %>%
    ungroup() %>% select(SV_ID) %>% unlist() %>% as.vector() -> homo_ref_crow_clade

filtered_table %>% filter(SV_ID %in% homo_ref_crow_clade ) %>% filter(individual == 2) %>%
  select(SV_ID) %>% unlist() %>% as.vector() -> jd_clade_variants

filtered_table %>% filter(SV_ID %in% homo_var_jd_clade | SV_ID %in% homo_ref_jd_clade) %>%
  filter(individual == 2) %>%
  select(SV_ID) %>% unlist() %>% as.vector() -> crow_clade_variants

filtered_table %>% filter(SV_ID %in% crow_clade_variants | SV_ID %in% jd_clade_variants) %>%
  filter(individual == 2) %>%
  select(SV_ID) %>% unlist() %>% as.vector() -> phylo_filtered_variants

filtered_table %>% filter(SV_ID %not_in% phylo_filtered_variants) %>% filter(individual == 2) %>%
  select(SV_ID) %>% unlist() %>% as.vector() -> polymorphic_across_clades
```

In the next step, we determined whether variant type, distance to chromosome end and repeat density are associated with removed variants:

```
# Determine distance to chromosome end:

# first use one individual in the table :

filtered_table %>% filter(individual == 2) -> input

# Load a bed file with 1 Mb windows
win<-read.table("1Mb_windows.genome.HCv5.5.bed", header=F)
colnames(win)<-c("chr", "start", "end")
chr_lengths<-read.table("HCv5.5.genome_file.txt", header=T)

# Load RepeatMasker bed file
repeat_masker_track<-read.table("repeatmasker_HCv5.5.bed", header=F)
names(repeat_masker_track)<-c("chr", "start", "end", "ID", "family")

# Run a loop to get repeat density per window
rep_density<-c()
for (i in 1:length(win$chr)){
  repeat_masker_track %>% filter(chr==paste0(win[i,1]) &
                                   start >= win[i,2] &
                                   end <= win[i,3]) %>% dim() -> temp
  rep_density[i]<-temp[1]
}
win<-cbind(win, rep_density)
distance_to_chr_end<-c()
  for (i in 1:length(win$chr)){
    print(win$chr[i] )
```

```
    print(win$start[i])
    if (win$start[i] < chr_lengths %>% filter(ID == paste(win$chr[i])) %>%
        select(length) %>% unlist() %>% as.vector() / 2){
    distance_to_chr_end[i]<-win$start[i]
    }
    else{
      distance_to_chr_end[i]<- chr_lengths %>%
        filter(ID == paste(win$chr[i])) %>%
        select(length) %>% unlist() %>% as.vector() - win$start[i]
    }
  }
win <- cbind(win, distance_to_chr_end)
win %>% mutate(distance_to_chr_end_Mb = distance_to_chr_end / 1000000) -> temp
win <- temp

# Plot repeat density vs. relative distance to chromosome end
win %>%mutate(ID=chr) %>%
  merge(., chr_lengths, by="ID") %>%
  mutate(rel_dist_to_chr_end=distance_to_chr_end/(length/2)) %>%
  ggplot(aes(rel_dist_to_chr_end, rep_density)) +
   theme_classic(base_size = 9) +
   geom_point() +
   geom_smooth() +
   xlab("Relative distance to chromosome end") +
   ylab("Repeat density per 1-Mb window")  -> distance_to_chr_end_vs_rep_density

# Define a function to get the repeat density per site
get_repeats<-function(chrID,posID){
 tem_table<-win %>%
   filter(chr == chrID & as.numeric(start) <= as.numeric(posID) &
            as.numeric(end) >= as.numeric(posID))
 if(dim(tem_table)[1]!=0){
    return(tem_table$rep_density)
  } else {
    return(0)
  }
}

# Run over all chromosomes
rep_density_per_site<-c()
for (i in 1:length(input$chr)){
  print(input$pos[i])
  if (input$pos[i] < chr_lengths %>%
      filter(ID == paste(input$chr[i])) %>%
      select(length) %>% unlist() %>% as.vector() / 2){
  distance_to_chr_end[i]<-input$pos[i]
  }
  else{
    distance_to_chr_end[i]<- chr_lengths %>%
      filter(ID == paste(input$chr[i])) %>%
      select(length) %>% unlist() %>% as.vector() - input$pos[i]
  }
 rep_density_per_site[i]<- get_repeats(chrID = paste0(input$chr[i]),
```

```
                                              posID = as.numeric(input$pos[i]))
}

# Split in two tables, one for kept and one for removed variants
input <- cbind(input, rep_density_per_site)
input %>% mutate(distance_to_chr_end)  %>%
  filter(SV_ID %in% polymorphic_across_clades ) -> GTs_filtered_out
input %>% mutate(distance_to_chr_end) %>%
  filter(SV_ID %not_in%  polymorphic_across_clades) -> GTs_kept

GTs_kept$keptYN <- 1
GTs_filtered_out$keptYN <- 0
dat_GT <- rbind(GTs_kept,GTs_filtered_out)
dat_GT$DistanceToEndMB <- dat_GT$distance_to_chr_end / 1000000
dat_GT$lenMB <- dat_GT$len / 1000000

# Run Linear models to assess the influence of Distance to
# chromosome end, variant type and repeat density on the variant filter
mod1 <- glm(keptYN ~ DistanceToEndMB + factor(type), data=dat_GT, family=binomial)
summary(mod1)

mod1 <- glm(keptYN ~ rep_density_per_site, data=dat_GT, family=binomial)
summary(mod1)

mod2 <- glm(keptYN ~ DistanceToEndMB + factor(type), data=dat_GT, family=quasibinomial)
summary(mod1)
summary(mod2)
mod3 <- glmer(keptYN ~ scale(DistanceToEndMB) + factor(type) + (1|chr), data=dat_GT, family=binomial)
  summary(mod3)

# Produce figures

colors<-c("red", "black")
n<-c(0,1)
names(colors)<-n

dat_GT %>% filter(type != "DUP") %>%
  ggplot(aes(type)) +
  geom_bar(aes(fill=as.factor(keptYN))) +
  ylab("Count") +
  xlab("SV Class") +
  scale_fill_manual(breaks=c("0", "1"),
                    values=c("red", "black"),
                    labels=c("Genotypes filtered",
                             "Genotypes kept"), name="" ) +
  theme_classic(base_size=9) +
  theme( legend.justification=c(1,1),
         legend.position="none") -> phylo_filter_variant_class

dat_GT %>%  mutate(ID=chr) %>%
  merge(., chr_lengths, by="ID") %>%
  mutate(rel_dist_to_chr_end=distance_to_chr_end/(length/2)) %>%
ggplot(aes(rel_dist_to_chr_end)) +
```

```
  theme_classic(base_size = 9) +
   geom_density(aes(group=keptYN, colour = as.factor(keptYN)) ) +
   scale_colour_manual( breaks=c("0", "1"),
                        values=c("red", "black"),
                        labels=c("Variants filtered", "
                                 Variants kept"), name="") +
   ylab("Density") + xlab("Relative distance to chromosome end") +
   theme(legend.justification=c(1,1),
         legend.position="none") -> phylo_filter_density_plot


pdf("Figure_2B.pdf", width = 26, height = 8)
svg("Figure_2B_2019-09-30.svg", width=8, height=2.5)
grid.arrange(phylo_filter_variant_class,
             phylo_filter_density_plot,
             distance_to_chr_end_vs_rep_density, ncol=3)
dev.off()
```

SV lengths by LR and OM

Here we plotted the lengths of SVs identified with LR and OM:

```
dat<-read.table("OM_LR_SVs_2019-09-16", header=F)
names(dat) <- c("chr", "pos", "source", "len", "type")
dat %>% filter(type %in% c("INS", "DEL", "INV")) -> temp
dat <- temp
summary(dat)

xlabel<-"Length (bp)"
ylabel<-"Count"
filtered_table %>% filter(individual == 2) %>%
  filter(type != "INV")  %>%
  filter(SV_ID %not_in% polymorphic_across_clades) %>%
  select(len, type) %>%
  mutate(source="LR") ->  lr_svlen_table
dat %>% filter(type != "INV") %>%
  filter(source %in% c("OM", "OM_and_LR")) %>%
  filter(len > 1000) %>%
  select(len, type) %>%
  mutate(source="OM") %>% rbind(., lr_svlen_table) %>%
  ggplot(aes(len, fill=type, color=type)) +
  geom_histogram(binwidth = 20) +
  xlab(xlabel)+
  ylab(ylabel) +
  xlim(0,10000) + theme_classic(base_size= 9) + scale_y_log10() +
  facet_grid( rows = vars(source), scales="free_y") +
  theme(strip.background = element_blank(), legend.position = "none",
        axis.text.y=element_text(size = 9, colour="black"),
        axis.text.x=element_text(size = 9, colour="black")) -> LR_OM_readlength

svg("Supplementary_Figure_INV_length_dist.svg" , width=3.5, height=3.5)
xlabel<-"Length (bp)"
ylabel<-"Count"
```

```
filtered_table %>%
  filter(individual == 2) %>%
  filter(type == "INV")  %>%
  filter(SV_ID %not_in% polymorphic_across_clades) %>%
  select(len, type) %>% mutate(source="LR") ->  lr_svlen_table
dat %>% filter(type == "INV") %>%
  filter(source %in% c("OM", "OM_and_LR")) %>%
  filter(len > 1000) %>% select(len, type) %>%
  mutate(source="OM") %>% rbind(., lr_svlen_table) %>%
  ggplot(aes(len, fill=type, color=type)) + geom_histogram(binwidth = 20) +
  xlab(xlabel)+ ylab(ylabel) + xlim(0,10000) + theme_classic(base_size= 9) +
  facet_grid( rows = vars(source), scales="free_y") +
  theme(strip.background = element_blank(),
        legend.position = "none",
        axis.text.y=element_text(size = 9, colour="black"),
        axis.text.x=element_text(size = 9, colour="black"))
dev.off()
```

Repeat characterization of LR insertions and deletions:

```
rm_output<-read.table("rm_pipeline_output_updated_with_repIDs_correctIDs")
colnames(rm_output) <- c("SV_ID", "repID", "repeat_class")
rm_output %>% filter(SV_ID %not_in% exclude_variants) -> temp
rm_output <- temp

filtered_table %>% filter(individual == 2) %>% filter(type != "INV")   %>% select(SV_ID) %>%
  filter(SV_ID %in% crow_clade_variants | SV_ID %in% jd_clade_variants) %>%
  merge(., rm_output, by="SV_ID", all=TRUE) %>%
  mutate(repeat_class=factor(ifelse(is.na(repeat_class),
                                    "no_hit",as.vector(repeat_class))))-> rm_output

rm_output %>% filter(SV_ID %in% crow_clade_variants |
                       SV_ID %in% jd_clade_variants) -> phylofiltered_rm_output

plyr::count(phylofiltered_rm_output$repeat_class) %>%
  mutate(repeat_class=factor(ifelse(freq < 700,"Other",as.vector(x)))) %>%
  arrange(desc(freq)) -> z_filtered

col<-c("#1f78b4","#ff7f00", "#fdbf6f", "#e31a1c","#33a02c",
       "#b2df8a","#b2df8a", "#b2df8a", "#b2df8a","#b2df8a",
       "#b2df8a","#b2df8a","#b2df8a")
n<-c("no_hit","LTR", "Simple_repeat" , "Low_complexity",
     "LINE/CR1","Other","Other","Other",
     "Other","Other","Other" ,"Other" ,"Other")
names(col)<-n

z_filtered %>%
  filter(repeat_class == "Other") %>%
  select(freq) %>%
  sum() -> other_sum
z_filtered %>%
  filter(repeat_class!="Other") %>%
```

```
  add_row(., x="Other", freq=other_sum, repeat_class="Other") -> temp
rep_type<-c("none", "ir", "tr", "tr", "ir", "tr", "ir", "unknown", "tr", "tr", "ir", "tr", "tr")

ggplot(temp, aes(x="", y=freq, fill=repeat_class)) +
 theme_classic(base_size = 9) +
 geom_bar(width = 1, stat = "identity") +
 coord_polar("y", start=90) +
 geom_text(aes(y = freq/6 + c(0, cumsum(freq)[-length(freq)]),
 label = freq), size=3) + theme(legend.position = "bottom",
 axis.title.x=element_blank(),
 axis.text.x=element_blank()) + scale_fill_manual(values=col) -> rm_output_figure

 svg("figure_3ab_2019-10-17.svg", width= 7, height=3)
 grid.arrange(LR_OM_readlength, rm_output_figure, ncol=2)
 dev.off()

 # Look which individual repeat motifs are most common:

# load lengths of individual repeat motifs
rep_lengths<-read.table("rep_lengths", header= F)
names(rep_lengths)<- c("repID", "length")
head(rep_lengths)

# determine frequency of single repeat motifs
phylofiltered_rm_output %>% select(repID) %>%
plyr::count() %>% arrange(desc(freq)) %>%
merge(., phylofiltered_rm_output, by="repID", all = T) %>%
  merge(., rep_lengths, by="repID", all = T) %>%
  arrange(desc(freq)) %>%
filter(repeat_class != "no_hit") %>%
  select(repID, freq, repeat_class, length) %>%
unique() %>%  filter(freq > 100)   %>%
  write.table(., file="freq_over_100.txt", sep ="\t", quote=FALSE, row.names = FALSE)

phylofiltered_rm_output %>% select(repID) %>%
plyr::count() %>% arrange(desc(freq)) %>%
merge(., phylofiltered_rm_output, by="repID", all = T) %>%
  merge(., rep_lengths, by="repID", all = T) %>%  arrange(desc(freq)) %>%
filter(repeat_class != "no_hit") %>%  select(repID, freq, repeat_class, length) %>%
unique() %>%  head(5) %>% select(freq) %>% sum() / 21158
```

Site frequency spectrum of LR variants

```
four_most_common_repeat_classes<-c("no_hit", "LTR", "Simple_repeat", "Low_complexity", "LINE/CR1")

# SFS plots for binned populations - Crow clade

col<-c("#1f78b4","#ff7f00", "#fdbf6f", "#e31a1c","#33a02c" ,
       "#b2df8a","#b2df8a", "#b2df8a", "#b2df8a","#b2df8a","#b2df8a","#b2df8a","#b2df8a")
n<-c("no_hit","LTR", "Simple_repeat" , "Low_complexity" ,
     "LINE/CR1","Other","Other","Other","Other","Other","Other" ,"Other" ,"Other")
names(col)<-n
```

```
filtered_table %>% filter(type != "INV") %>%  filter(SV_ID %in% crow_clade_variants) %>%
  filter(individual %in% c(hc, cc, cce, ac)) %>%
  group_by(SV_ID) %>% summarize(freq=sum(genotype)) %>%
  filter(freq!=0 & freq!=48) %>% mutate(folded_freq=ifelse(freq>=25,48-freq,freq)) %>%
  join(., rm_output, by="SV_ID") %>% filter(repeat_class %in% four_most_common_repeat_classes) %>%
  ggplot(aes(folded_freq, stat(density), fill=repeat_class)) + geom_histogram(binwidth=2) +
  theme_classic(base_size = 9) +
  ylab("Frequency")+
  scale_fill_manual(values = col) +
  theme(plot.margin = unit(c(5,5,5,5),"mm"),
        panel.spacing.x = unit(2,"mm"),
        title = element_text(),
        legend.position="none",
        axis.title.x=element_blank(),
        axis.text.x=element_text(angle=45, vjust=0.5),
        axis.title.y=element_text(),
        strip.text.x = element_text( margin=unit(c(2,2,2,2),"mm")),
        strip.background = element_blank())+
  facet_grid(. ~ repeat_class,
             scale="free_x",
             space="free_x") -> plot
ggplot_build(plot) -> pg

col<-c("#1f78b4","#ff7f00", "#fdbf6f", "#e31a1c","#33a02c" , "#b2df8a","#b2df8a",
       "#b2df8a", "#b2df8a","#b2df8a","#b2df8a","#b2df8a","#b2df8a")
n<-c("#1f78b4","#ff7f00", "#fdbf6f", "#e31a1c","#33a02c" , "#b2df8a","#b2df8a",
     "#b2df8a", "#b2df8a","#b2df8a","#b2df8a","#b2df8a","#b2df8a")
names(col)<-n
titles<-c("LINE/CR1", "Low complexity", "LTR", "No match", "Simple repeat")
n<-c(1,2,3,4,5)
names(titles)<-n

pg$data[[1]] %>% mutate(chr_nr = x+0.5) -> temp
temp$group<-factor(temp$group,levels= c(1,3,2,5,4))

labels<-c("0.04", "", "0.12", "", "0.21", "", "0.29", "", "0.38", "", "0.46", "")
temp %>%
  ggplot(aes(as.factor(chr_nr), y=density, fill=fill)) + geom_bar(stat= "identity") +
  theme_classic(base_size = 9) +
  ylab("Frequency")+
  scale_x_discrete(labels=labels)+
  scale_fill_manual(values = col) +
  theme(plot.margin = unit(c(5,5,5,5),"mm"),
        panel.spacing.x = unit(2,"mm"),
        title = element_text(),
        legend.position="none",
        axis.title.x=element_blank(),
        axis.text.x=element_text( angle=75, vjust=0.5),
        axis.title.y=element_text(),
        strip.text.x = element_text(size=9, margin=unit(c(2,2,2,2),"mm")),
        strip.background = element_blank())+
  facet_grid(. ~ group,
             labeller=labeller(group = titles),
```

```
            scale="free_x",
            space="free_x") ->sfs_crow_clade_indels

filtered_table %>% filter(type == "INV") %>%
  filter(SV_ID %in% crow_clade_variants) %>%
  filter(individual %in% c(hc, cc, cce, ac)) %>%
  group_by(SV_ID) %>% summarize(freq=sum(genotype)) %>%
  filter(freq!=0 & freq!=48) %>%
  mutate(folded_freq=ifelse(freq>=25,48-freq,freq)) %>%
    ggplot(aes(folded_freq, stat(density))) + geom_histogram(binwidth=2) +
    theme_classic(base_size = 9) +
        ylab("Frequency")+
      theme(plot.margin = unit(c(5,5,5,5),"mm"),
        panel.spacing.x = unit(2,"mm"),
        title = element_text(),
        legend.position="none",
        axis.title.x=element_blank(),
        axis.text.x=element_text(angle=45),
        axis.title.y=element_text(),
        strip.text.x = element_text( margin=unit(c(2,2,2,2),"mm")),
          strip.background = element_blank()) -> plot
      ggplot_build(plot) -> pg

pg$data[[1]] %>% mutate(chr_nr = x+0.5) -> temp

temp %>%
ggplot(aes(as.factor(chr_nr), y=density)) + geom_bar(stat= "identity") +
    theme_classic(base_size = 9) +
        scale_x_discrete(labels=labels)+
        ggtitle("Inversion") +
      theme(plot.margin = unit(c(5,5,5,2),"mm"),
        panel.spacing.x = unit(2,"mm"),
        plot.title = element_text( size=9,hjust=0.5),
        legend.position="none",
        axis.title.x=element_blank(),
        axis.text.x=element_text( angle=75, vjust=0.5),
        axis.title.y=element_blank(),
        strip.text.x = element_text( margin=unit(c(2,2,2,2),"mm")),
          strip.background = element_blank())  -> sfs_crow_clade_inversion

svg("sfs_crow_clade_binned.svg",  width= 7, height=3)
grid.arrange(sfs_crow_clade_indels, sfs_crow_clade_inversion, ncol=2, widths=c(5,1.3))
dev.off()

# SFS plots for binned populations - Jackdaw clade

col<-c("#1f78b4","#ff7f00", "#fdbf6f", "#e31a1c","#33a02c" , "#b2df8a",
       "#b2df8a", "#b2df8a", "#b2df8a","#b2df8a","#b2df8a","#b2df8a","#b2df8a")
n<-c("no_hit","LTR", "Simple_repeat" , "Low_complexity" ,"LINE/CR1",
     "Other","Other","Other","Other","Other","Other" ,"Other" ,"Other")
names(col)<-n

filtered_table %>% filter(type != "INV") %>%
```

```
  filter(SV_ID %in% jd_clade_variants) %>%
  filter(individual %in% jd_clade) %>%
  group_by(SV_ID) %>% summarize(freq=sum(genotype)) %>%
  filter(freq!=0 & freq!=16) %>% mutate(folded_freq=ifelse(freq>=9,16-freq,freq)) %>%
  join(., rm_output, by="SV_ID") %>%
  filter(repeat_class %in% four_most_common_repeat_classes) %>%
  ggplot(aes(folded_freq, stat(density), fill=repeat_class)) +
  geom_histogram(binwidth=2) +
  theme_classic(base_size = 9) +
  ylab("Frequency")+
  scale_fill_manual(values = col) +
  theme(plot.margin = unit(c(5,5,5,5),"mm"),
        panel.spacing.x = unit(2,"mm"),
        title = element_text(),
        legend.position="none",
        axis.title.x=element_blank(),
        axis.text.x=element_text(angle=45, vjust=0.5),
        axis.title.y=element_text(),
        strip.text.x = element_text( margin=unit(c(2,2,2,2),"mm")),
        strip.background = element_blank())+
  facet_grid(. ~ repeat_class,
             scale="free_x",
             space="free_x") -> plot
ggplot_build(plot) -> pg

col<-c("#1f78b4","#ff7f00", "#fdbf6f", "#e31a1c","#33a02c" , "#b2df8a",
       "#b2df8a", "#b2df8a", "#b2df8a","#b2df8a","#b2df8a","#b2df8a","#b2df8a")
n<-c("#1f78b4","#ff7f00", "#fdbf6f", "#e31a1c","#33a02c" , "#b2df8a",
     "#b2df8a", "#b2df8a", "#b2df8a","#b2df8a","#b2df8a","#b2df8a","#b2df8a")
names(col)<-n
titles<-c("LINE/CR1", "Low complexity", "LTR", "No match", "Simple repeat")
n<-c(1,2,3,4,5)
names(titles)<-n

pg$data[[1]] %>% mutate(chr_nr = x+0.5) -> temp
temp$group<-factor(temp$group,levels= c(1,3,2,5,4))

temp %>%
  ggplot(aes(as.factor(chr_nr), y=density, fill=fill)) +
  geom_bar(stat= "identity") +
  theme_classic(base_size = 9) +
  ylab("Frequency")+
  scale_x_discrete(labels=round(seq(2,8,2)/16, digits=2))+
  scale_fill_manual(values = col) +
  theme(plot.margin = unit(c(5,5,5,5),"mm"),
        panel.spacing.x = unit(2,"mm"),
        title = element_text(),
        legend.position="none",
        axis.title.x=element_blank(),
        axis.text.x=element_text(size=9, angle=75, vjust=0.5),
        axis.title.y=element_text(),
        strip.text.x = element_text( margin=unit(c(2,2,2,2),"mm")),
        strip.background = element_blank())+
```

```
  facet_grid(. ~ group,
             labeller=labeller(group = titles),
             scale="free_x",
             space="free_x") ->sfs_jackdaw_clade_indels

filtered_table %>% filter(type == "INV") %>%
  filter(SV_ID %in% jd_clade_variants) %>%
  filter(individual %in% jd_clade) %>%
  group_by(SV_ID) %>% summarize(freq=sum(genotype)) %>%
  filter(freq!=0 & freq!=16) %>%
  mutate(folded_freq=ifelse(freq>=9,16-freq,freq)) %>%
  ggplot(aes(folded_freq, stat(density))) + geom_histogram(binwidth=2) +
  theme_classic(base_size = 9) +
  ylab("Frequency")+
  theme(plot.margin = unit(c(5,5,5,5),"mm"),
        panel.spacing.x = unit(2,"mm"),
        title = element_text(),
        legend.position="none",
        axis.title.x=element_blank(),
        axis.text.x=element_text(angle=45),
        axis.title.y=element_text(),
        strip.text.x = element_text( margin=unit(c(2,2,2,2),"mm")),
        strip.background = element_blank()) -> plot
ggplot_build(plot) -> pg

pg$data[[1]] %>% mutate(chr_nr = x+0.5) -> temp

temp %>%
  ggplot(aes(as.factor(chr_nr), y=density)) + geom_bar(stat= "identity") +
  theme_classic(base_size = 9) +
  scale_x_discrete(labels=round(seq(2,8,2)/16, digits=2))+
  ggtitle("Inversion") +
  theme(plot.margin = unit(c(5,5,5,2),"mm"),
        panel.spacing.x = unit(2,"mm"),
        plot.title = element_text(size=9, hjust=0.5),
        legend.position="none",
        axis.title.x=element_blank(),
        axis.text.x=element_text(size=9, angle=75, vjust=0.5),
        axis.title.y=element_blank(),
        strip.text.x = element_text( margin=unit(c(2,2,2,2),"mm")),
        strip.background = element_blank())  -> sfs_jackdaw_clade_inversion

svg("sfs_jd_clade_binned.svg",  width= 7, height=3)
grid.arrange(sfs_jackdaw_clade_indels,
             sfs_jackdaw_clade_inversion, ncol=2, widths=c(5,1))
dev.off()

# SFS for single populations:

# HC SFS
### SFS plots for binned populations:

col<-c("#1f78b4","#ff7f00", "#fdbf6f", "#e31a1c","#33a02c" ,
```

```
       "#b2df8a","#b2df8a", "#b2df8a", "#b2df8a","#b2df8a","#b2df8a","#b2df8a","#b2df8a")
n<-c("no_hit","LTR", "Simple_repeat" , "Low_complexity" ,"
     LINE/CR1","Other","Other","Other","Other","Other","Other" ,"Other" ,"Other")
names(col)<-n

filtered_table %>% filter(type != "INV") %>%
  filter(SV_ID %in% crow_clade_variants) %>%
  filter(individual %in% hc) %>%
  group_by(SV_ID) %>% summarize(freq=sum(genotype)) %>%
  filter(freq!=0 & freq!=16) %>%
  mutate(folded_freq=ifelse(freq>=9,16-freq,freq)) %>%
  join(., rm_output, by="SV_ID") %>% filter(repeat_class %in% four_most_common_repeat_classes) %>%
  ggplot(aes(folded_freq, stat(density), fill=repeat_class)) +
  geom_histogram(binwidth=2) +
  theme_classic(base_size = 9) +
  ylab("Frequency")+
  scale_fill_manual(values = col) +
  theme(plot.margin = unit(c(5,5,5,5),"mm"),
        panel.spacing.x = unit(2,"mm"),
        title = element_text(),
        legend.position="none",
        axis.title.x=element_blank(),
        axis.text.x=element_text(angle=45, vjust=0.5),
        axis.title.y=element_text(),
        strip.text.x = element_text( margin=unit(c(2,2,2,2),"mm")),
        strip.background = element_blank())+
  facet_grid(. ~ repeat_class,
             scale="free_x",
             space="free_x") -> plot
ggplot_build(plot) -> pg

col<-c("#1f78b4","#ff7f00", "#fdbf6f", "#e31a1c","#33a02c" ,
       "#b2df8a","#b2df8a", "#b2df8a", "#b2df8a","#b2df8a","#b2df8a","#b2df8a","#b2df8a")
n<-c("#1f78b4","#ff7f00", "#fdbf6f", "#e31a1c","#33a02c" ,
     "#b2df8a","#b2df8a", "#b2df8a", "#b2df8a","#b2df8a","#b2df8a","#b2df8a","#b2df8a")
names(col)<-n
titles<-c("LINE/CR1", "Low complexity", "LTR", "No match", "Simple repeat")
n<-c(1,2,3,4,5)
names(titles)<-n

pg$data[[1]] %>% mutate(chr_nr = x+0.5) -> temp
temp$group<-factor(temp$group,levels= c(1,3,2,5,4))

temp %>%
  ggplot(aes(as.factor(chr_nr), y=density, fill=fill)) + geom_bar(stat= "identity") +
  theme_classic(base_size = 9) +
  ylab("Frequency")+
  scale_x_discrete(labels=round(seq(2,8,2)/16, digits=2))+
  scale_fill_manual(values = col) +
  theme(plot.margin = unit(c(5,5,5,5),"mm"),
        panel.spacing.x = unit(2,"mm"),
        title = element_text(),
        legend.position="none",
```

```
        axis.title.x=element_blank(),
        axis.text.x=element_text(size=9, angle=75, vjust=0.5),
        axis.title.y=element_text(),
        strip.text.x = element_text( margin=unit(c(2,2,2,2),"mm")),
        strip.background = element_blank())+
  facet_grid(. ~ group,
             labeller=labeller(group = titles),
             scale="free_x",
             space="free_x") ->sfs_hc_indels

filtered_table %>% filter(type == "INV") %>%
  filter(SV_ID %in% crow_clade_variants) %>% filter(individual %in% hc) %>%
  group_by(SV_ID) %>% summarize(freq=sum(genotype)) %>%
  filter(freq!=0 & freq!=16) %>% mutate(folded_freq=ifelse(freq>=9,16-freq,freq)) %>%
  ggplot(aes(folded_freq, stat(density))) + geom_histogram(binwidth=2) +
  theme_classic(base_size = 9) +
  ylab("Frequency")+
  theme(plot.margin = unit(c(5,5,5,5),"mm"),
        panel.spacing.x = unit(2,"mm"),
        title = element_text(),
        legend.position="none",
        axis.title.x=element_blank(),
        axis.text.x=element_text(angle=45),
        axis.title.y=element_text(),
        strip.text.x = element_text( margin=unit(c(2,2,2,2),"mm")),
        strip.background = element_blank()) -> plot
ggplot_build(plot) -> pg

pg$data[[1]] %>% mutate(chr_nr = x+0.5) -> temp

temp %>%
  ggplot(aes(as.factor(chr_nr), y=density)) + geom_bar(stat= "identity") +
  theme_classic(base_size = 9) +
  scale_x_discrete(labels=round(seq(2,8,2)/16, digits=2))+
  ggtitle("Inversion") +
  theme(plot.margin = unit(c(5,5,5,2),"mm"),
        panel.spacing.x = unit(2,"mm"),
        plot.title = element_text(size=9, hjust=0.5),
        legend.position="none",
        axis.title.x=element_blank(),
        axis.text.x=element_text(size=9, angle=75, vjust=0.5),
        axis.title.y=element_blank(),
        strip.text.x = element_text( margin=unit(c(2,2,2,2),"mm")),
        strip.background = element_blank())  -> sfs_hc_inversion

svg("sfs_hc_binned.svg",  width= 7, height=3)
grid.arrange(sfs_hc_indels, sfs_hc_inversion, ncol=2, widths=c(5,1))
dev.off()

### SFS plots for binned populations:

col<-c("#1f78b4","#ff7f00", "#fdbf6f", "#e31a1c","#33a02c" ,
       "#b2df8a","#b2df8a", "#b2df8a", "#b2df8a","#b2df8a","#b2df8a","#b2df8a","#b2df8a")
```

```
n<-c("no_hit","LTR", "Simple_repeat" , "Low_complexity" ,"LINE/CR1",
     "Other","Other","Other","Other","Other","Other" ,"Other" ,"Other")
names(col)<-n

filtered_table %>% filter(type != "INV") %>%
  filter(SV_ID %in% crow_clade_variants) %>% filter(individual %in% cc) %>%
  group_by(SV_ID) %>% summarize(freq=sum(genotype)) %>%
  filter(freq!=0 & freq!=16) %>% mutate(folded_freq=ifelse(freq>=9,16-freq,freq)) %>%
  join(., rm_output, by="SV_ID") %>%
  filter(repeat_class %in% four_most_common_repeat_classes) %>%
  ggplot(aes(folded_freq, stat(density), fill=repeat_class)) +
  geom_histogram(binwidth=2) +
  theme_classic(base_size = 9) +
  ylab("Frequency")+
  scale_fill_manual(values = col) +
  theme(plot.margin = unit(c(5,5,5,5),"mm"),
        panel.spacing.x = unit(2,"mm"),
        title = element_text(),
        legend.position="none",
        axis.title.x=element_blank(),
        axis.text.x=element_text(angle=45, vjust=0.5),
        axis.title.y=element_text(),
        strip.text.x = element_text( margin=unit(c(2,2,2,2),"mm")),
        strip.background = element_blank())+
  facet_grid(. ~ repeat_class,
             scale="free_x",
             space="free_x") -> plot
ggplot_build(plot) -> pg

col<-c("#1f78b4","#ff7f00", "#fdbf6f", "#e31a1c","#33a02c" ,
       "#b2df8a","#b2df8a", "#b2df8a", "#b2df8a","#b2df8a","#b2df8a","#b2df8a","#b2df8a")
n<-c("#1f78b4","#ff7f00", "#fdbf6f", "#e31a1c","#33a02c" ,
     "#b2df8a","#b2df8a", "#b2df8a", "#b2df8a","#b2df8a","#b2df8a","#b2df8a","#b2df8a")
names(col)<-n
titles<-c("LINE/CR1", "Low complexity", "LTR", "No match", "Simple repeat")
n<-c(1,2,3,4,5)
names(titles)<-n

pg$data[[1]] %>% mutate(chr_nr = x+0.5) -> temp
temp$group<-factor(temp$group,levels= c(1,3,2,5,4))

temp %>%
  ggplot(aes(as.factor(chr_nr), y=density, fill=fill)) + geom_bar(stat= "identity") +
  theme_classic(base_size = 9) +
  ylab("Frequency")+
  scale_x_discrete(labels=round(seq(2,8,2)/16, digits=2))+
  scale_fill_manual(values = col) +
  theme(plot.margin = unit(c(5,5,5,5),"mm"),
        panel.spacing.x = unit(2,"mm"),
        title = element_text(),
        legend.position="none",
        axis.title.x=element_blank(),
        axis.text.x=element_text(size=9, angle=75, vjust=0.5),
```

```
        axis.title.y=element_text(),
        strip.text.x = element_text( margin=unit(c(2,2,2,2),"mm")),
        strip.background = element_blank())+
  facet_grid(. ~ group,
             labeller=labeller(group = titles),
             scale="free_x",
             space="free_x") ->sfs_cc_indels

filtered_table %>% filter(type == "INV") %>%
  filter(SV_ID %in% crow_clade_variants) %>% filter(individual %in% cc) %>%
  group_by(SV_ID) %>% summarize(freq=sum(genotype)) %>%
  filter(freq!=0 & freq!=16) %>% mutate(folded_freq=ifelse(freq>=9,16-freq,freq)) %>%
  ggplot(aes(folded_freq, stat(density))) + geom_histogram(binwidth=2) +
  theme_classic(base_size = 9) +
  ylab("Frequency")+
  theme(plot.margin = unit(c(5,5,5,5),"mm"),
        panel.spacing.x = unit(2,"mm"),
        title = element_text(),
        legend.position="none",
        axis.title.x=element_blank(),
        axis.text.x=element_text(angle=45),
        axis.title.y=element_text(),
        strip.text.x = element_text( margin=unit(c(2,2,2,2),"mm")),
        strip.background = element_blank()) -> plot
ggplot_build(plot) -> pg

pg$data[[1]] %>% mutate(chr_nr = x+0.5) -> temp

temp %>%
  ggplot(aes(as.factor(chr_nr), y=density)) + geom_bar(stat= "identity") +
  theme_classic(base_size = 9) +
  scale_x_discrete(labels=round(seq(2,8,2)/16, digits=2))+
  ggtitle("Inversion") +
  theme(plot.margin = unit(c(5,5,5,2),"mm"),
        panel.spacing.x = unit(2,"mm"),
        plot.title = element_text(size=9, hjust=0.5),
        legend.position="none",
        axis.title.x=element_blank(),
        axis.text.x=element_text(size=9, angle=75, vjust=0.5),
        axis.title.y=element_blank(),
        strip.text.x = element_text( margin=unit(c(2,2,2,2),"mm")),
        strip.background = element_blank())  -> sfs_cc_inversion

svg("sfs_cc_binned.svg",  width= 7, height=3)
grid.arrange(sfs_cc_indels, sfs_cc_inversion, ncol=2, widths=c(5,1))
dev.off()
```

Short-read based SV detection

Illumina short reads were mapped with `BWA-MEM` and SV calling was performed on the bam files using `Delly`, `Lumpy` and `Manta`. The resulting vcf files were merged with `SURVIVOR`. Here are the representative commands:

```
# Delly
delly call -o delly_sv '_delly' -s 15 -q 20 -g reference mapped_reads.bam

# Lumpy
lumpy -mw 4 -tt 0.0 -pe bam_file:$out'.discordand.sort.bam',\
histo_file:$out'.hist',mean:$INS,stdev:$STD,read_length:$len, \
min_non_overlap:150,discordant_z:4,back_distance:20,weight:1, \
id:1,min_mapping_threshold:20  -sr bam_file:$out'splitters.sort.bam',\
back_distance:20,weight:1,id:2,min_mapping_threshold:20 > lumpy_sv.vcf

#Manta
out="output_Manta"
echo $out
#create Manta workflow
configManta.py --bam= mapped_reads.bam --referenceFasta= reference --runDir=$out'_Manta'
#run Manta workflow
python $out'_Manta'/runWorkflow.py -j 15 -m local -g 30
#combine results from Manta
gunzip  --stdout $out'_Manta/results/variants/candidateSV.vcf.gz' > $out'.manta.vcf'
gunzip  --stdout $out'_Manta/results/variants/diploidSV.vcf.gz' | \
grep -v '^#' >> $out'.manta.vcf'
gunzip  --stdout $out'_Manta/results/variants/candidateSmallIndels.vcf.gz'| \
grep -v '^#' >> $out'.manta.vcf'
```

FST analysis

We calculated FST for the LR variants using `vcftools`. First we removed duplicated variants (those with the exact same starting position), as those caused issues with `vcftools`.

```
grep -v '#' merged_filtered_force_called.vcf | \
   awk '{print $1 "/" $2}' | sort | uniq -c | awk '$1 == 1' | \
   awk '{print $2}' > ref

grep -v '#' merged_filtered_force_called.vcf | \
   tr ";" "\t"  | cut -f 1,2,5,10 | sed -e 's/SVLEN=//g'  | \
   sed -e 's/<//g' -e 's/>//g' | awk '{print $1 "/" $2 "\t" $0}' > temp
```

Small python script to join to files in order:

```
import csv
import sys
import io

input_1=sys.argv[1]
input_2=sys.argv[2]
input_dict={}

with open(input_1) as csvfile:
  inputfile = csv.reader(csvfile, delimiter='\t')
  for row in inputfile:
    key = row[0]
    value = row[0:4]
    input_dict[key] = value
```

```
with open(input_2) as csvfile:
  inputfile = csv.reader(csvfile, deliminter='\t')
  for row in inputfile:
    if row[0] in input_dict.keys():
      print input_dict[row[0]]
    else:
      pass
```

Use this script to sort remove duplicated variants.

```
python join_ref.py temp ref | sed -e 's/\[//g' -e 's/\]//g' -e 's/\,//g' |\
 tr "'" "X" | sed 's/X//g' | tr " " "\t" > ref_2019-09-16
```

Now calculate FST between all possible populations / species:

```
while IFS= read -r line; do
  pop1=$(echo $line | awk '{print $1}' )
  pop2=$(echo $line | awk '{print $2}' )
  echo $pop1 $pop2
  vcftools --vcf merged_filtered_force_called.vcf \
     --weir-fst-pop $pop1
     --weir-fst-pop $pop2
     --out ${pop1}_${pop2}.2019-09-16 \
     --max-missing 0.8
  tail -n +2 ${pop1}_${pop2}.2019-09-16.weir.fst | awk '{print $1 "/" $2 "\t" $3}' | \
  sort -k 1,1 | sed 's/-nan/NA/g' > temp
  python join.py temp ref_2019-09-16  | \
  cat <(echo 'fst_'$pop1'.'$pop2) - > ${pop1}_${pop2}.2019-09-16.out
  rm temp
done<combinations

cat <(echo 'SV_ID       chr     pos     type    len') ref_2019-09-16 | \
paste - *2019-09-16.out
```

This table is then used for the analysis in R:

```
fst<-read.table("fst_filtered_force_called_B03_excluded_HC_ref.2019-09-16.table", header=T)
fst %>% mutate_at( names(fst)[6:19], funs(ifelse(. < 0, 0, .))) -> temp
fst<-temp
fst %>% mutate(length=sqrt(len^2)) %>% select(-len) %>%
  mutate(len=length) %>% select(-length) -> temp
fst<-temp

# first look at the phenotypically divergent comparison of the
# German all black carrion crow and black-and-grey hooded crow:
fst %>% mutate(SV_ID = paste0(chr, "_" ,pos)) %>%
  filter(SV_ID %in% crow_clade_variants) %>% select(fst_HC.CC) %>%
  unlist() %>% as.vector() %>% quantile(0.99, na.rm=T) -> ninetyninth_percentile_fst_HC.CC
fst %>% mutate(SV_ID = paste0(chr, "_" ,pos)) %>%
  filter(SV_ID %in% crow_clade_variants) %>%
  filter(fst_HC.CC > ninetyninth_percentile_fst_HC.CC ) %>%
  arrange(desc(fst_HC.CC)) %>% select(chr, pos, type, len, fst_HC.CC) %>%
  mutate(SV_ID =paste0(chr, "_", pos)) -> ninetyninth_percentile_outlier_variants_HC_CC
write.table(ninetyninth_percentile_outlier_variants_HC_CC,
            file="ninetyninth_percentile_outlier_variants_HC_CC.txt",
```

```
            sep="\t", quote=F, row.names = F)

# As a control, compare within within phenotype (black German carrion vs. black Spanish carrion):
fst %>% mutate(SV_ID = paste0(chr, "_" ,pos)) %>%
  filter(SV_ID %in% crow_clade_variants) %>%
  select(fst_CC.CCE) %>% unlist() %>% as.vector() %>%
  quantile(0.99, na.rm=T) -> ninetyninth_percentile_fst_CC.CCE
fst %>% mutate(SV_ID = paste0(chr, "_" ,pos)) %>%
  filter(SV_ID %in% crow_clade_variants) %>%
  filter(fst_CC.CCE > ninetyninth_percentile_fst_CC.CCE ) %>%
  arrange(desc(fst_CC.CCE)) %>%
  select(chr, pos, type, len, fst_CC.CCE) %>%
  mutate(SV_ID =paste0(chr, "_", pos)) -> ninetyninth_percentile_outlier_variants_CC_CCE
write.table(ninetyninth_percentile_outlier_variants_CC_CCE,
            file="ninetyninth_percentile_outlier_variants_CC_CCE.txt", sep="\t")

# Now look at the overlap HC.CC vs. CC.CCE:

ninetyninth_percentile_outlier_variants_HC_CC %>%
  merge(., ninetyninth_percentile_outlier_variants_CC_CCE) %>%
  select(SV_ID) %>% unlist() %>% as.vector() -> overlap_HC.CC_CC.CCE

ninetyninth_percentile_outlier_variants_HC_CC %>%
  filter(SV_ID %not_in% overlap_HC.CC_CC.CCE) ->
  ninetyninth_percentile_outlier_variants_HC_CC_filtered
write.table(ninetyninth_percentile_outlier_variants_HC_CC_filtered,
            "ninetyninth_percentile_outlier_variants_HC_CC_filtered.txt", quote=F, row.names = F)
```

Gene expression of the *NDP* gene

Here we took gene expression data from *Poelstra et al. 2019* and looked at the expression of the *NDP* gene in relation to the LTR element insertion genotype.

```
gene_counts<-read.table("transformed_gene_counts", header=T)
col<-c("#bdbdbd", "#636363", "#1f78b4", "#b2df8a", "#33a02c")
name<-c("HC", "CC",0,1,2)
names(col)<-name

# Linear model:
gene_counts %>% filter(part == "body") %>%
  lm(normalized_count ~ -1 + factor(gt), data= . ) -> mod

summary(mod)

svg("Normalized_gene_counts_NDP.svg", height=3, width=3)
gene_counts %>% filter(part=="body") %>%
   filter(gt != "NA") %>%
   ggplot(aes(as.factor(gt), normalized_count, fill=as.factor(gt))) +
   theme_classic(base_size = 9) +
   geom_boxplot() +
   scale_fill_manual(values=col) +
   ylab("Normalized gene count")+
```

```
  scale_x_discrete(breaks=c("0", "1", "2"),
                   labels=c("Homozygous insertion",
                            "Heterozygous",
                            "Homozygous non-insertion")) +
theme(
  legend.position="none",
  axis.text.x = element_text(size = 9),
      axis.title.x=element_blank())
dev.off()
```

Repeat characterization pipeline

To assign insertion / deletion sequences to known repeats, we developed a pipeline which uses `RepeatMasker`. In the following `Makefile` all steps are automized:

```
SHELL := /bin/bash
MAKEFLAGS += --no-builtin-rules
.SUFFIXES:
VPATH := ./vcfs
VCFS  := $(notdir $(shell ls ${VPATH}/*.vcf.gz))
RLIB  := fAlb_lycPyr_hcrow_aves_combined.lib
#cores to run repeat masker on
#can not be empty
CORES := 10
.DELETE_ON_ERROR:
.PHONEY:All
All: \
    ${VCFS:.vcf.gz=.info}       \
    ${RLIB:=.fai}               \
    info_joined.txt             \
    for_repeatmasker.fasta      \
    for_repeatmasker.fasta.fai  \
    for_repeatmasker.key        \
    for_repeatmasker.fasta.out  \
    for_repeatmasker.tab        \
    matrix.res                 \
    filtered_matrix.res       \
    filtered_persite_matrix.res
for_repeatmasker.fasta for_repeatmasker.key:$@
%.info:%.vcf.gz
    #getting SEQ field from file $< using bcftools
    echo -e "CHROM\tPOS\tREF\tALT\tSEQ_$(notdir ${<:.vcf.gz=})" > $@
    bcftools query -f '%CHROM\t%POS\t%REF\t%ALT\t%SEQ\n' $< >> $@
info_joined.txt:$(VCFS:.vcf.gz=.info)
    #pasting and parsing to get sequences in SEQ fields of all files
    echo $^ |sed -s 's/ / \/dev\/null /g' |xargs paste -d $$'\t' > $@
for_repeatmasker.fasta for_repeatmasker.key:get_seq_from_vcf.pl info_joined.txt
    #generating fasta and key files from $(word 2,$^)
    perl $^ $(basename $@)
%.fasta.out:%.fasta  $(RLIB)
    #run repeat masker here on $< with library file ${RLIB}
    RepeatMasker -no_is -pa ${CORES}  -lib $(word 2,$^) $<
```

```
%.tab:rmparse.pl %.fasta.out %.fasta.fai ${RLIB:=.fai}
	#parsing repeatmasker output file $< using bioperl
	perl $^ > $@
matrix.res:matmaker.pl for_repeatmasker.tab for_repeatmasker.key info_joined.txt
	#generating tabular multiple sample repeat annottation based
	perl $^  > $@
filtered_matrix.res:matfilter.pl matrix.res
	perl $^ > $@
filtered_persite_matrix.res:persite_matfilter.pl matrix.res
	perl $^ > $@
%.fai:%
	#fai indexing using samtools
	samtools faidx $<
clean:
	rm -f ${VCFS:.vcf.gz=.info}       \
	info_joined.txt                   \
	for_repeatmasker.fasta            \
	for_repeatmasker.key              \
	for_repeatmasker.fasta.out        \
	for_repeatmasker.fasta.cat.gz     \
	for_repeatmasker.fasta.masked     \
	for_repeatmasker.tab              \
	for_repeatmasker.fasta.tbl        \
	matrix.res                        \
	filtered_matrix.res               \
	filtered_persite_matrix.res
```

Depending on `gnuparallel`, `BioPerl`, `RepeatMasker` and `samtools`, the pipeline was run with `make`.
